# Characterization and clinical use of inflammatory cerebrospinal fluid protein markers in Alzheimer’s disease

**DOI:** 10.1186/s13195-018-0353-3

**Published:** 2018-02-26

**Authors:** Frederic Brosseron, Andreas Traschütz, Catherine N. Widmann, Markus P. Kummer, Pawel Tacik, Francesco Santarelli, Frank Jessen, Michael T. Heneka

**Affiliations:** 1 German Center for Neurodegenerative Diseases (DZNE), Bonn, Germany; 2 Department of Neurodegenerative Diseases & Geropsychiatry/Neurology, University of Bonn Medical Center, Bonn, Germany; 3 Department of Psychiatry, University of Cologne, Medical Faculty, Kerpener Strasse 62, 50924 Cologne, Germany

**Keywords:** Alzheimer’s disease, Mild cognitive impairment, Cerebrospinal fluid, Biomarker, Inflammation, Discriminative power

## Abstract

**Background:**

Neuroinflammation has gained increasing attention as a potential contributing factor in Alzheimer’s disease (AD) pathology. A clinical cerebrospinal fluid biomarker capable of monitoring this process during the course of the disease has yet to emerge, chiefly owing to contradictory research findings. In this study, we sought to clarify the utility of inflammatory biomarkers in diagnostic procedures of AD in three steps: (1) to screen for proteins that are robustly detectable in cerebrospinal fluid; (2) based on this analysis, to explore any associations between the analytically robust markers and salient pathological features of AD; and (3) to determine the discriminative power of these markers in the clinical diagnosis of AD.

**Methods:**

From a total of 46 proteins, 15 that were robustly detectable in cerebrospinal fluid were identified. A subsequent analysis of these markers in a cohort of 399 patients (nondemented subjects, patients with mild cognitive impairment [MCI], and patients with AD, supplemented by smaller cohorts of other diseases) was conducted. Fluid biomarker data were related to AD pathology and neuropsychological markers and adjusted for confounders such as age, sex, apolipoprotein E genotype, and biobank storage time.

**Results:**

Cerebrospinal fluid levels of C-reactive protein and soluble TREM2 differed between nondemented subjects, patients with MCI, or patients with AD and were associated with amyloid and tau pathology. Several markers were associated with tau pathology only or with other neurodegenerative diseases. Correlations between neuropsychological performance and inflammatory markers were weak, but they were most prominent in AD and for the most challenging cognitive tests. All investigated covariates had significant influence, with varying effects across the markers. Still, none of the markers achieved discriminative power of more than 70% to distinguish between patient groups defined by clinical or neuropathological categories.

**Conclusions:**

Basic analytical considerations proved indispensable for this type of study because only one-third of the tested markers were robustly detectable in cerebrospinal fluid. Detectable inflammatory protein markers were associated in multiple ways with AD pathology. Yet, even significantly associated markers were not powerful enough in terms of effect strength, sensitivity, and specificity, and hence they were not suited for direct use in clinical diagnostic practice. Targets other than those most commonly considered in this field of research might provide results with better clinical applicability.

**Electronic supplementary material:**

The online version of this article (10.1186/s13195-018-0353-3) contains supplementary material, which is available to authorized users.

## Background

Neuroinflammation is now widely accepted as a pathological hallmark of Alzheimer’s disease (AD) and other dementias [1, 2]. However, in contrast to the classical fluid biomarker hallmarks of amyloid and tau proteins, a standard clinical application of inflammatory markers in the clinical diagnosis of AD is lacking, likely owing to contradictory and heterogeneous findings of numerous studies [3–6]. It is noteworthy that several frequently investigated inflammatory markers are found in low abundance in both brain tissue and cerebrospinal fluid (CSF). These have therefore been excluded from analysis in some more recent studies [7, 8]. Hence, part of the discrepancy may be due to marker levels being close to detection thresholds in laboratory assays, yielding nonreproducible findings. Another important aspect of such biomarker research is the attainment of sufficient sensitivity and specificity for diagnostic application [9]. Although high sensitivity and specificity have consistently been reported for the use of CSF amyloid or tau markers, these key diagnostic parameters have barely been studied for inflammatory markers in AD research.

To address these issues, we first analyzed a total of 46 inflammation-associated protein markers to validate assay performance in CSF. The panel was based on a commercial 40-plex previously used by Chen et al*.* for a study with a similar concept but involving an investigation of brain samples containing several typical pro- and anti-inflammatory cytokines and chemokines as well as mediators likewise associated with vascular injury [8]. Additionally, the following markers from the complement system’s classical and alternative pathways, genome-wide association studies, and our own previous research that have previously been related to AD were selected: C1q, C5a, C3aDesArg, soluble triggering receptor expressed on myeloid cells 2 (sTREM2), inhibition of soluble interleukin-1 (IL-1) receptor accessory protein (sIL-1RAcP), and myeloid-related proteins 8/14 [10–14]. Afterward, a panel of 15 markers with robust detectability in CSF was used to study samples derived from patients in a neurological and psychiatric outpatient unit, where decision-making relies on the ability of biomarkers to distinguish AD from other disorders. Investigated cohorts included patients with a diagnosis of AD or mild cognitive impairment (MCI) and nondemented comparator subjects (nondemented neurological patients without cognitive dysfunction, central nervous system [CNS] involvement, or inflammation-associated disorders), supplemented by smaller groups of subjects with other neurodegenerative diseases. We tested for associations between inflammatory markers and pathological and neuropsychological features as well as major potentially confounding factors. Finally, the diagnostic performance was evaluated by comparing the discriminative power (sensitivity and specificity) of significant inflammatory markers with that of standard AD amyloid and tau biomarkers to discriminate across the three groups in pairwise comparisons. This article is supported by Additional file 1, which provides detailed results of the initial test phase for each of the 46 proteins and a statistical supplement reporting those test results not included in the body of the article.

## Methods

### Subjects

A total of 399 CSF samples were retrieved from the biobank of the Department of Neurodegenerative Diseases & Geropsychiatry/Neurology, German Center for Neurodegenerative Diseases (Bonn, Germany). Patients had undergone lumbar puncture as part of clinical diagnostic procedures. Informed consent for research purposes was obtained with local ethics committee approval (University Hospital of Bonn Ethics Commission 279/10). Diagnostic procedures were carried out according to the criteria of the National Institute of Neurological and Communicative Disorders and Stroke/Alzheimer’s Disease and Related Disorders Association. Levels of the CSF AD core biomarkers β-amyloid 1–40 (Aβ_40_), β-amyloid 1–42 (Aβ_42_), total tau (t-tau), and tau phosphorylated at position 181 (p-tau-181), as well as the Aβ_42_/Aβ_40_ ratio, were considered in the diagnostic process, for which laboratory-specific cutoff values were employed (Aβ_42_ < 350 pg/ml, Aβ_42_/Aβ_40_ ratio < 0.07, t-tau > 450 pg/ml, p-tau-181 > 56 pg/ml). Clinical and apolipoprotein E (*ApoE*) data were obtained from the clinic for neurodegenerative diseases at the Department of Neurology and Psychiatry Outpatient Unit for Neurodegenerative Diseases (www.kbfz.de) at the University of Bonn, Germany. Neuropsychological tests (NPTs) were performed as clinically indicated and comprised the Mini Mental State Examination and the Consortium to Establish a Registry for Alzheimer’s Disease battery, supplemented by the Trail Making Test A and B and the Free and Cued Selective Reminding Test, as described elsewhere [15–17].

Clinical cohorts included diagnoses of AD, MCI, Parkinson’s disease (PD), dementia with Lewy bodies (DLB), frontotemporal dementia (FTD), and amyotrophic lateral sclerosis (ALS). For details, *see* Table 1 and Additional file 2. The AD cohort included patients with “probable Alzheimer’s disease” according to National Institute on Aging-Alzheimer’s Association criteria, supported by at least an intermediate level of evidence for AD pathology by CSF analysis or imaging [18]. The MCI cohort included patients with clinical or neuropsychological evidence of cognitive decline below age-dependent norms but with preserved daily function. For comparison purposes, samples from nondemented neurological patients without cognitive dysfunction, CNS involvement, or inflammation-associated disorders were used. The nondemented cohort consisted of 85 patients with diverse clinical diagnoses (*see* Additional file 2 for details). In the following discussion, the large nondemented, MCI, and AD cohorts will be designated as “main cohorts,” whereas other dementias with numbers of 20 or fewer patients will be designated as “supplemental cohorts.” Individuals with a history of brain surgery or trauma, fever, acute infections, recent sepsis, or other inflammation-associated disorders were excluded. Furthermore, samples were excluded when CSF was turbid or contaminated with blood (erythrocyte count > 1000/μl).

**Table 1:** Descriptive data of the main clinical cohorts

	Feature (median ± SD, minimum–maximum, coverage)			
	Nondemented (ND)	Mild cognitive impairment (MCI)	Alzheimer’s disease (AD)	*p* Value (significant cohort-wise comparisons)
No. of subjects	85	130	116	–
Age, years	67 ± 11	71 ± 8	74 ± 8	**1 × 10E** ^**−10**^
	43–81; 100%	48–85; 100%	50–88; 100%	(ND vs. MCI, AD)
Sex, % male	65	65	45	**χ** ^**2**^ **= 2 × 10E** ^**−3**^
				(AD vs. ND, MCI)
Storage time, months	27 ± 21	36 ± 23	22 ± 19	**7 × 10E** ^**−5**^
	0.3–95.0; 100%	4.0–91.0; 100%	4.0–94.0; 100%	(MCI vs. ND, AD)
Aβ_42_, pg/ml	552 ± 315	432 ± 302	319 ± 191	**1 × 10E** ^**−9**^
	138–1653; 100%	87–1326; 100%	91–1505; 100%	(All cohorts)
Aβ_42_/Aβ_40_ ratio	0.10 ± 0.03	0.07 ± 0.04	0.05 ± 0.02	**1 × 10E** ^**−23**^
	0.02–0.25; 100%	0.02–0.23; 100%	0.03–0.15; 100%	(All cohorts)
t-tau, pg/ml	289 ± 228	419 ± 290	680 ± 344	**3 × 10E** ^**−14**^
	36–1210; 100%	71–1528; 100%	126–2157; 100%	(All cohorts)
p-tau-181, pg/ml	43 ± 25	59 ± 30	76 ± 36	**1 × 10E** ^**−12**^
	16–157; 100%	18–158; 100%	23–248; 100%	(All cohorts)
Aβ_42_/p-tau-181 ratio	17.0 ± 9.2	9.7 ± 9.1	4.5 ± 3.8	**6 × 10E** ^**−27**^
	2.0–42.8; 100%	1.5–48.8; 100%	1.3–25.2; 100%	(All cohorts)
CSF protein, mg/L	488 ± 167	458 ± 148	453 ± 144	0.423
	257–999; 93%	248–1090; 98%	245–1055; 99%	
CSF albumin, mg/L	268 ± 137	256 ± 116	260 ± 116	0.485
	103–688; 88%	100–724; 98%	118–803; 98%	
CSF IgG, mg/L	31 ± 17	28 ± 13	28 ± 16	0.263
	8–94; 88%	9–108; 98%	11–128; 98%	
CSF leukocytes, cells/μl	1 ± 2	1 ± 1	1 ± 1	0.942
	0–11; 93%	0–7; 98%	0–7; 99%	
MMSE score	29 ± 1	26 ± 3	21 ± 4	**1 × 10E** ^**−48**^
	27–30; 73%	18–30; 91%	6–29; 96%	(All cohorts)
Education, years	15 ± 2	14 ± 4	13 ± 4	0.129
	11–18; 18%	8–22; 82%	8–22; 72%	
Word list learning	20 ± 3	16 ± 4	13 ± 4	**1 × 10E** ^**−11**^
	16–23; 18%	5–27; 81%	3–19; 69%	(All cohorts)
Word list recall	6 ± 1	4 ± 2	2 ± 2	**6 × 10E** ^**−16**^
	4–9; 18%	0–10; 78%	0–7; 60%	(All cohorts)
Word list discrimination, %	100 ± 3	95 ± 15	85 ± 21	**7 × 10E** ^**−6**^
	90–100; 18%	0–100; 81%	0–100; 69%	(All cohorts)
Figure recall	8 ± 2	5 ± 3	3 ± 3	**4 × 10E** ^**−10**^
	2–11; 16%	0–11; 81%	0–10; 60%	(All cohorts)
TMT A	52 ± 27	57 ± 33	84 ± 46	**4 × 10E** ^**−3**^
	20–119; 16%	26–180; 80%	29–180; 69%	(AD vs. ND, MCI)
TMT B	115 ± 50	166 ± 80	300 ± 71	**5 × 10E** ^**−11**^
	53–245; 16%	52–300; 75%	76–300; 60%	(All cohorts)
Semantic fluency	22 ± 7	16 ± 6	12 ± 5	**2 × 10E** ^**−10**^
	14–39; 18%	5–34; 81%	3–26; 72%	(All cohorts)
Phonetic fluency	12 ± 4	10 ± 5	9 ± 4	**7 × 10E** ^**−3**^
	7–19; 16%	1–26; 79%	2–22; 70%	(MCI vs. AD)
FCSRT free	32 ± 8	19 ± 8	9 ± 8	**2 × 10E** ^**−12**^
	14–40; 14%	0–40; 63%	0–33; 42%	(All cohorts)
FCSRT summary score	48 ± 3	47 ± 8	36 ± 15	**6 × 10E** ^**−10**^
	45–57; 14%	0–48; 63%	2–48; 42%	(AD vs. ND, MCI)

Boldface values highlight statistically significant results

*Abbreviations*: Aβ_*40*_ β-Amyloid 1–40, *Aβ*_*42*_ β-Amyloid 1–42, *AD* Alzheimer’s disease, *CSF* Cerebrospinal fluid, *FCSRT* Free and Cued Selective Reminding Test, *IgG* Immunoglobulin G, *MCI* Mild cognitive impairment, *MMSE* Mini Mental State Examination, *ND* Nondemented, *p-tau-181* Tau phosphorylated at position 181, *TMT* Trail Making Test, *t-tau* Total tau

### Protein analysis

For AD core biomarkers, we used the V-PLEX Aβ Peptide Panel 1 (6E10) Kit (K15200E) and the V-PLEX Human Total Tau Kit (K151LAE) (Mesoscale Diagnostics LLC, Rockville, MD, USA), as well as the INNOTEST PHOSPHO TAU(181P) kit (81581, Fujirebio, Ghent, Belgium). For inflammation-associated proteins, detailed information is provided in Additional file 1.

In the initial evaluative step, all inflammatory markers were classified as detectable, undetectable, or borderline. The last group contained markers at detection limits for which test results suggested that addition of a known dose of protein to the sample could be used to improve the assay signal (spike-in concept). A full record of this test phase is provided in Additional file 1.

CSF samples saved strictly for research purposes had been snap-frozen in liquid nitrogen and stored in a biobank at −80 °C. Prior to assay, samples were thawed on ice and divided into aliquots (total of two freeze-thaw cycles). All further sample processing was conducted on ice until samples were applied to the assays. Samples and calibrators were run in duplicates, and samples with a coefficient of variation (CV) > 20% were repeated. To normalize for interrun variance, a pooled and aliquoted CSF sample was run as an internal standard on each assay plate.

### Statistical analysis

Statistical analysis and data visualization were performed using the Prism 7 (GraphPad Software Inc., La Jolla, CA, USA), IBM SPSS Statistics 21 (IBM Corporation, Armonk, NY, USA), and R (R Foundation for Statistical Computing, Institute for Statistics and Mathematics, Wirtschaftsuniversität Wien, Vienna, Austria) software programs. Protein concentrations were normalized to an internal standard sample to address interrun variances. Data distribution was analyzed using the D’Agostino-Pearson omnibus test and quantile-quantile plots. Some samples showed extreme values without indication of inflammatory disorders or other exclusion criteria in medical records (*see* Additional file 2). For this reason, and because none of the protein data were normally distributed, nonparametric tests were employed. Group comparisons and correlation analyses were performed using the Mann-Whitney *U* test, Kruskal-Wallis test, and Spearman’s rank linear correlation. Discriminative power was calculated from the intersection points of sensitivity and specificity based on ROC and Youden index (power = sensitivity = specificity). For tests assuming normally distributed data (principal component analysis [PCA], linear regression analysis, and analysis of covariance [ANCOVA]), protein-level data were log-transformed prior to testing. Intercohort distribution of sex was assessed using Pearson’s chi-square test. Statistical significance was defined as α = 0.05, and the Bonferroni method was used to compensate for multiple pairwise comparisons. Samples with missing values were excluded from analysis of respective features (i.e., for correlations with NPT, only samples with available data were considered, depending on data coverage as described in Table 1). Additional file 2 contains a full report of those results not included in this article.

## Results

### Assay performance

Of 46 initially tested inflammation-associated markers, 14 met the criteria for reliable detection in CSF. Twenty-two were classified as undetectable, and 10 were at the borderline of detectability. The final panel consisted of 12 robust analytes (C1q, C3aDesArg, C-reactive protein [CRP], soluble intercellular adhesion molecule 1 [sICAM-1], sIL-1RAcP, IL-8, interferon-γ-induced protein 10 [IP-10], monocyte chemoattractant protein-1 [MCP-1], macrophage migration inhibitory factor [MIF], serum amyloid A protein [SAA], sTREM2, and soluble vascular cell adhesion molecule 1 [VCAM-1]) as well as 3 borderline candidates (vascular endothelial growth factor [VEGF], soluble VEGF receptor [VEGFR], and IL-6) for which the spike-in setup was tested (*see* Additional file 1). For all 15 proteins in the final panel, mean assay CVs were within a range of 1.5–5.7%. Protein levels followed a skewed data distribution; CRP and SAA were distributed on a lognormal scale. Protein levels were within the detection range in all tested samples, with the exception of the following: VEGF (2 undetectable samples), IL-6 (18 samples), and C3aDesArg (1 sample). The respective values were included as 0 pg/ml in data analysis.

### Inflammatory markers in nondemented, mild cognitive impairment, and Alzheimer’s disease

**Fig. 1:**
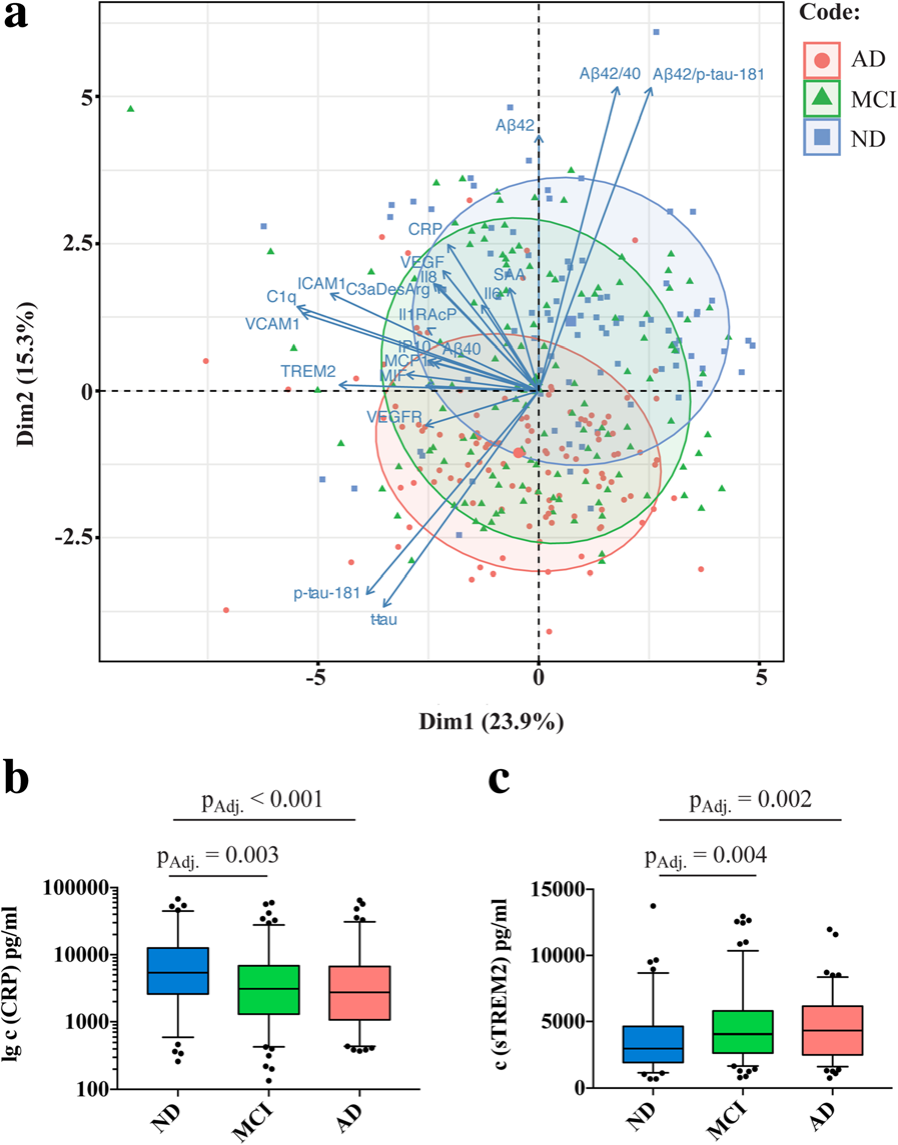
Biomarker distribution in the main cohorts. **a** Principal component analysis of cerebrospinal fluid levels of standard Alzheimer’s disease (AD) biomarkers and inflammatory proteins in nondemented subjects (ND), patients with mild cognitive impairment (MCI), and patients with AD. The first two dimensions account for approximately 24% and 15% of the variance in the dataset, respectively. CIs were set at 0.7. The standard amyloid and tau markers and their ratios were the main separators of the clinical cohorts. In contrast, inflammatory markers did not achieve comparable discriminatory strength. **b**, **c** In direct comparisons, only soluble triggering receptor expressed on myeloid cells 2 (sTREM2) and C-reactive protein (CRP) differed significantly between the nondemented cohort on the one hand and patients with MCI as well as patients with AD on the other. Box plots represent median and IQR, 5% and 95% percentile whiskers, and all data points outside that range. *Aβ*_*40*_ β-Amyloid 1–40, *Aβ*_*42*_ β-Amyloid 1–42, *IP-10* Interferon-γ-induced protein 10, *MCP-1* Monocyte chemoattractant protein 1, *MIF* Macrophage migration inhibitory factor, *p-tau-181* Tau phosphorylated at position 181, *SAA* Serum amyloid A protein, *sICAM-1* Soluble intercellular adhesion molecule 1, *sIL-1RAcP* Soluble inhibition of soluble interleukin-1 receptor accessory protein, *sVCAM-1* Soluble vascular cell adhesion molecule 1, *t-tau* Total tau, *VEGF* Vascular endothelial growth factor, *VEGFR* Vascular endothelial growth factor receptor

An overview of the features of the main cohorts (i.e., nondemented subjects, patients with MCI, patients with AD) is given in Table 1. As visualized by PCA analysis (Fig. 1a), the markers of the inflammatory panel were weaker differentiators of the main cohorts than standard AD amyloid and tau biomarkers. On the first PCA dimension, which accounted for 23.9% of the variance, inflammatory markers such as sTREM2, C1q, sVCAM-1, and sICAM-1 were the strongest contributing factors (visualized by the respective vectors). However, there was no separation of the main cohorts along this first PCA dimension. In contrast, cohorts were separated by the second dimension, which accounted for less of the overall variance (15.3%) but was influenced primarily by AD markers such as Aβ_42_, tau isoforms, or their ratios (visualized by the larger vectors of the AD markers).

When compared directly between the main cohorts, the levels of sTREM2 and CRP were significantly different (Kruskal-Wallis *p* < 0.001) (Fig. 1b and c, Table 2, and Additional file 2). Compared with the nondemented cohort, sTREM2 levels were higher and CRP levels were lower in the MCI and AD cohorts. CRP was also the only inflammatory marker significantly associated with the *ApoE* genotype, the most influential genetic AD risk factor [19]. The median CRP level dropped as the number of *ApoE* ε4 (*ApoE4*) alleles increased (no *ApoE4* alleles 3770 pg/ml, one allele 2600 pg/ml, two alleles 2052 pg/ml; Kruskal-Wallis *p* = 0.009), with the difference between homozygous ApoE4 carriers and noncarriers being the driving force (pairwise adjusted *p* = 0.014). For sTREM2, there was a trend of an association with *ApoE4* status or allele number (Kruskal-Wallis *p* = 0.071).

**Table 2:** Overview of significant group-wise comparison fold changes

Protein	MCI vs. ND	AD vs. ND	Amyloid-positive vs. amyloid-negative	Tau-positive vs. tau-negative
VEGF	n.s.	n.s.	0.9	0.9
sVEGFR-1	n.s.	n.s.	1.1	1.3
MCP-1	n.s.	n.s.	n.s.	1.1
IP-10	n.s.	n.s.	n.s.	1.2
IL-6	n.s.	n.s.	n.s.	n.s.
IL-8	n.s.	n.s.	n.s.	n.s.
SAA	n.s.	n.s.	n.s.	n.s.
CRP	0.6	0.5	0.5	0.7
sICAM-1	n.s.	n.s.	1.1	1.1
sVCAM-1	n.s.	n.s.	1.1	1.2
MIF	n.s.	n.s.	n.s.	1.3
C1q	n.s.	n.s.	n.s.	1.3
C3aDesArg	n.s.	n.s.	n.s.	n.s.
sIL-1RAcP	n.s.	n.s.	1.1	1.1
sTREM2	1.4	1.4	1.4	1.7

*Abbreviations: Aβ*_*40*_ β-Amyloid 1–40, *Aβ*_*42*_ β-Amyloid 1–42, *AD* Patients with Alzheimer’s disease, *CRP* C-reactive protein, *IL* Interleukin, *IP-10* Interferon-γ-induced protein 10, *MCI* Patients with mild cognitive impairment, *MCP-1* Monocyte chemoattractant protein 1, *MIF* Macrophage migration inhibitory factor, *ND* Nondemented subjects, *SAA* Serum amyloid A protein, *sICAM-1* Soluble intercellular adhesion molecule 1, *sIL-1RAcP* Soluble inhibition of soluble interleukin-1 receptor accessory protein, *sTREM2* Soluble triggering receptor expressed on myeloid cells 2, *sVCAM-1* Soluble vascular cell adhesion molecule 1 factor, s*VEGFR-1* Soluble vascular endothelial growth factor receptor 1, *VEGF* Vascular endothelial growth

The table lists only significant comparisons and only fold changes of median values. Insignificant changes are marked as not significant (n.s.). Amyloid pathology was defined by Aβ_42_/Aβ_40_ ratio; tau pathology was defined by total-tau. For detailed descriptions of median values, SDs, and ranges, *see* Additional file 2, page 8 and following)

### Association between inflammatory markers and Alzheimer’s disease pathology

**Fig. 2:**
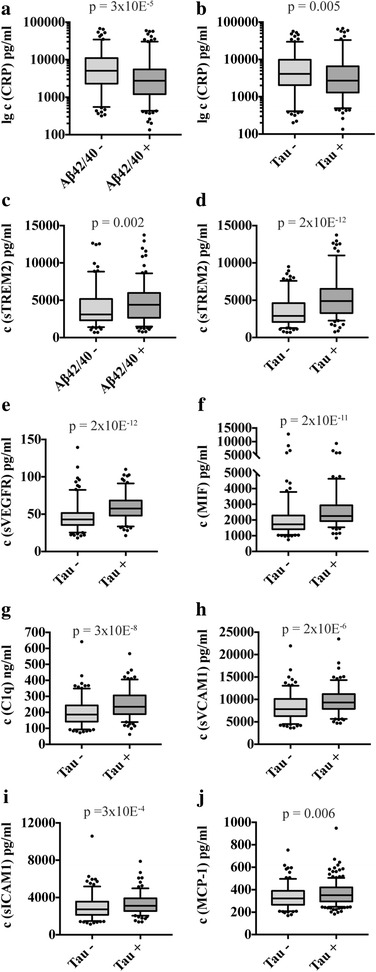
Association of inflammatory markers with amyloid and tau levels. The figure shows the ten strongest associations when dichotomizing the main cohorts by pathological (+) and nonpathological (−) levels of the β-amyloid 1–42 (Aβ_42_)/β-amyloid 1–40 (Aβ_40_) ratio or total tau (t-tau) (Mann-Whitney *U* test). *See* Additional file 2 for weaker or redundant associations. C-reactive protein (CRP) and soluble triggering receptor expressed on myeloid cells 2 (sTREM2) were associated with both amyloid and tau pathology (**a–d**). Other markers were predominantly associated with pathological tau levels, including (**e**) soluble vascular endothelial growth factor receptor (sVEGFR), (**f**) macrophage migration inhibitory factor (MIF), (**g**) C1q, (**h**) soluble vascular cell adhesion molecule 1 (sVCAM-1), (**i**) soluble intercellular adhesion molecule 1 (sICAM-1), and (**j**) monocyte chemoattractant protein 1 (MCP-1). Box plots represent median and IQR, 5% and 95% percentile whiskers, and all data points outside that range

To test the association of the inflammatory markers with standard biomarkers of AD pathology, the main cohorts were dichotomized on the basis of pathological levels of amyloid or tau biomarkers. The proteins VEGF, sVEGFR-1, MCP-1, IP-10, CRP, sICAM-1, sVCAM-1, MIF, C1q, sIL-1RaCP, and sTREM2 all varied according to pathological vs. nonpathological AD marker levels. The ten most significant findings are depicted in Fig. 2 (*see* Additional file 2 for complete results). Among these, only CRP and sTREMs were discriminated between both CSF amyloid and tau status. Other markers were predominantly associated with the presence of tau pathology. Complementary to this finding, there were several significant correlations between amyloid or tau markers and those inflammatory proteins that showed significance in cohort-wise comparisons. Most striking were the correlations of sVEGFR-1, sVCAM-1, MIF, C1q, or sTREM2 with tau or p-tau-181 that reached moderate strength (Spearman correlation *p* < 0.05, *r* = 0.300–0.556).

### Association between inflammatory markers and clinical features

**Fig. 3:**
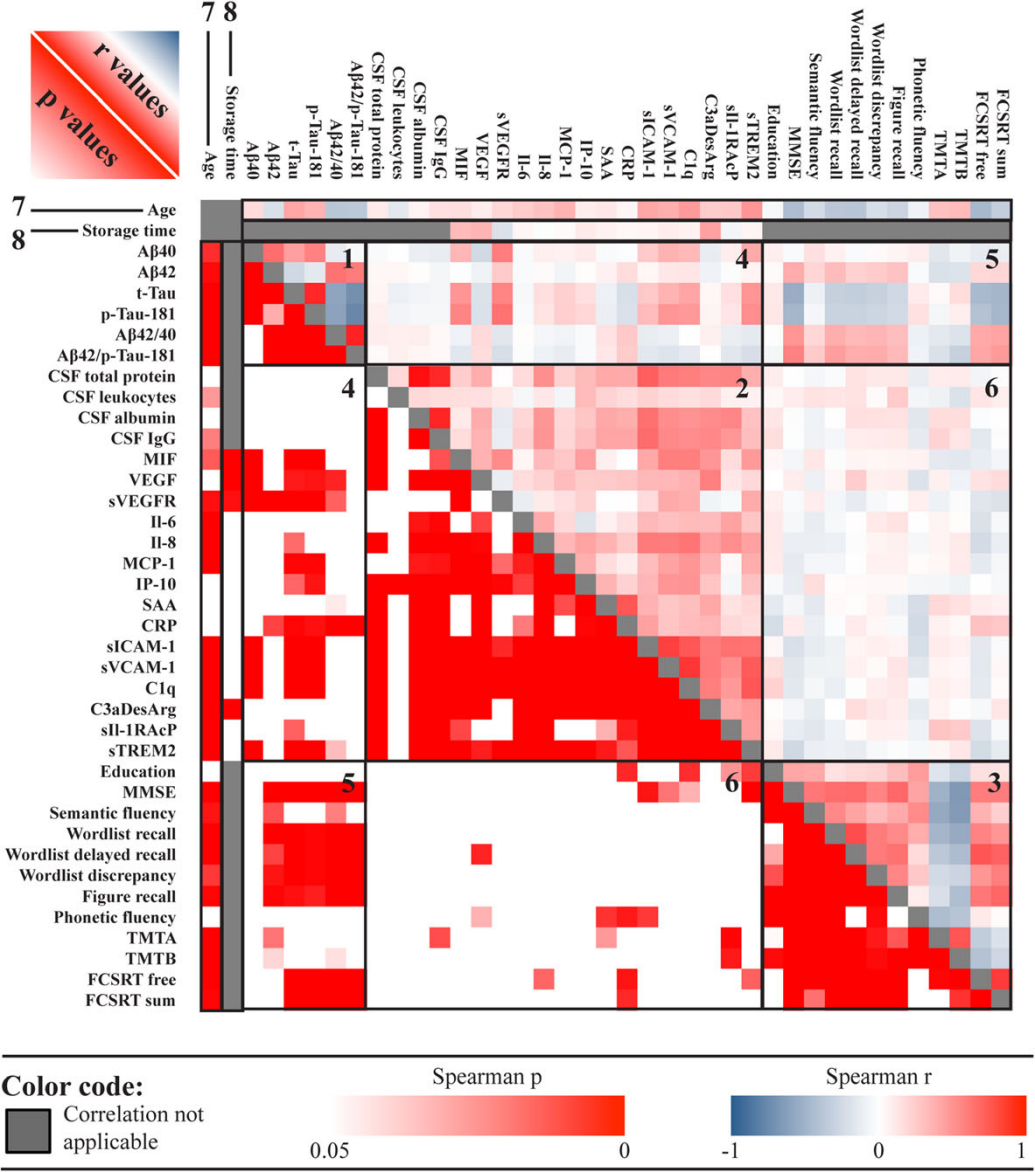
Correlation matrix of the main cohorts depicting results of nonparametric Spearman’s correlations, visualized by plotting significance values (*p*, *lower left*) against strength of correlation (*r*, *upper right*) as a heat map. In general, there were a large number of highly significant correlations of weak or moderate strength: (1) correlation of standard Alzheimer’s disease (AD) markers with each other, (2) cerebrospinal fluid (CSF) total protein and inflammatory markers against each other, (3) neuropsychiatric tests (NPTs) against each other, (4) AD markers against inflammatory markers, (5) AD markers against NPT results, (6) inflammatory markers against NPT results, and (7) and (8) influence of patient age and biobank storage time. *Aβ*_*40*_ β-Amyloid 1–40, *Aβ*_*42*_ β-Amyloid 1–42, *CRP* C-reactive protein, *FCSRT* Free and Cued Selective Reminding Test, *IgG* Immunoglobulin G, *IL* Interleukin, *IP-10* Interferon-γ-induced protein 10, *MCP-1* Monocyte chemoattractant protein 1, *MIF* Macrophage migration inhibitory factor, *MMSE* Mini Mental State Examination, *p-tau-181* Tau phosphorylated at position 181, *SAA* Serum amyloid A protein, *sICAM-1* Soluble intercellular adhesion molecule 1, *sIL-1RAcP* Soluble inhibition of soluble interleukin-1 receptor accessory protein, *sTREM2* Soluble triggering receptor expressed on myeloid cells 2, *sVCAM-1* Soluble vascular cell adhesion molecule 1, *TMT* Trail Making Test, *t-tau* Total tau, *VEGF* Vascular endothelial growth factor, *VEGFR* Vascular endothelial growth factor receptor

To analyze the potential associations among inflammatory markers and other features, we calculated a correlation matrix (Fig. 3). Inflammatory proteins were frequently correlated with other inflammatory, AD-specific, or abundant CSF proteins, although results differed strongly among the respective markers. Nearly all features of the main cohorts, including most inflammatory proteins, were significantly correlated with age (*see* Fig. 3, box 7). Moreover, many inflammatory markers were slightly elevated in male patients compared with female patients (*see* Additional file 2). Of the 15 tested markers, 4 were significantly correlated with biobank storage time. Unexpectedly, there were positive correlations between the proteins MIF, VEGF, and C3aDesArg with storage time (higher marker levels correlated with longer storage time; Fig. 3, box 8). For sVEGFR, the correlation with storage time was negative (longer storage correlating with lower values). The remaining markers did not show significant correlations with storage time.

**Fig. 4:**
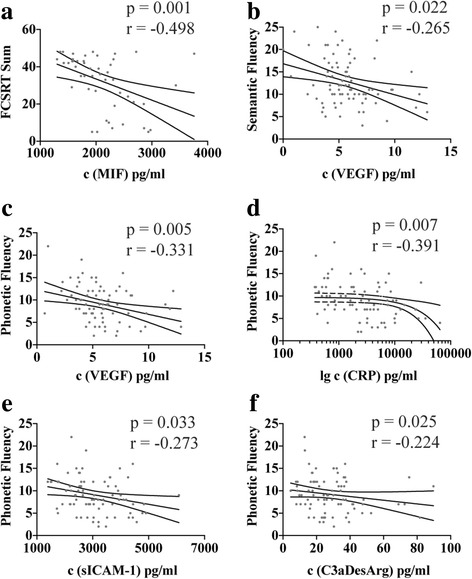
Association between inflammatory markers and cognitive performance in Alzheimer’s disease (AD). Six significant correlations were robust in multivariable linear regression analysis adjusting for age, sex, and education (*see* Additional file 2). Correlations (**a**) between the total sum of the Free and Cued Selective Reminding Test (FCSRT sum) and macrophage migration inhibitory factor (MIF), (**b**) between semantic fluency and vascular endothelial growth factor (VEGF), and between (**c**) phonetic fluency and VEGF, (**d**) C-reactive protein (CRP), (**e**) soluble intercellular adhesion molecule 1 (sICAM-1), and (**f**) C3aDesArg. Phonetic fluency was the most prominent cognitive correlate. In all cases, higher levels of inflammatory markers in the cerebrospinal fluid were associated with lower cognitive performance

Associations were highly consistent among AD biomarkers, other CSF proteins including the inflammatory markers, and NPTs, respectively (Fig. 3, boxes 1–3). However, several weak to moderate correlations (Spearman’s *r* < 0.3 or 0.6, respectively) were observed between NPT parameters and levels of inflammatory markers (Fig. 3, box 6). Six correlations in the AD cohort were robust in a multiple linear regression analysis adjusting for age, sex, and education level as covariates (Fig. 4 and Additional file 2). Of note, these associations were all negative and prominently included phonetic fluency.

Because of the influence of age and sex, we also performed ANCOVA of all significant cohort-wise and pathology-based comparisons, including storage time and the number of ApoE4 alleles as possible confounding factors for CRP and sTREM2 (*see* Additional file 2 for complete analysis). The association between CRP level and positive CSF amyloid pathology was robust in models adjusting for age, sex, storage time, or the number of ApoE4 alleles (all *p* < 0.05). In contrast, the association of CRP with positive CSF tau pathology or clinical cohorts (MCI or AD) was no longer significant after controlling for ApoE status (adjusted *p =* 0.546 and 0.581, respectively). For sTREM2, the association with clinical cohorts and pathological CSF amyloid or tau levels remained significant after controlling for sex, storage time, or the number of ApoE4 alleles (adjusted *p* < 0.05). After controlling for age, however, only the association of sTREM2 with tau remained significant (adjusted *p* = 3 × 10E^−7^), whereas its associations with clinical cohorts or CSF amyloid levels were no longer significant (adjusted *p* = 0.270 and *p* = 0.730, respectively).

Among the tau-associated proteins, VEGF, sVEGFR-1, IP-10, sVCAM-1, MIF, C1q, and sTREM2 were robust against age and other covariates (*see* Additional file 2 for confounder-adjusted comparisons). In contrast, sICAM-1, sIL-1RAcP, and MCP-1 were not significant in tau pathology-based comparisons when adjusted for age.

### Inflammatory markers in other neurodegenerative diseases

**Fig. 5:**
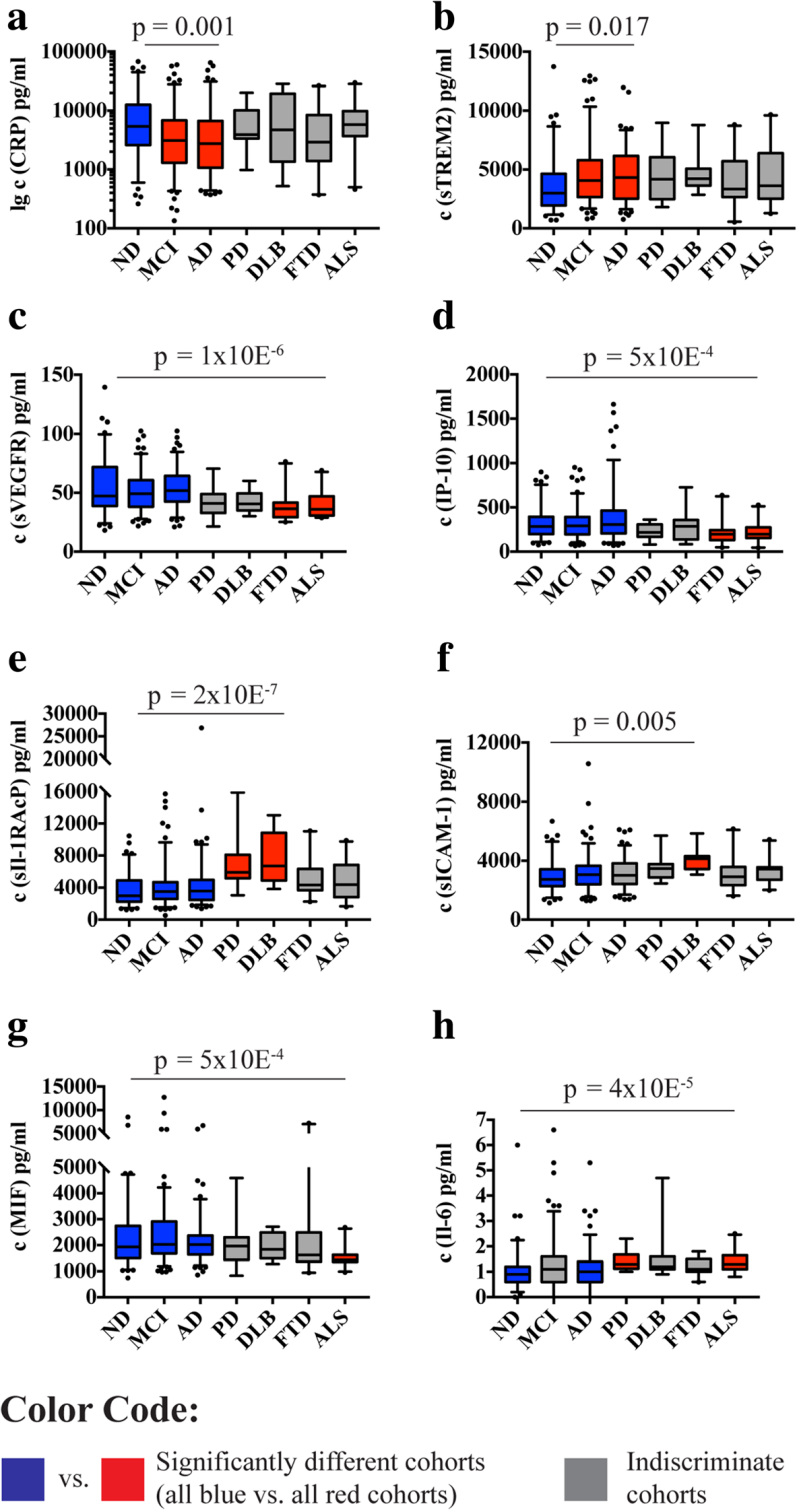
Inflammatory biomarkers in nondemented (ND) subjects and patients with mild cognitive impairment (MCI), Alzheimer’s disease (AD), and other neurodegenerative diseases. The figure shows results for inflammatory markers with significant differences in intercohort comparisons (*p* values derived by Kruskal-Wallis test; *see* Additional file 2 for all pairwise comparisons). Clustered cohorts with significant differences are colored *blue* and *red*; *gray* indicates indiscriminate cohorts. Effects observed for C-reactive protein (CRP) and soluble triggering receptor expressed on myeloid cells 2 (sTREM2) in the main cohorts remained significant after inclusion of supplemental cohorts in the Kruskal-Wallis test (**a**, **b**). Furthermore, significant differences between cohorts were observed for sVEGFR (**c**), IP-10 (**d**), sIl-1RAcP (**e**), sICAM-1 (**f**), MIF (**g**) and Il-6 (**h**). Box plots represent median and IQR, 5% and 95% percentile whiskers, and all data points outside that range. *ALS* Amyotrophic lateral sclerosis, *DLB* Dementia with Lewy bodies, *FTD* Frontotemporal dementia, *Ig* Immunoglobulin, *IL* Interleukin, *IP-10* Interferon-γ-induced protein 10, *MCI* Mild cognitive impairment, *MCP-1* Monocyte chemoattractant protein 1, *MIF* Macrophage migration inhibitory factor, *PD* Parkinson’s disease, *sICAM-1* Soluble intercellular adhesion molecule 1, *sIL-1RAcP* Soluble inhibition of soluble interleukin-1 receptor accessory protein, *sTREM2* Soluble triggering receptor expressed on myeloid cells 2, *sVEGFR* Soluble vascular endothelial growth factor receptor

When comparing the main cohorts with supplemental smaller cohorts of patients with PD, DLB, FTD, or ALS, the previously described effects for CRP and sTREM2 were found to be robust (Fig. 5 and Additional file 2). Several other inflammatory markers showed changes in at least one of the supplemental cohorts, usually when compared with the samples from the larger main cohorts. Noteworthy were the increased levels of sIL-1RAcP in both PD and DLB and the lower levels of sVEGFR and IP-10 in FTD and ALS, each compared with the nondemented, MCI, and AD cohorts.

### Discriminative power analysis

**Fig. 6:**
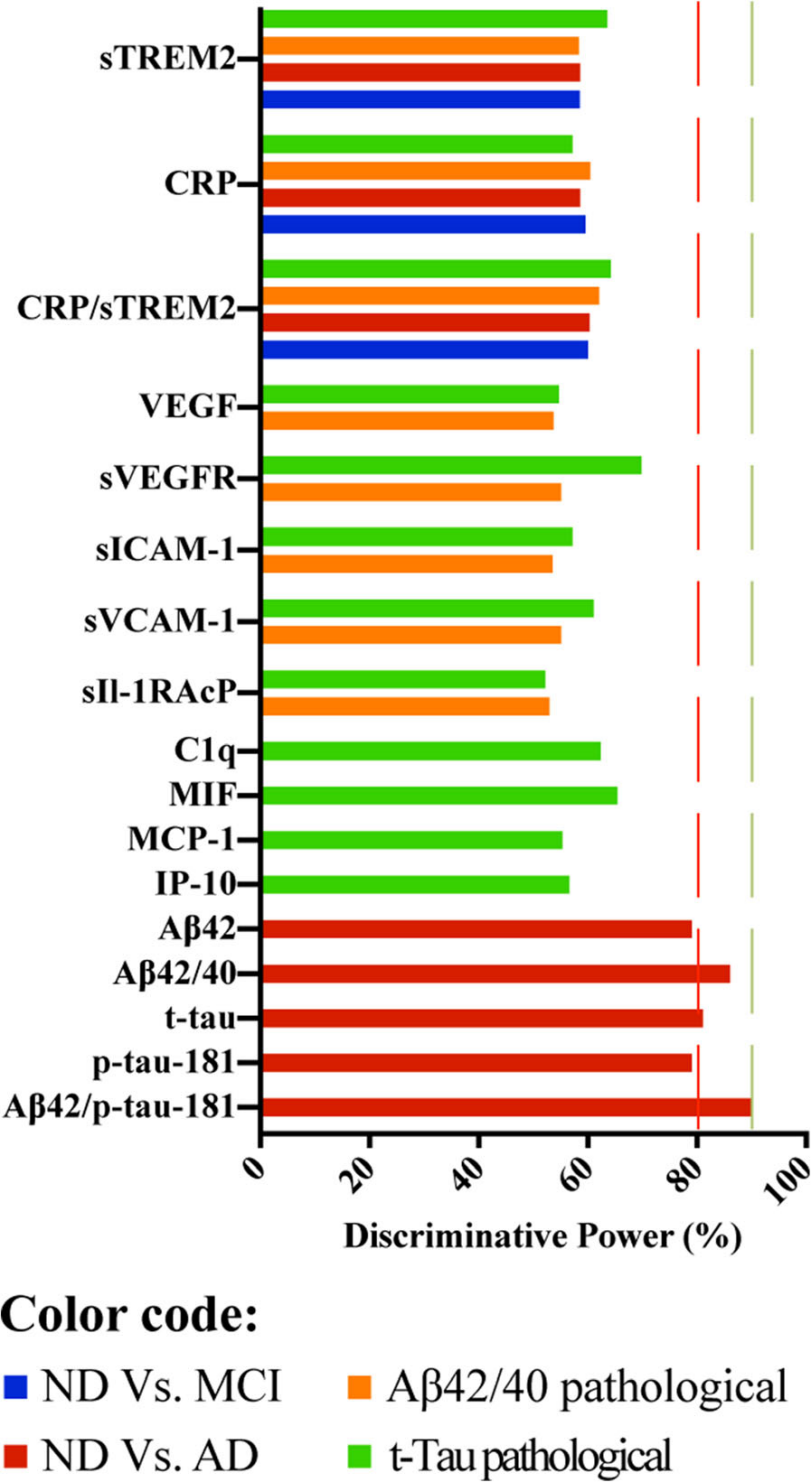
Diagnostic power of inflammatory biomarkers. ROC based on nonparametric data distribution to calculate cutoff values with equally weighted sensitivity and specificity (discriminative power). The figure shows the results for proteins associated with clinical cohorts or amyloid and tau levels based on the ratio Aβ_42_/Aβ_40_ and t-tau. In general, markers reached a discriminative power between 50% and 70%. For use as biomarkers, a power of at least 80–90% (indicated by *red* and *green dashed lines*) would be necessary. Values in that range are achieved using Alzheimer’s disease core biomarkers, which are shown for comparison purposes based on the internal laboratory-specific cutoff values. *Aβ*_*40*_ β-Amyloid 1–40, *Aβ*_*42*_ β-Amyloid 1–42, *AD* Alzheimer’s disease, *ALS* Amyotrophic lateral sclerosis, *CRP* C-reactive protein, *IP-10* Interferon-γ-induced protein 10, *MCI* Mild cognitive impairment, *MCP-1* Monocyte chemoattractant protein 1, *MIF* Macrophage migration inhibitory factor, *ND* Nondemented, *p-tau-181* Tau phosphorylated at position 181, *sICAM-1* Soluble intercellular adhesion molecule 1, *sIL-1RAcP* Soluble inhibition of soluble interleukin-1 receptor accessory protein, *sTREM2* Soluble triggering receptor expressed on myeloid cells 2, *sVCAM-1* Soluble vascular cell adhesion molecule 1, *sVEGFR* Soluble vascular endothelial growth factor receptor, *t-tau* Total tau, *VEGF* Vascular endothelial growth factor

To be relevant in the clinical context, biomarkers should reach sensitivities and specificities of 90% or more [20]. For this reason, we assessed how the inflammatory markers would perform if cutoff values were used to distinguish cohorts defined either by clinical diagnosis or by pathological CSF amyloid or tau levels (Fig. 6 and Additional file 2). If sensitivity and specificity were weighted equally (summarized by the term discriminative power), an average power of 50–70% was attained, depending on the marker and type of comparison (*see* Additional file 2). The weakest discriminator was sIL-1RAcP for tau pathology (discriminative power of 52.2%), and the strongest was sVEGFR for tau pathology (discriminative power of 69.8%). CRP and sTREM2 reached a power of 58.6–59.5% when discriminating nondemented subjects vs. patients with MCI or nondemented subjects vs. patients with AD. Because the CRP and sTREM2 proteins showed changes in opposite directions (lower levels for CRP and higher ones for sTREM2), we explored whether a ratio between both protein levels could improve power. Of different tested variants including log-normalized CRP values, only the CRP/sTREM2 ratio was discriminative (Kruskal-Wallis *p*, pairwise *p* nondemented vs. MCI and nondemented vs. AD all *p* < 0.05; *see* Additional file 2), but this only marginally improved power (60.0–60.3%). For comparison purposes, Fig. 6 also shows the power of the standard AD markers for the internal laboratory-specific cutoff values.

## Discussion

Briefly, this study had three main objectives: (1) to ensure robust detectability of all included inflammatory biomarkers in the CSF, (2) to investigate potential associations between those inflammatory markers and important pathological features of AD, and (3) to test whether any inflammatory protein markers would achieve sufficient discriminative power to aid in clinical diagnostic procedures. Studies on CSF inflammation-associated markers in AD have provided quite heterogeneous results, and no powerful marker has yet gained salience for use in clinical practice [5]. Differences in cohort composition, sample handling, and choice of assays have a major impact on the results obtained in such studies [21]. Aside from this, low CSF concentrations of inflammatory mediators constitute another important limitation. Several recent studies have highlighted that many proteins included in typical multiplex panels are below detection limits when investigating AD CSF or brain tissue samples [7, 8]. This is consistent with the initial testing phase in the present study, where roughly one-third of tested proteins, especially very frequently reported pro- and anti-inflammatory cytokines, were found to be below detection limits. The number of detectable cytokines and chemokines was limited to a few analytes such as IL-8, MCP-1, or IP-10. In contrast, robustly detectable markers were proteins from different immune signaling pathways or associated with multiple biological processes, such as soluble receptors, complement factors, or vascular signaling. These findings indicate that data on inflammatory markers with low abundance should be treated with caution. Furthermore, future research should either be focused on analysis of pathways of robustly detectable markers or use more sensitive assay systems to overcome such analytical limitations.

Another technical finding of this study was related to biobank storage time. Storage time was correlated with CSF levels of VEGF, sVEGFR, MIF, and C3aDesArg. In general, it would be expected that samples would lose quality over time and that measured levels would decrease as analytes deteriorate. However, this was observed only for sVEGFR. Statistically, for the other three markers, there was a perplexing positive correlation between storage time and levels. Whether this observation was a data artefact or due to actual biochemical or biophysical processes, such as separation from binding proteins or changes to epitopes recognized by antibodies, could not be clarified with the available dataset. It is noteworthy that storage time was not a critical covariate for the majority of clinical findings in this study.

Among the panel of detectable markers, two proteins stood out when we compared nondemented, MCI, and AD samples: CRP and sTREM2. CRP is one of the most common peripheral blood biomarkers for cardiovascular and inflammatory disorders. Similarly to several other inflammatory markers, elevated peripheral blood levels of CRP have frequently been associated with increased risk of dementias and cognitive decline in multiple studies, although less robustly with AD itself [22, 23]. It is noteworthy that CRP levels showed a lognormal distribution in CSF, similar to those found in peripheral blood, which hampers interpretation of CRP levels in general [24]. However, median levels of CRP decreased among groups, from nondemented to MCI to AD samples. This is consistent with findings reported by Schuitemaker et al*.*, who described lower CSF CRP levels in patients with AD than in those with MCI [25]. Our study yielded no significant findings for CRP in PD samples, although others have described higher CSF CRP values in patients with PD with dementia than in patients with PD and control patients [26]. Furthermore, CRP levels were robustly associated with CSF amyloid levels and *ApoE* genotype, but not with tau or the clinical cohorts. These results are well in accordance with studies of CRP function in AD. CRP gene variants have been associated with plaque development in AD [27]. The protein has been linked to amyloid pathology in APP/PS1 (amyloid precursor protein/presenilin 1) mice, and Strang et al*.* have described dissociation of pentameric into monomeric CRP induced by amyloid plaques [28, 29]. Monomeric CRP has been discussed as a linker between vascular damage and inflammation on the one hand and plaque formation, neuronal damage, and dementia risk on the other [30]. Regarding the association of CRP with the *ApoE* genotype, several reports have described statistical interactions between peripheral CRP levels and *ApoE* genotype. For example, Hubacek et al*.* described lower plasma CRP levels in *ApoE4* carriers than in *ApoE3* carriers [31]. It should be noted that CRP is produced primarily in the liver, and local production of CRP in the CNS does not appear to contribute significantly to CSF levels [32]. Hence, on the one hand, it could be hypothesized that CSF levels of CRP in AD are a consequence of decreased blood levels associated with the *ApoE4* genotype, which is simultaneously the strongest genetic risk factor for AD. On the other hand, the negative association found with phonetic fluency in patients with AD is consistent with the fact mentioned above that elevated peripheral CRP levels provide a risk factor for dementia [22, 23]. Thus, CRP functions as well as CRP levels are likely linked by multiple mechanisms to AD pathology.

The other significant marker, sTREM2, has received tremendous attention since the discovery of *TREM2* gene variants as risk factors for AD. Its functional role in AD and other neurodegenerative diseases, although not without controversy, has been thoroughly investigated and reviewed [12, 33, 34]. In the present study, sTREM2 was found to be elevated in MCI and AD cohorts and to be strongly associated with age and tau pathology, but with neither *ApoE* genotype nor amyloid levels. These findings are highly consistent with previous reports [35–39]. In part, this redundancy is probably due to the use of the same assay (the protocol described by Suárez-Calvet et al*.*). Differences between results were of a minor nature, such as those related to the influence of sex on sTREM2 CSF levels.

Given the oppositional regulation of CRP and sTREM2 levels in the CSF, we tested how a ratio between levels of both proteins would impact significance and power. Although this ratio differed significantly between the nondemented and MCI as well as AD cohorts, discriminative power was only slightly improved, resulting in no added value of the ratio compared with its components. An explanation might be that in contrast to the Aβ_42_/Aβ_40_ ratio, which is made of mechanistically linked components, CRP and sTREM2 do not share a common pathway, aside from being inflammation-associated in general. So far, only in African American women has a *TREM2* variant been linked to higher peripheral CRP levels [40]. Hence, CRP and sTREM2 are probably involved in different processes and stages of AD pathology, which could be the reason why a ratio does not improve cohort discrimination.

A range of further markers such as VEGF, sVEGFR-1, IP-10, sVCAM-1, MIF, and C1q, were robustly associated with pathological tau levels, independent of patient age. Basically, tau-associated proteins could be clustered into three groups when using age as a covariate: (1) proteins for which age was not a significant covariate at all; (2) markers for which there was an influence of age, whereas the association with tau was still significant; and (3) markers that were apparently entirely age-dependent and no longer significant for tau after adjusting for age. Hence, the influence of aging on CSF marker levels differs from molecule to molecule. Because CSF tau is considered a marker of pathological processes in later stages of AD, such as neuronal death, it is possible that the levels of these markers increase in the CSF owing to inflammatory signaling in response to tissue damage. This would be consistent with the associations found between inflammatory markers and cognitive outcomes, primarily phonetic fluency in AD, which is associated with frontal and temporal lobe integrity. Thus, CSF levels of these markers correlate to some extent with the pathological processes in the brain only in late stages of the disease and with cognitively demanding tests. Importantly, this does not exclude involvement of the immune system and inflammation within the brain in earlier stages of AD. It does, however, indicate that traceability of earlier inflammatory processes in the CSF might be limited owing to lack of detectability and effect strength of respective markers.

Comparison with other neurodegenerative diseases was limited by sample size in this study. Still, there were several striking findings. First, the clustered effects observed for CRP and sTREM2 in the main cohorts were robust in the supplemented cohorts. Second, sIL-1RAcP was elevated in both PD and dementia with DLB, which are both considered synucleinopathies [41]. Last, sVEGFR and IP-10 differed between the main cohorts and FTD as well as ALS, which are also speculated to belong to one disease spectrum, in particular since the discovery of the *C9orf72* mutations [42]. These results could indicate potential targets for research on inflammatory biomarkers in the respective disorders, but they require validation in larger cohorts.

When discussing biomarker findings, it is of great importance to consider not only statistical significance but also the effect strength, sensitivity, and specificity of the biomarker candidates [9, 43]. This was the third and most important aim of this study. Sensitivity and specificity were weighted equally and summarized by the term discriminative power. Despite a large number of significant associations between clinical cohorts or AD pathology markers on the one hand and inflammatory markers on the other, none of the tested scenarios reached a discriminative power high enough for applicability in clinical diagnostic procedures. Overall, inflammatory markers were clearly associated with various pathological features of AD but did not show changes in CSF levels to the extent of established AD amyloid and tau markers. In consequence, the inflammatory processes involved in AD pathogenesis are not reflected in CSF in the same way as amyloidosis or tauopathy. Implementation of assays with higher sensitivity or investigation of signaling mediators from alternative pathways could lead to discovery of candidates with higher potential for use in clinical diagnostic procedures.

## Conclusions

The robustness of detection of inflammatory markers in CSF is an important analytical limitation for biomarker studies. A large range of associations between inflammatory biomarkers and AD pathology could be observed. However, none of the inflammatory markers reached sufficient sensitivity or specificity to enable application in clinical practice. In future studies, implementation of assays with higher sensitivity or investigation of signaling mediators from alternative pathways could provide candidates with higher potential for actual use in diagnostic procedures [44–46].

## Additional files

Additional file 1: Technical supplement: initial test phase results. (PDF 3888 kb)

Additional file 2: Statistical supplement. (PDF 781 kb)

## Abbreviations

Aβ_40_: β-Amyloid 1–40
Aβ_42_: β-Amyloid 1–42
AD: Alzheimer’s disease
ALS: Amyotrophic lateral sclerosis
ANCOVA: Analysis of covariance
ApoE: Apolipoprotein E
ApoE4: Apolipoprotein E ε4 allele
CNS: Central nervous system
CRP: C-reactive protein
CSF: Cerebrospinal fluid
CV: Coefficient of variation
DLB: Dementia with Lewy bodies
FCSRT: Free and Cued Selective Reminding Test
FTD: Frontotemporal dementia
IgG: Immunoglobulin G
IL: Interleukin
IP-10: Interferon-γ-induced protein 10
MCI: Mild cognitive impairment
MCP-1: Monocyte chemoattractant protein 1
MIF: Macrophage migration inhibitory factor
MMSE: Mini Mental State Examination
ND: Nondemented
NPT: Neuropsychological test
PCA: Principal component analysis
PD: Parkinson’s disease
p-tau-181: Tau phosphorylated at position 181
SAA: Serum amyloid A protein
sICAM-1: Soluble intercellular adhesion molecule 1
sIL-1RAcP: Soluble inhibition of soluble interleukin-1 receptor accessory protein
sTREM2: Soluble triggering receptor expressed on myeloid cells 2
sVCAM-1: Soluble vascular cell adhesion molecule 1
TMT: Trail Making Test
t-tau: Total tau
VEGF: Vascular endothelial growth factor
VEGFR: Vascular endothelial growth factor receptor
