# Supplementary material for: Characterization and clinical use of inflammatory cerebrospinal fluid protein markers in Alzheimer’s disease
Source: Alzheimers Res Ther. 2018 Feb 26;10:25. doi: 10.1186/s13195-018-0353-3 (PMC5828084; doi:10.1186/s13195-018-0353-3)

Technical supplement: Initial test phase results

Table of Contents: **Page**

Table of Contents:	Page
**1. Methods**	1
i. List of assays	1
ii. Testing procedures	2
iii. Evaluation criteria	2
**2. Results**	3
i. Classification of assays	3
ii. Grouping of assays	4
iii. Explanatory results (example with figure legends	5
iv. Single analytes results	6 - 54

1. Methods:

i. List of assays

- V-PLEX Human Biomarker 40-Plex Kit, K15209D, Mesoscale Diagnostics LLC
  - Consists of 5 different multiplex kits:
  - V-PLEX Chemokine Panel 1 (human) Kit, K15047D: Eotaxin, Eotaxin-3, IL-8, IL-8 (HA), IP-10, MCP-1, MCP-4, MDC, MIP-1α, MIP-1β, TARC
  - V-PLEX Proinflammatory Panel 1 (human), K15049D: IFN-γ, IL-10, IL-12p70, IL-13, IL-1β, IL-2, IL-4, IL-6, IL-8, TNF-α
  - V-PLEX Cytokine Panel 1 (human), K15050D: GM-CSF, IL-12/IL-23p40, IL-15, IL-16, IL-17A, IL-1α, IL-5, IL-7, TNF-β, VEGF-A
  - V-PLEX Angiogenesis Panel 1 (human), K15190D: FGF (basic), Flt-1/VEGFR-1, PlGF, Tie-2, VEGF-A, VEGF-C, VEGF-D
  - V-PLEX Vascular Injury Panel 2 (human), K15198D: CRP, ICAM-1, SAA, VCAM-1
- Human MIF Kit, K151LVD, Mesoscale Diagnostics LLC
- Human Complement C3a des Arg ELISA Kit, ab133037, Abcam plc
- Human Complement C1q ELISA kit, ab170246, Abcam plc
- Human Complement Component C5a DuoSet® ELISA, DY2037, R&D Systems Inc.
- Human IL-1 RAcP/IL-1 R3 DuoSet® ELISA, DY676, R&D Systems Inc.
- Human soluble TREM2 was analyzed using the protocol established by Suárez-Calvet et. al., “sTREM2 cerebrospinal fluid levels are a potential biomarker for microglia activity in early-stage Alzheimer's disease and associate with neuronal injury markers.” EMBO Mol Med 2016;8:466-476.
- Human Mrp8/14 heterodimer was analyzed by the group of Prof. Dr. Thomas Vogl, Institut für Immunologie, Medizinische Fakultät, Westfälische Wilhelms-Universität Münster, Germany.

ii. Testing procedures

1. Regular standard curve for comparison purposes
2. Spike-in of standard curve in a CSF/diluent mixture
   - The spike in was designed in a way that the concentrations of the standard protein would be the same as for the regular standard curve. In example, if a regular standard would be a 8-point curve ranging from 2000 to 30 pg/ml, the spike-in would also be a 8-point curve ranging from 2000 to 30 pg/ml.
   - In contrast to the regular standard, the spike-in curve was prepared not in regular assay diluent, but in a mixture of CSF with the assay diluent. Hence, readouts of the spike-in curve would be modified by CSF derived, endogenous protein. The CSF/Diluent mixture was usually done in a ratio of 1:1, except for those assays were vendors gave specific instructions on how to dilute the CSF (e.g., for C1q a dilution of 1:300 was provided by the vendor and applied in the spike-in).
   - The concept was that this procedure would allow to test for matrix effects derived from CSF. More importantly, if there are endogenous levels of a certain protein in CSF, the signal of the spiked-in curve should be increased over that of the regular standard curve.
   - Furthermore, as each spike-in was prepared as a 8-point-curve, there would be 8 data points available to calculate CSF endogenous levels by subtracting the known spiked-in standard concentrations. Theoretically, this should provide the same result for all data points of the spiked-in standard curve.
3. Dilution series of a pooled CSF sample to determine dilution linearity and optimal dilution range.
4. In a second experiment, a small set of ND, MCI and AD test samples (n = 6) was applied to the assays to obtain some basic information on the distribution of values obtained from actual research samples. Any significant difference found between test samples would be valued as a positive feature of the assay.

iii. Evaluation criteria

- Evaluation classified assays as follows:
- Passed: Assay was fully functional, samples were detectable within the quantitative range and had low technical variance
- Not passed: Proteins were undetectable with the assay used due to concentrations below quantitative or detection range, high variance, and failed spike-in-experiments
- Borderline: Sample concentrations were at borderline or undetectable, but spike-in tests suggested that it might be possible to calculate concentrations if a known dose of protein would be added to samples. In concept, this spiked-in protein would mix with the CSF protein to elevate signal levels into the quantitation range. After measurements, CSF concentrations would be calculated by subtracting the known spiked-in dose from the result. A prerequisite was that the concentrations calculated from the spiked-in standard curve in the test experiment were the same over a range of at least 4 different spiked in concentrations.

2. Results:

i. Classification of assays

Passed	Borderline	Not passed
C1q	Flt-1/VEGFR1	C5a
C3a des Arg	Il-5	bFGF
CRP	Il-6	Eoataxin
ICAM-1	Il-7	Eotaxin-3
Il-1 RAP/Il-1 R3	Il-12B/Il-12p40	GM-CSF
Il-8/CXCL8	Il-15	IFN-γ
IP-10/CXCL10	Il-16	Il-1α
MCP-1/CCL2	MDC/CCL22	Il-1β
MIF	TARC/CCL17	Il-2
MIP-1β/CCL4	VEGF	Il-4
PIGF		Il-10
SAA		Il-12p70
TREM2		Il-13
VCAM-1		Il-17A
		MCP-4
		MIP-1α
		Mrp8/14
		Tie-2
		TNF-α
		TNF-β
		VEGF-C
		VEGF-D

ii. Grouping of assays

The table lists the protein assays selected based on the initial test phase together with the respective analytical properties. Assays were run as multiplexes (M) or singleplexes (S) depending on the specific diluents, dilution factors and detection ranges, which differed significantly between the target proteins. The overall aim of the setup was to obtain sample signals above lower limit of detection (LLOD) or – preferably – above lower limit of quantitation (LLOQ) and well within the assays range. Proteins at borderline of detection (indicated by asterisk *) were run in spike-in-setups to ensure that signals of low-abundant proteins are within detection range.

**Protein**	**Setup**	**LLOD (pg/ml)**	**LLOQ (pg/ml)**	**Standard range (pg/ml)**	**Dilution (x-fold)**	**Spike-In (pg/ml)**	**Mean CV (%)**
**VEGF***	M	0.48	5.0	0.48 - 1970	2	7.38	4.8
**sVEGFR-1/Flt-1***		0.56	10.0	1.99 – 8170		35.2	3.6
**MCP-1/CCL2**	M	0.09	1.09	0.11 – 448.0	10	No	4,4
**IP-10/CXCL10**		0.37	1.37	0.59 - 2400			3.0
**Il-6***	M	0.06	1.58	0.18 - 745	2	0.76	2.8
**Il-8/CXCL8**		0.04	1.13	0.16 – 666		2.57	2.1
**SAA**	M	10.9	54.1	14.0 - 218x10E^3	10	No	5.2
**CRP**		1.33	27.6	12.0 - 195x10E^3			1.8
**sICAM-1/CD54**		1.03	6.4	4.0 – 61.4x10E^3			3.9
**sVCAM-1/CD106**		6.9	37.6	3.0 – 51.7x10E^3			2.2
**MIF**	S	2.4	N.A.	3.7 – 15.0x10E^3	5	No	2.5
**C1q**	S	30.0	N.A.	10.0 – 40.0x10E^3	300	No	3.8
**C3aDesArg**	S	N.A.	N.A.	31.3 – 20.0x10E^3	10 - 50	No	5.7
**sIl-1RAcP**	S	N.A.	N.A.	31.3 - 2000	25 - 50	No	3.5
**sTREM2**	S	N.A.	N.A.	8x10^3 – 200x10E^3	4	No	1.5

iii. Explanatory results (example with figure legends)

We evaluated each assay to estimate if it would provide reliable data in CSF analysis. Evaluation included the assays overall performance, variance of duplicate measurements, spike-in tests, a CSF dilution series and a limited number of test research samples. This page serves as an explanatory example for the results that are provided for each single protein on page 7-54.

MIF (Singleplex) — Protein name and assay setup

Evaluation (classification): Passed

Optimal dilution factor (estimated from dilution series) is 5x

- Spike-In-Curve (red) has positive difference to standard curve (blue)
- Spike in Concentrations are linear in range of standard 4 – 8 at 1000 pg/ml (2x Dilution)
- Sample concentrations > LLOD (here: 2.4 pg/ml)
- Sample concentrations in linear range
- All QC parameters (detectability of samples, variance of coefficient CV < 20%) passed
- No significant difference between test samples

A Summary of test phase results and the classification of the assay as “passed”, “borderline” or “not passed”.

Signal of regular standard curve (**blue**), the standard curve spiked into the CSF/diluent mixture (**red**) and the CSF sample dilution series (**black**), plotted against the concentration of the standard protein

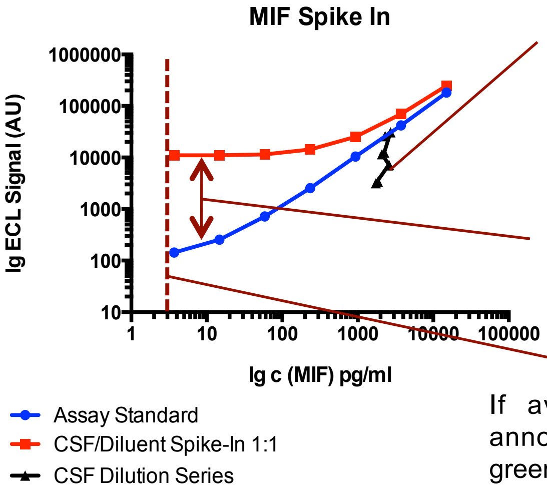

Signal from diluted CSF samples (black) is ideally above LLOD/LLOQ and in linear assay range

Endogenous CSF protein increases spike-in signal (red) above that of regular assay standard (blue)

If available, red lines annotate assay LLOD; green lines assay LLOQ

Protein concentrations recalculated from the standard spiked into CSF/diluent mixture (y-axis) plotted against the concentration of the spiked-in standard (x-axis). Ideally, recalculated concentrations should be identical, positive values.

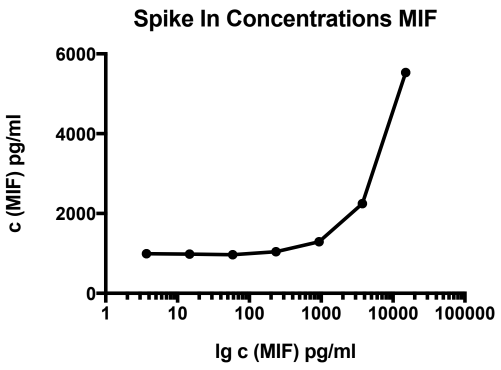

Protein concentrations recalculated from the CSF dilution series, plotted against used dilution factors. Ideally, calculated concentrations should be identical for all dilutions

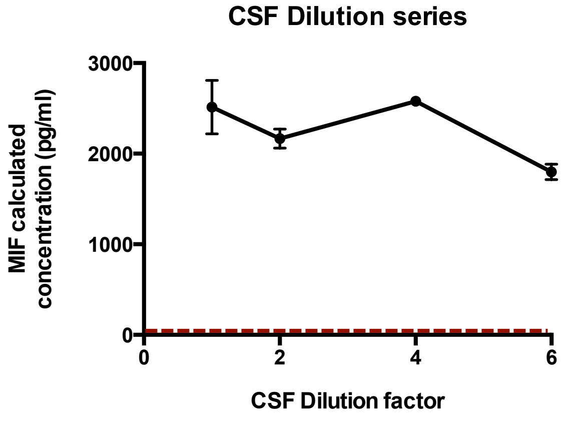

Data from the n = 6 test sample cohort, primarily used to estimate inter-individual variances and ensure research samples are in detection range

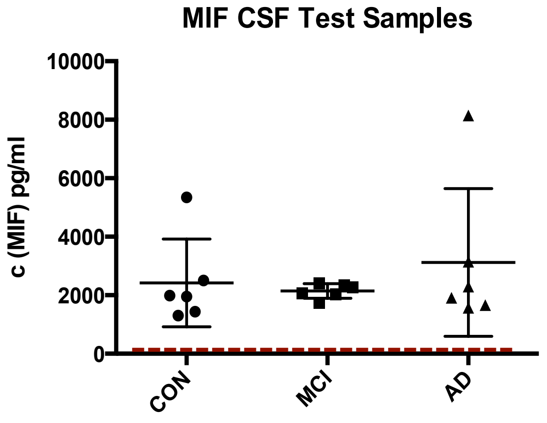

iv. Table of contents: Single analytes results

Analyte	Page	Analyte	Page
C1q	7	Il-13	31
C3a des Arg	8	Il-15	32
C5a	9	Il-16	33
CRP	10	Il-17A	34
bFGF	11	IP-10	35
Eotaxin	12	MCP-1	36
Eotaxin-3	13	MCP-4	37
Flt-1	14	MDC	38
GM-CSF	15	MIF	39
ICAM-1	16	MIP-1α	40
IFN-γ	17	MIP-1β	41
Il-1α	18	Mrp8/14	42
Il-1β	19	PIGF	43
Il-1RAcP	20	SAA	44
Il-2	21	TARC	45
Il-4	22	Tie-2	46
Il-5	23	TNF-α	47
Il-6	24	TNF-β	48
Il-7	25	TREM2	49
Il-8 (Chemokine Multiplex)	26	VCAM-1	50
Il-8 (Proinflammatory Panel Multiplex)	27	VEGF (Cytokine Panel Multiplex)	51
Il-10	28	VEGF (Angiogenesis Panel Multiplex)	52
Il-12p40	29	VEGF-C	53
Il-12p70	30	VEGF-D	54

iv. Single analytes results

Complement C1q (colorimetric singleplex)

Evaluation: Passed

Optimal dilution factor is 300x (confirmed vendor specification)

- Spike-In-Curve has positive difference to standard curve
- Spike in Concentrations are linear in range of standard 3 – 8 at 1.0 ng/ml (300x Dilution)
- Sample concentrations > LLOD (0.03 ng/ml)
- Sample concentrations in linear range if diluted
- No significant difference between test samples

A) Spike In Curve

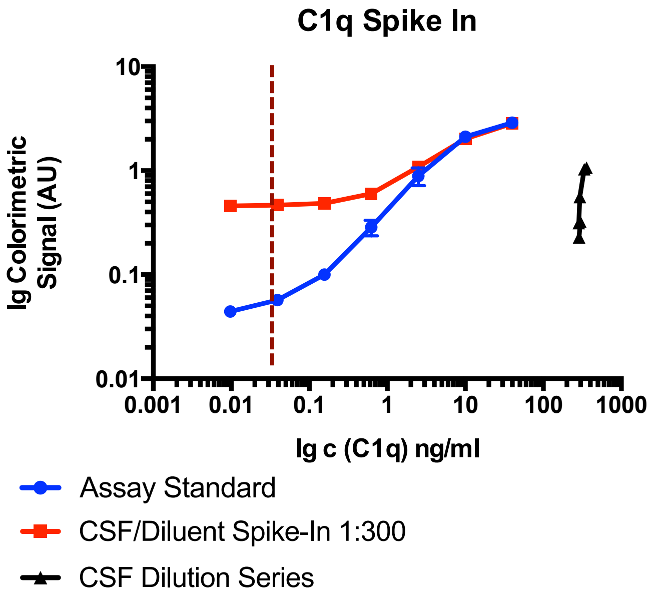

B) Recalculated concentrations curve

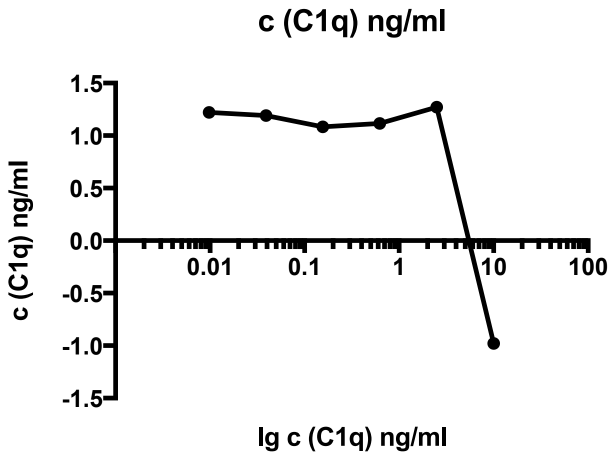

C) CSF dilution series

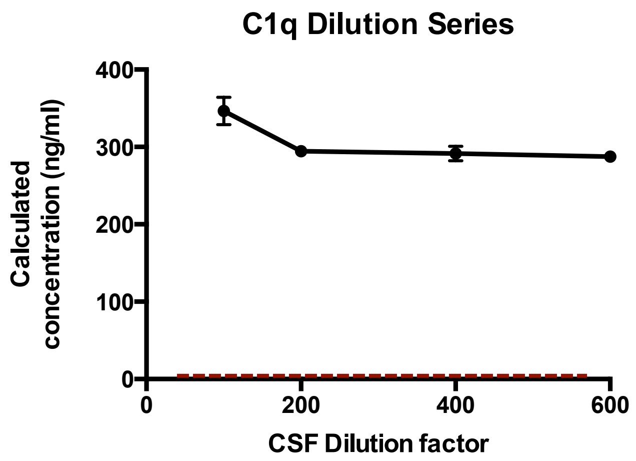

D) Test sample results

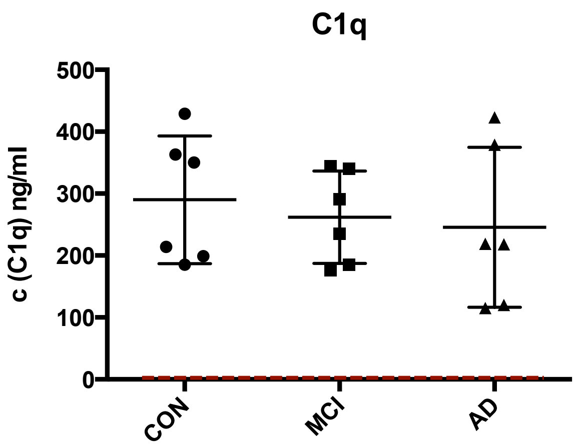

iv. Single analytes results

Complement C3a des Arg (competitive colorimetric singleplex)

Evaluation: Passed

Optimal dilution factor is 50x

- Spike-In-Curve has negative difference to standard curve (competitive assay; less signal equals higher concentrations)
- Sample concentrations in linear range if diluted
- One of the research test samples has CV > 20% while average CV is < 10%
- No significant difference between test samples

A) Spike In Curve

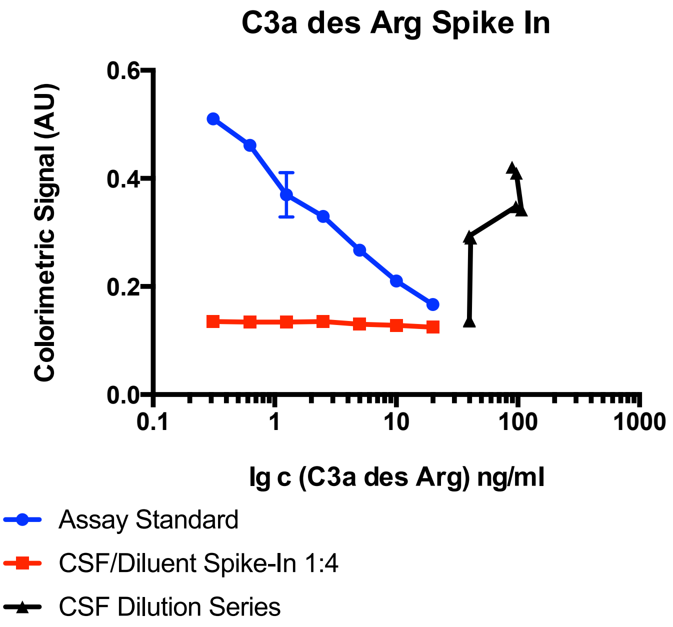

B) Recalculated concentrations curve

In the 1:4 CSF/diluent mixture used, C3a concentrations were above detection limits as this complement factor is highly abundant in CSF.

C) CSF dilution series

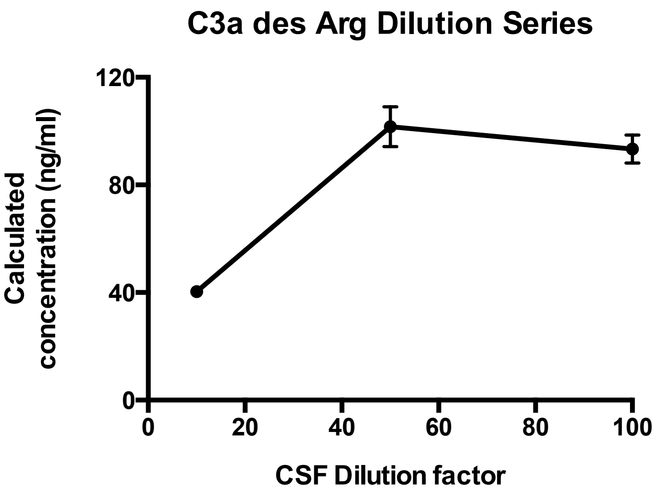

D) Test sample results

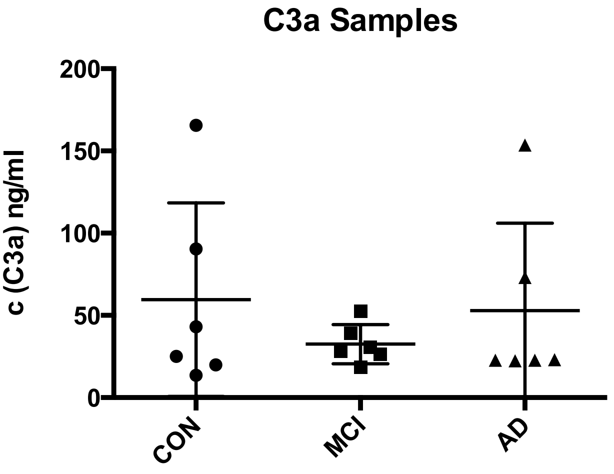

iv. Single analytes results

Complement C5a (colorimetric singleplex)

Evaluation: Not passed

Only few samples detectable, spike-in setup performed to unstable

- Spike-In-Curve overlaps with standard curve
- Spike-In concentrations are nonlinear
- Samples undetectable in dilution series
- Majority of samples fails QC parameters
- No significant difference between test samples

A) Spike In Curve

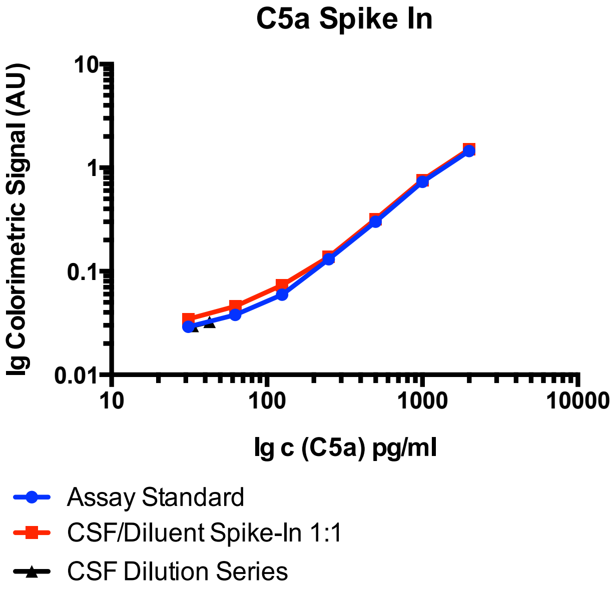

B) Recalculated concentrations curve

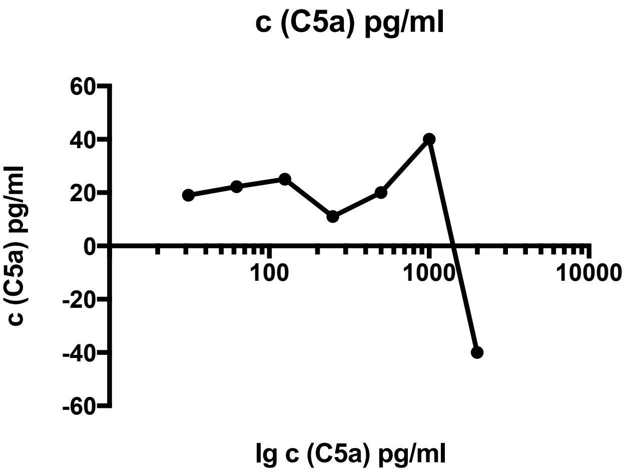

C) CSF dilution series

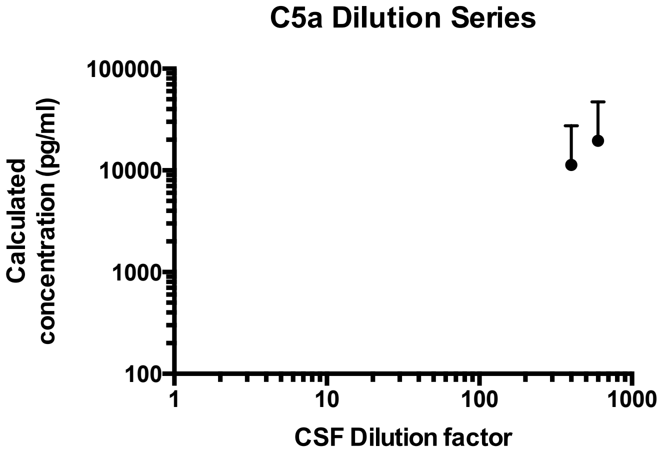

D) Test sample results

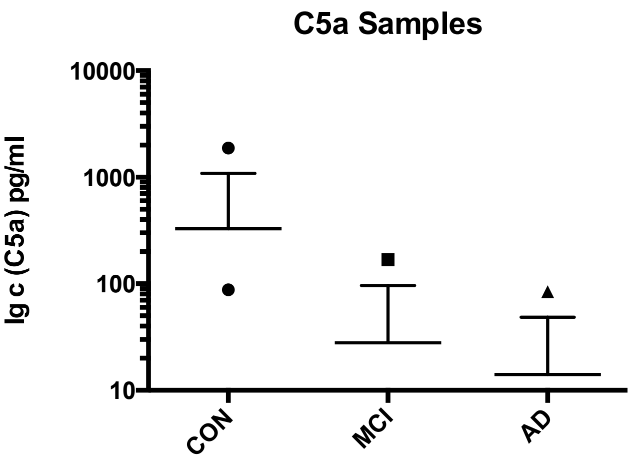

iv. Single analytes results

CRP (electrochemiluminescence multiplex; vascular injury panel)

Evaluation: Passed

Optimal dilution factor is >>6x

- Spike-In-Curve has positive difference to standard curve
- Spike in Concentrations are linear in range of standard 3 – 8 at 100.000 pg/ml (2x Dilution)
- Sample concentrations > LLOQ (27.6 pg/ml)
- Sample concentrations in linear range
- All QC parameters passed
- Tendency: AD < Control significant in nonparametric ANOVA

A) Spike In Curve

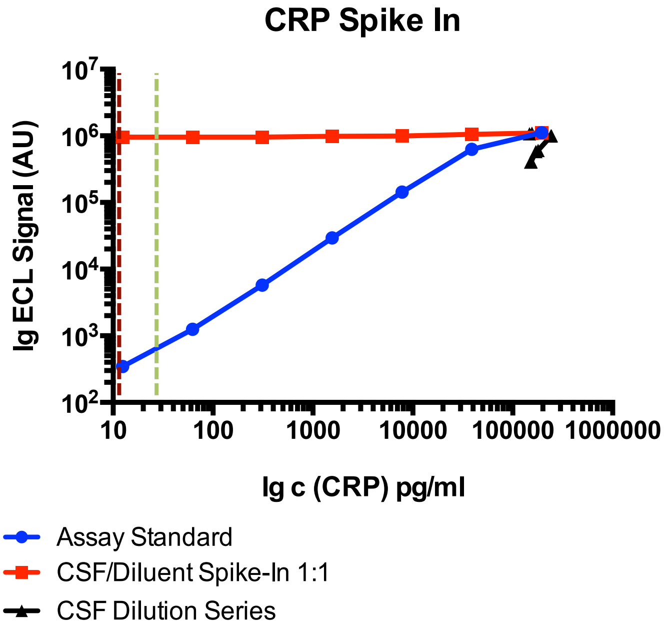

B) Recalculated concentrations curve

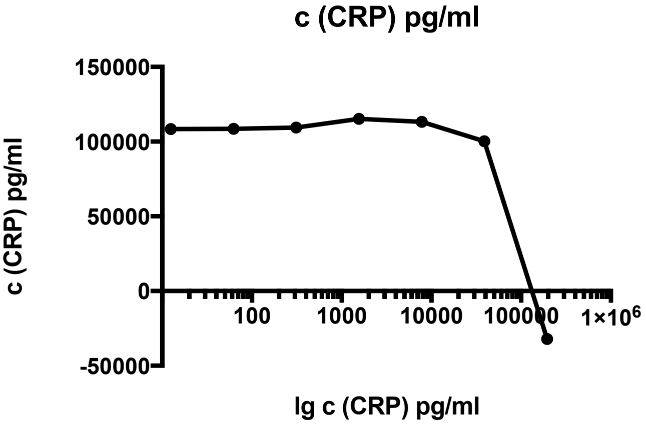

C) CSF dilution series

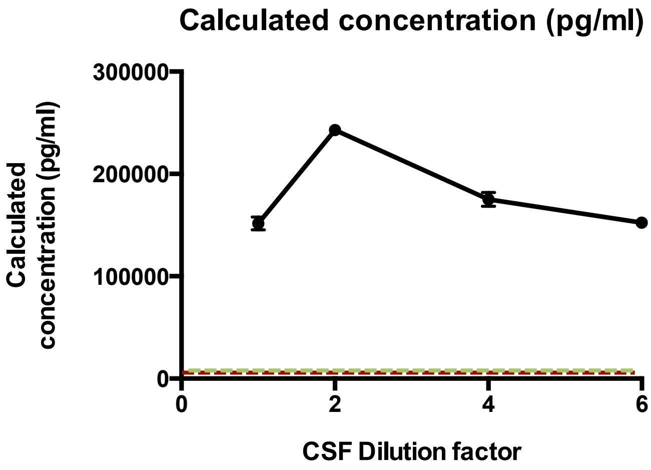

D) Test sample results

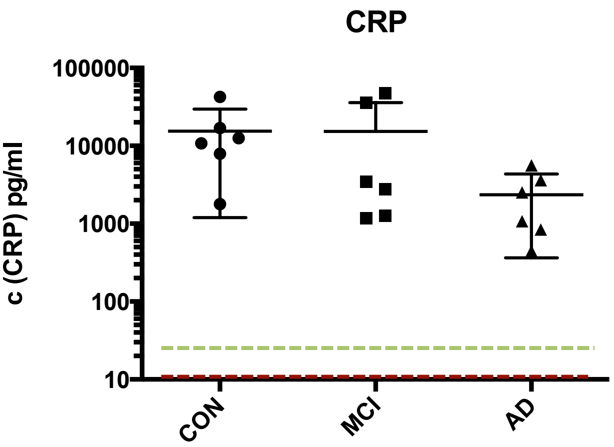

iv. Single analytes results

bFGF (electrochemiluminescence multiplex; angiogenesis panel)

Evaluation: Not passed

Samples not detectable

- Spike-In-Curve has small but positive difference to standard curve
- Spike-In concentrations are nonlinear
- Sample concentrations < LLOQ (2.6 pg/ml)
- No significant difference between test samples; samples < LLOQ in all groups

A) Spike In Curve

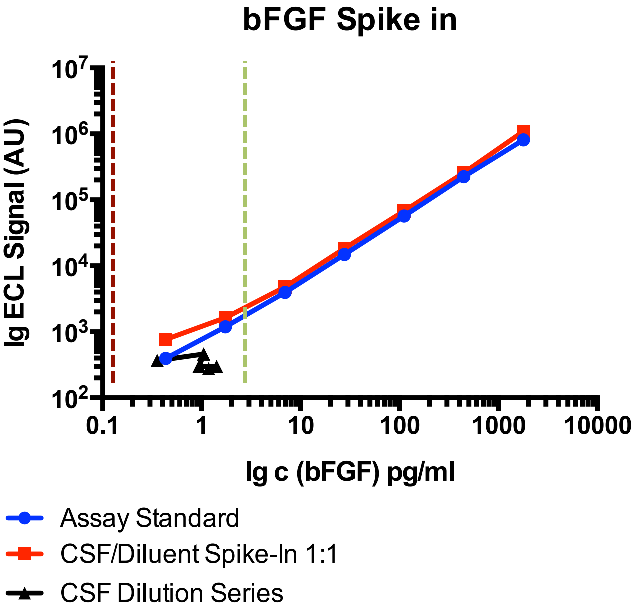

B) Recalculated concentrations curve

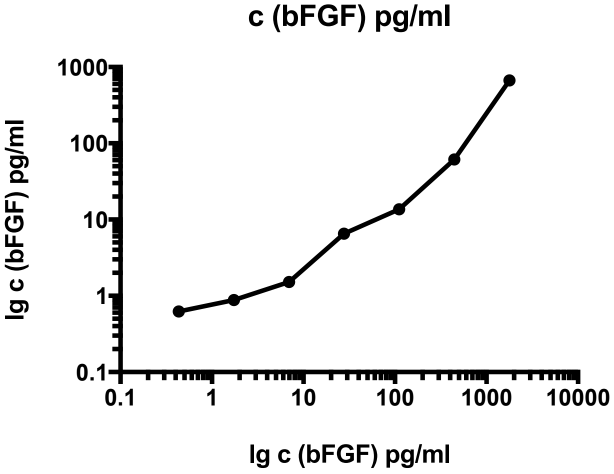

C) CSF dilution series

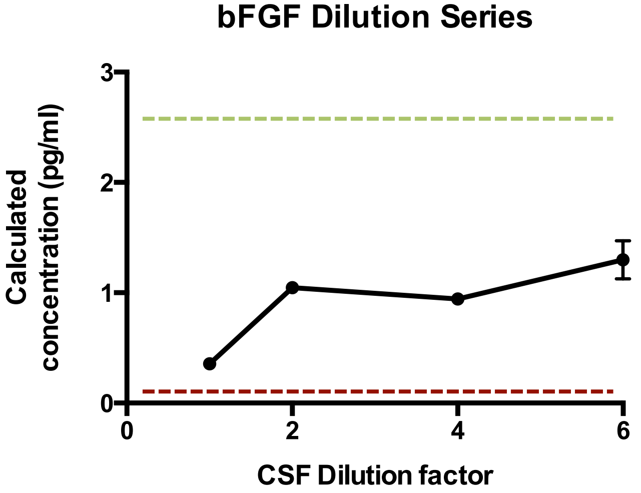

D) Test sample results

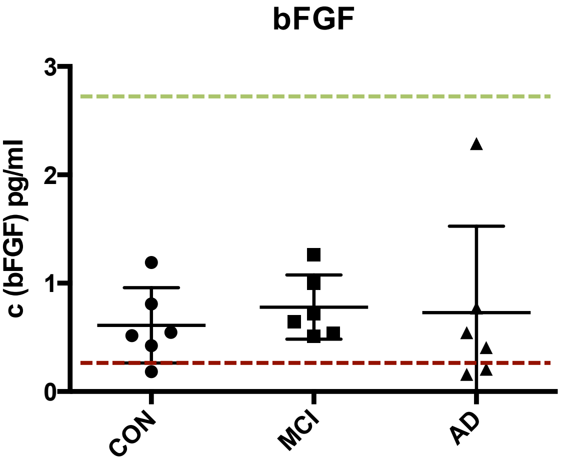

iv. Single analytes results

Eotaxin (electrochemiluminescence multiplex; chemokine panel)

Evaluation: Not passed

Samples not detectable

- Spike-In-Curve overlaps with standard curve
- Spike-In Concentrations are nonlinear
- Test sample concentrations overlap with LLOQ (12.3 pg/ml) in all groups
- No significant difference between test samples

A) Spike In Curve

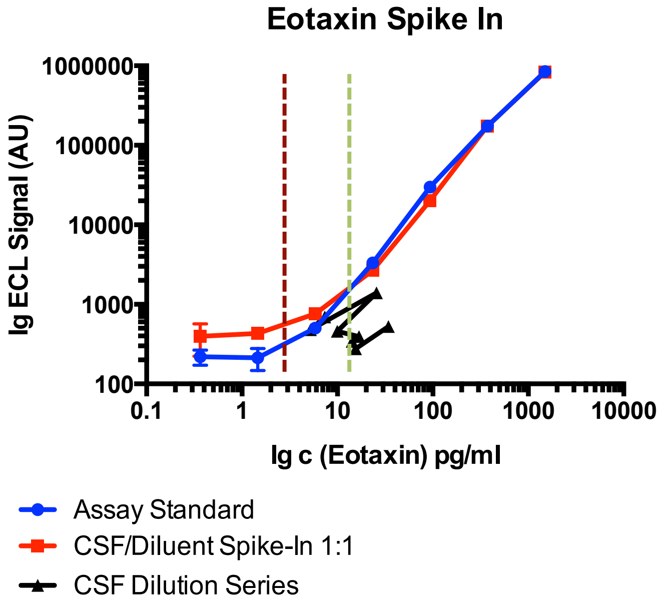

B) Recalculated concentrations curve

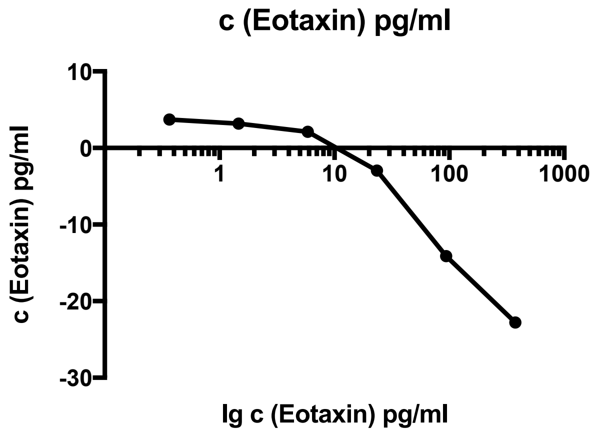

C) CSF dilution series

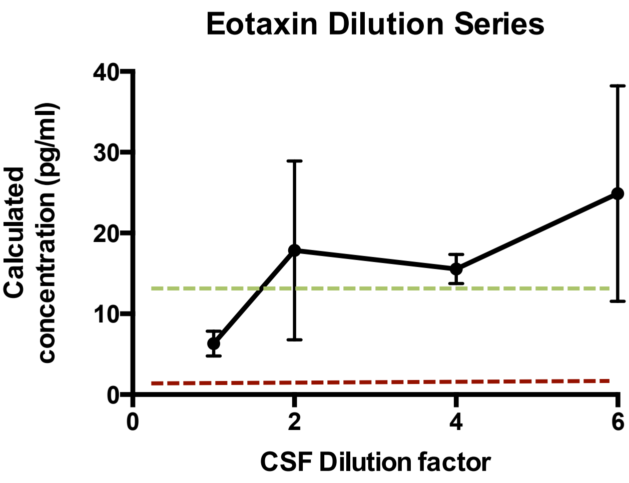

D) Test sample results

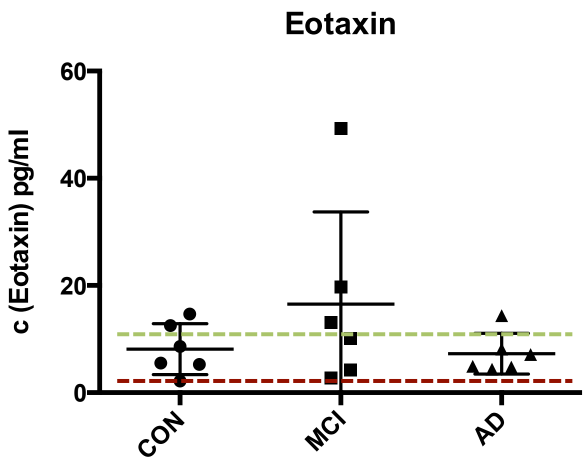

iv. Single analytes results

Eotaxin-3 (electrochemiluminescence multiplex; chemokine panel)

Evaluation: Not passed

Samples not detectable

- Spike-In-Curve overlaps with standard curve
- Spike-In Concentrations are nonlinear
- Test sample concentrations < LLOQ (10.2 pg/ml) in all groups
- No significant difference between test samples

A) Spike In Curve

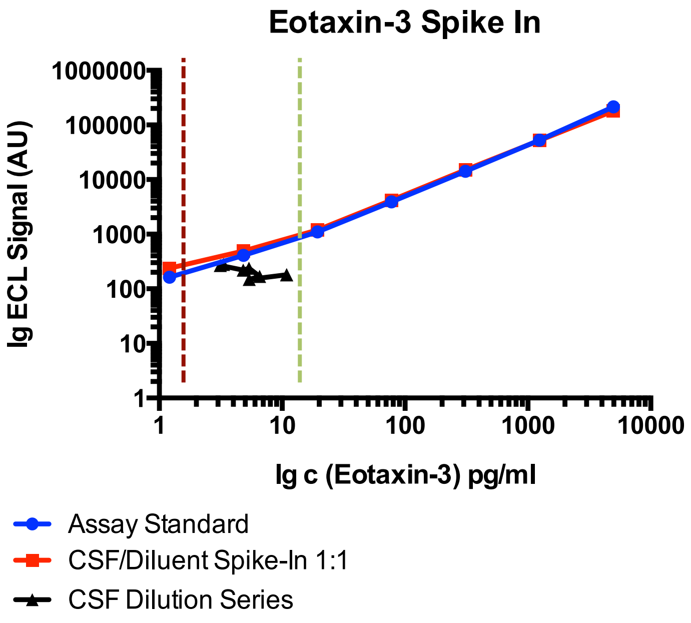

B) Recalculated concentrations curve

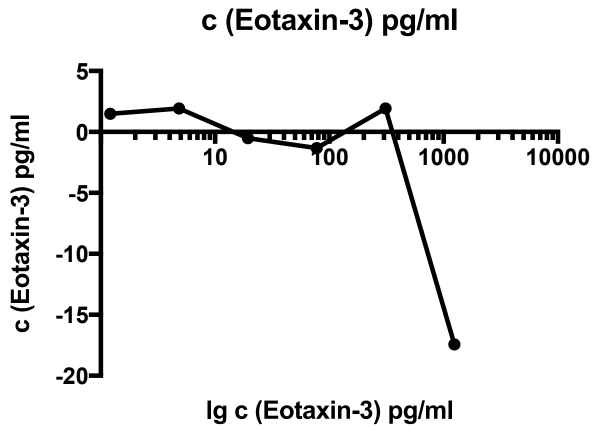

C) CSF dilution series

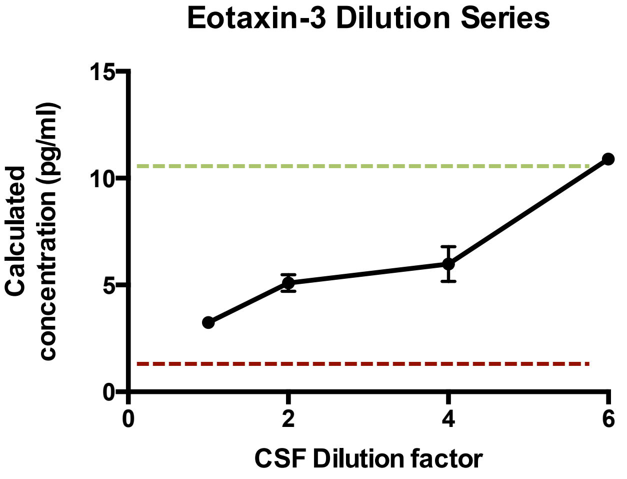

D) Test sample results

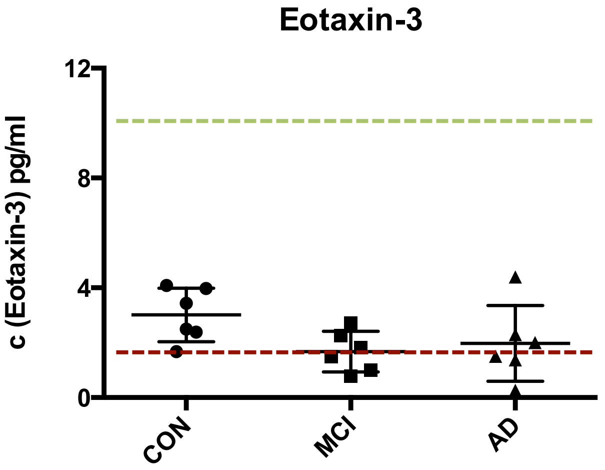

iv. Single analytes results

Flt-1/VEGFR (electrochemiluminescence multiplex; angiogenesis panel)

Evaluation: Passed

Optimal dilution factor is 2x

- Spike-In-Curve partly overlaps with standard curve
- Spike in Concentrations are linear in range of standard 4 – 8 at 10 pg/ml (2x Dilution)
- Sample concentrations > LLOQ (10.0 pg/ml)
- Sample concentrations in linear range
- No significant difference between test samples

A) Spike In Curve

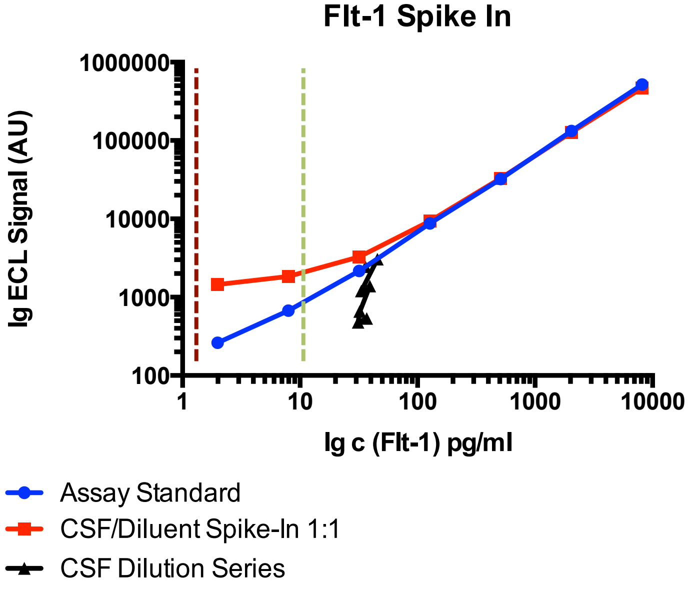

B) Recalculated concentrations curve

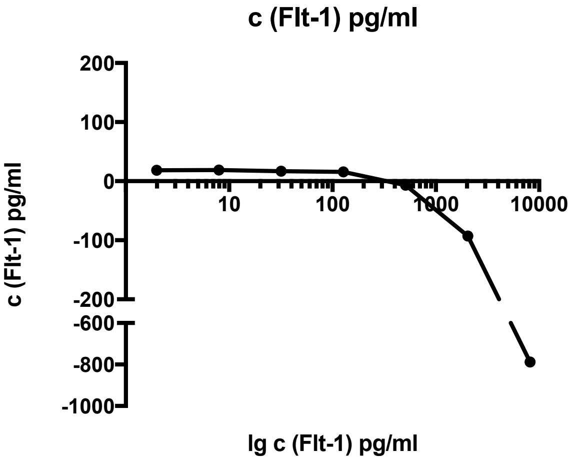

C) CSF dilution series

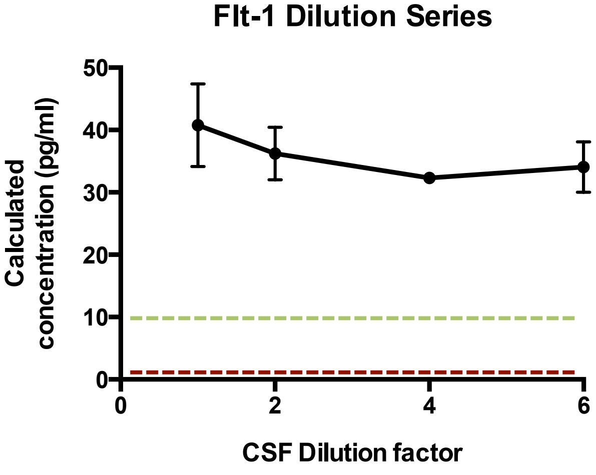

D) Test sample results

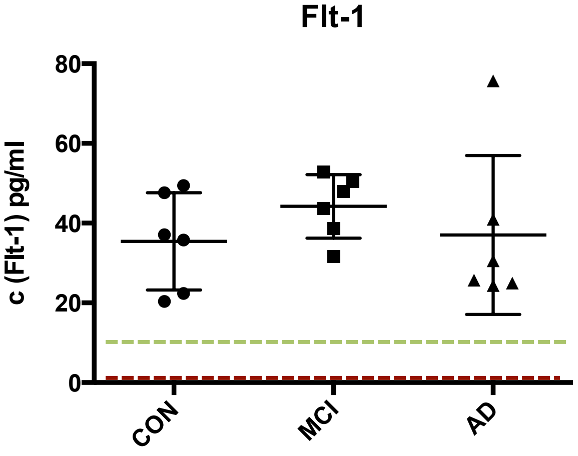

iv. Single analytes results

GM-CSF (electrochemiluminescence multiplex; cytokine panel)

Evaluation: Not passed

Samples not detectable

- Spike-In-Curve overlaps with standard curve
- Spike in Concentrations close to 0 in range of standard 3 – 8 (2x Dilution)
- Sample concentrations < LLOQ (1.9 pg/ml)
- No significant difference between test samples

A) Spike In Curve

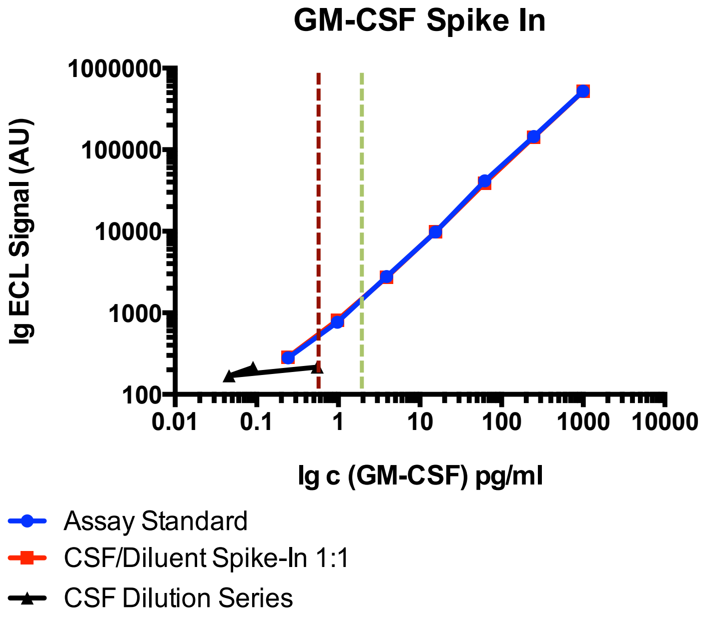

B) Recalculated concentrations curve

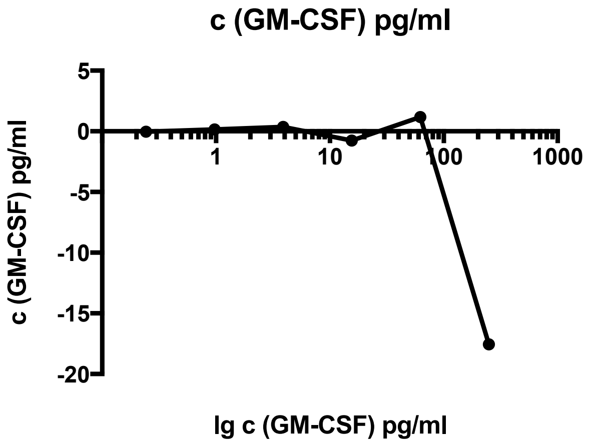

C) CSF dilution series

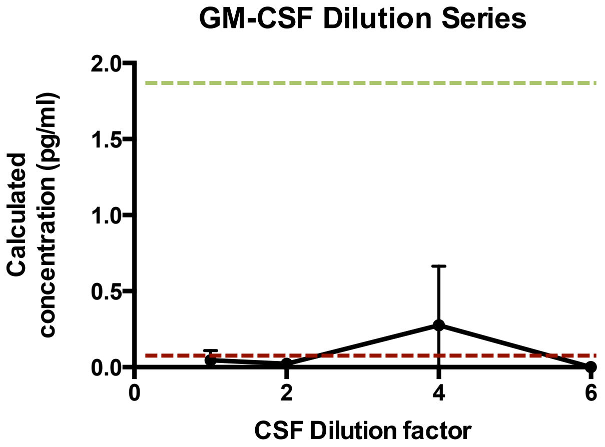

D) Test sample results

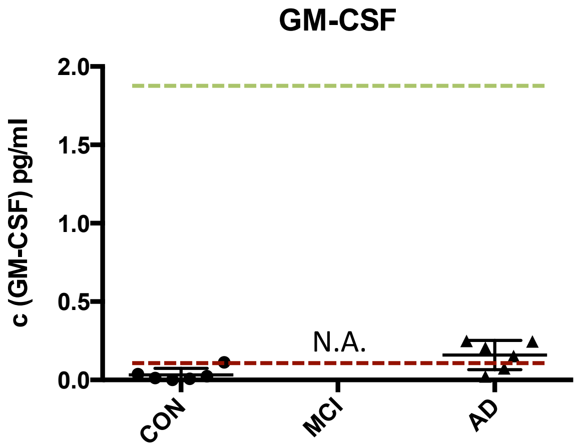

iv. Single analytes results

ICAM-1 (electrochemiluminescence multiplex; vascular injury panel)

Evaluation: Passed

Optimal dilution factor is 6x

- Spike-In-Curve has positive difference to standard curve
- Spike in Concentrations are linear in range of standard 3 – 8 at 10.000 pg/ml (2x Dilution)
- Sample concentrations > LLOQ (6.4 pg/ml)
- Sample concentrations in linear range
- All QC parameters passed
- No significant difference between test samples

A) Spike In Curve

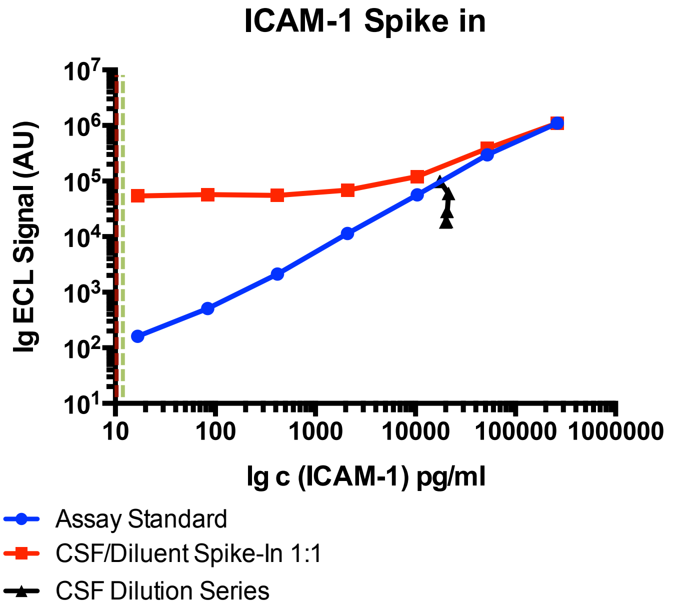

B) Recalculated concentrations curve

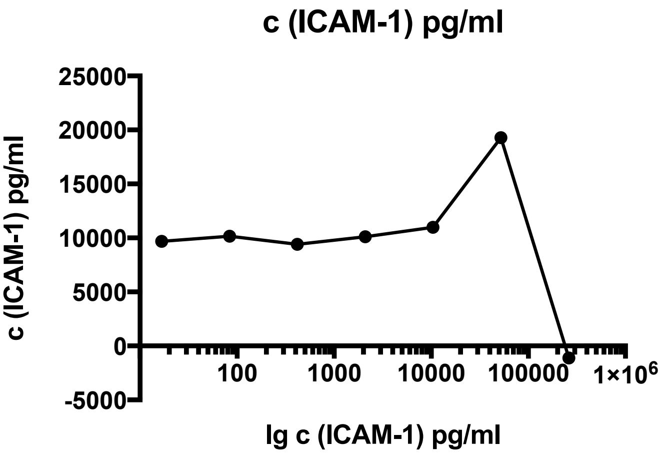

C) CSF dilution series

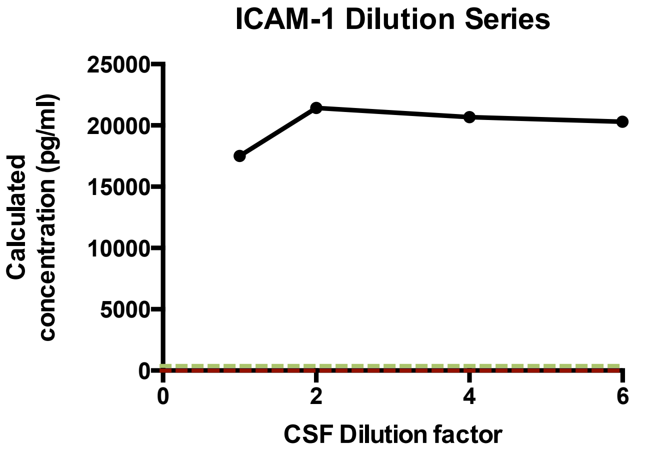

D) Test sample results

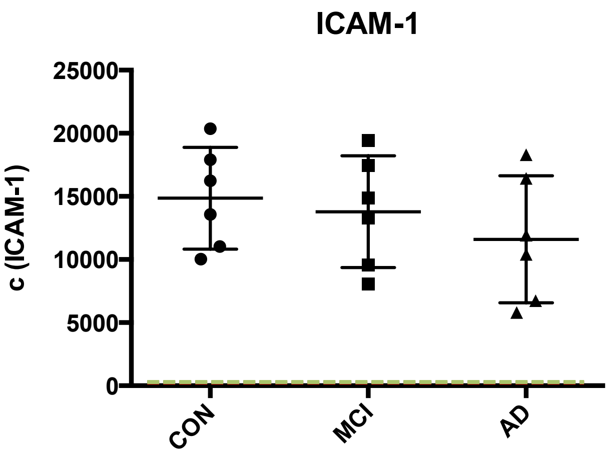

iv. Single analytes results

IFN-γ (electrochemiluminescence multiplex; proinflammatory panel)

Evaluation: Not passed

Samples not detectable

- Spike-In-Curve overlaps with standard curve
- Spike in Concentrations are nonlinear
- Sample concentrations < LLOQ (7.47 pg/ml)
- No significant difference between test samples

A) Spike In Curve

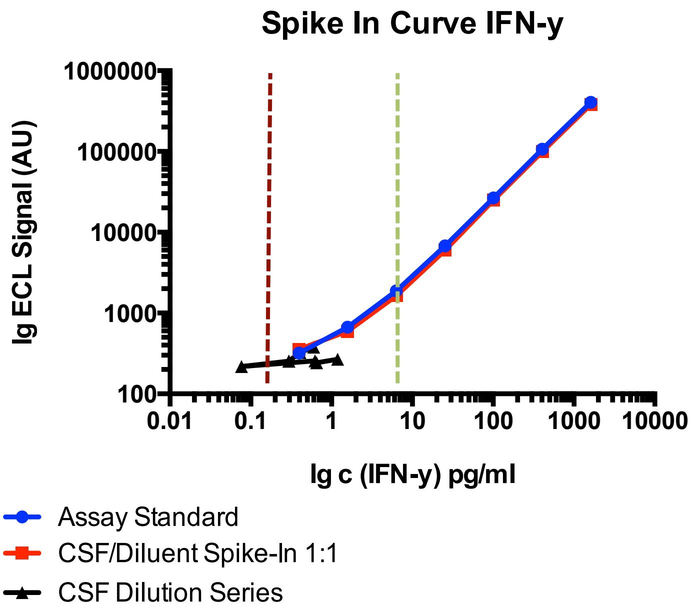

B) Recalculated concentrations curve

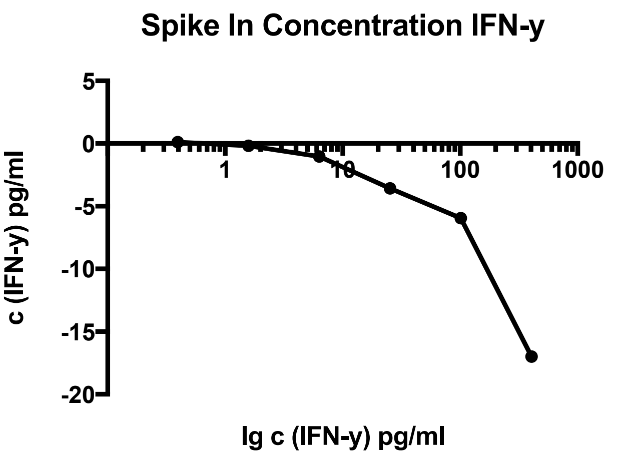

C) CSF dilution series

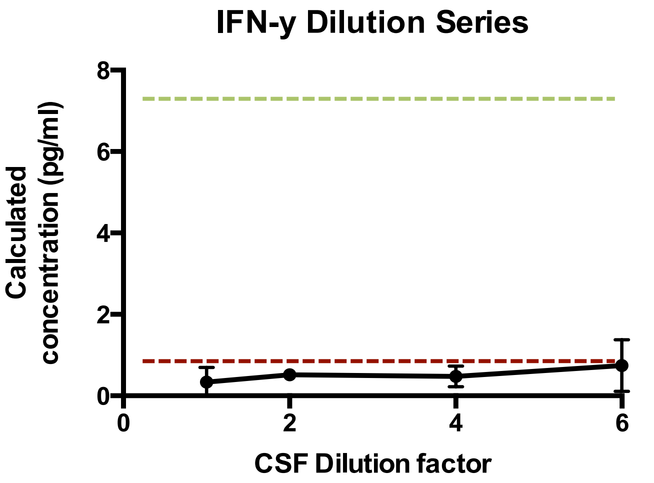

D) Test sample results

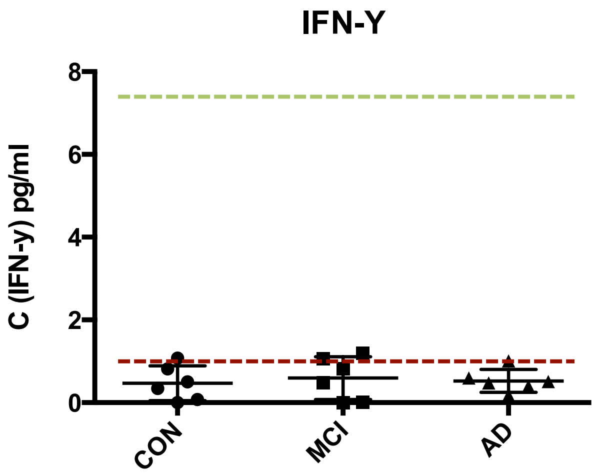

iv. Single analytes results

Il-1α (electrochemiluminescence multiplex; cytokine panel)

Evaluation: Not passed

Samples not detectable

- Spike-In-Curve overlaps with standard curve
- Spike in Concentrations are nonlinear
- Sample concentrations < LLOQ (2.85 pg/ml)
- No significant difference between test samples

A) Spike In Curve

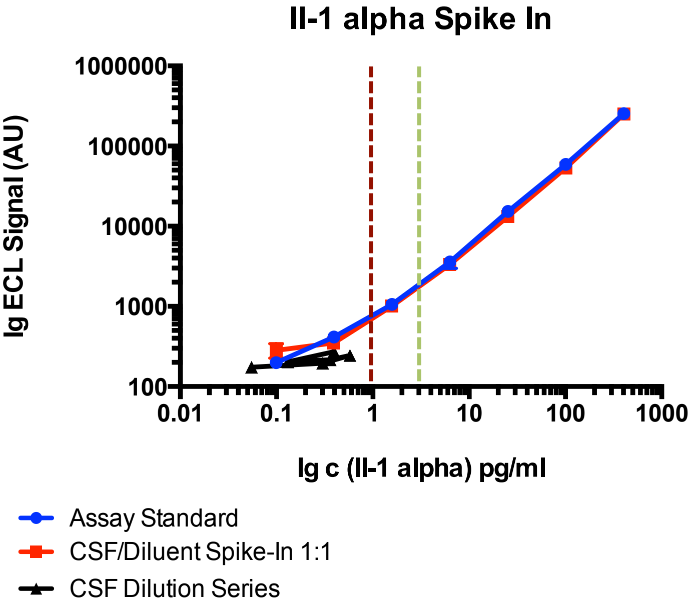

B) Recalculated concentrations curve

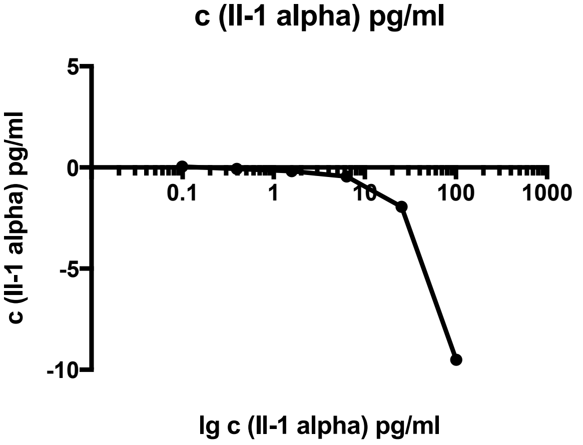

C) CSF dilution series

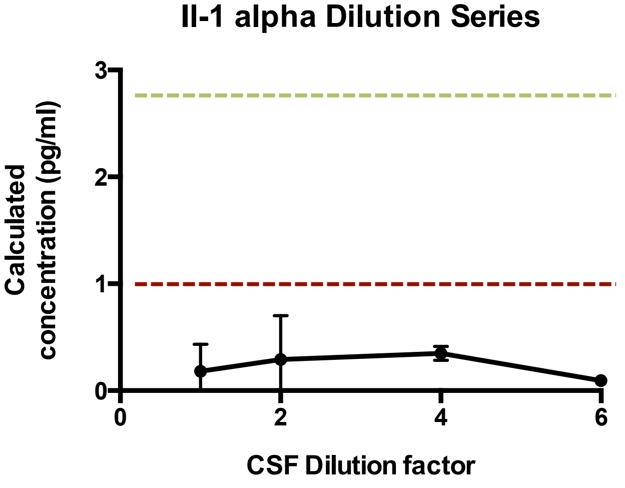

D) Test sample results

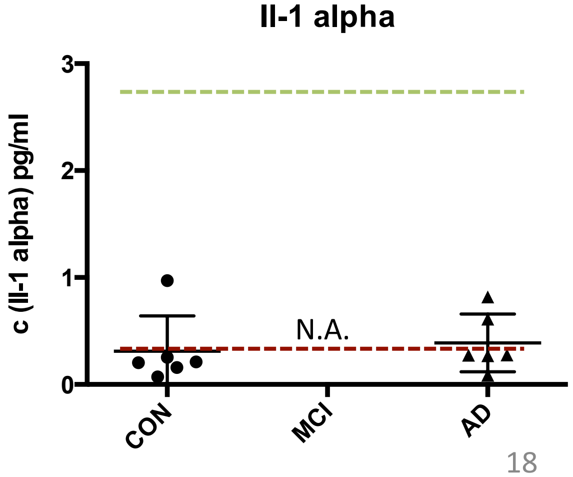

iv. Single analytes results

Il-1β (electrochemiluminescence multiplex; proinflammatory panel)

Evaluation: Not passed

Samples not detectable

- Spike-In-Curve overlaps with standard curve
- Spike in Concentrations are nonlinear
- Sample concentrations < LLOD (0.04 pg/ml)
- No significant difference between test samples

A) Spike In Curve

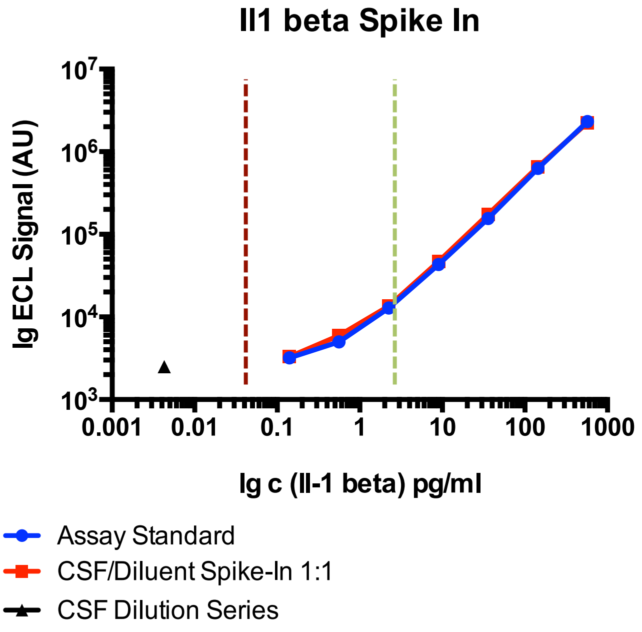

B) Recalculated concentrations curve

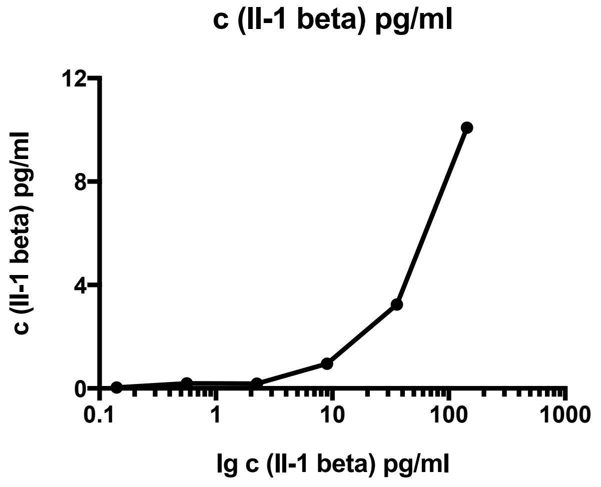

C) CSF dilution series

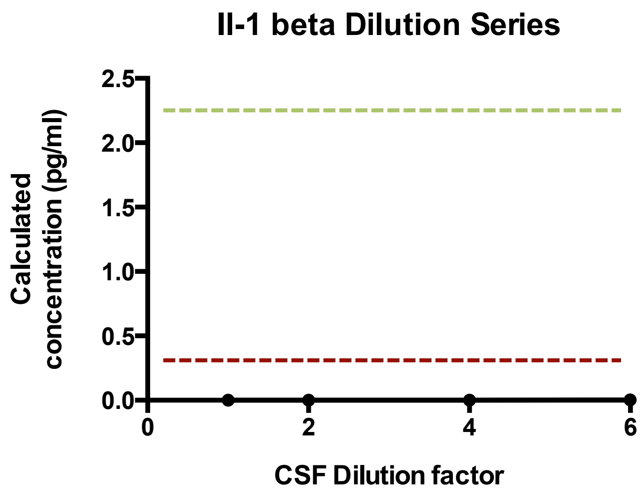

D) Test sample results

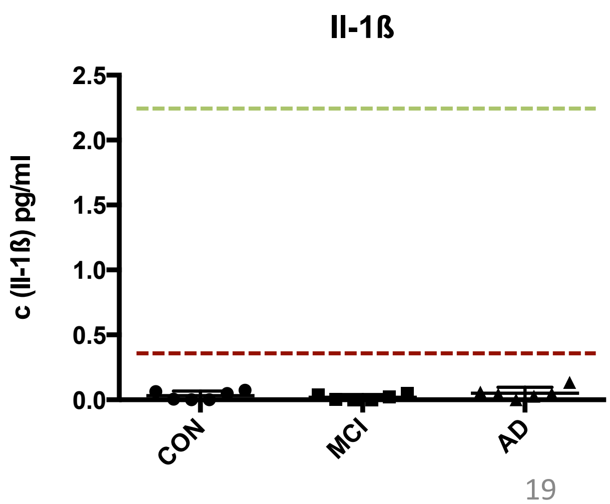

iv. Single analytes results

Il-1RAcP (colorimetric singleplex)

Evaluation: Passed

Optimal dilution factor is 50x

- Spike-In-Curve has positive difference to standard curve
- Spike in Concentrations linear in range of standard 5 – 8 at 300 pg/ml (50x Dilution)
- Sample concentrations in linear range
- 2 test samples have a CV >20% while average CV is <10%
- No significant difference between test samples

A) Spike In Curve

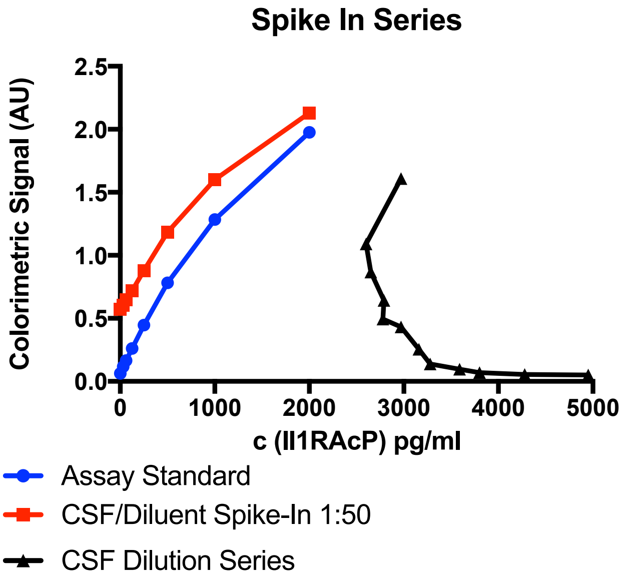

B) Recalculated concentrations curve

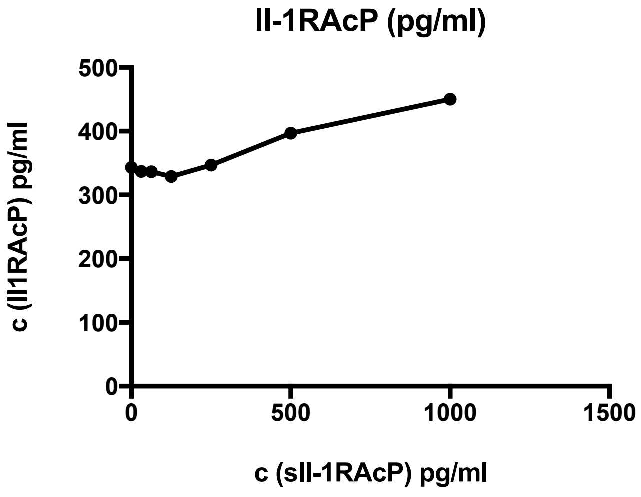

C) CSF dilution series

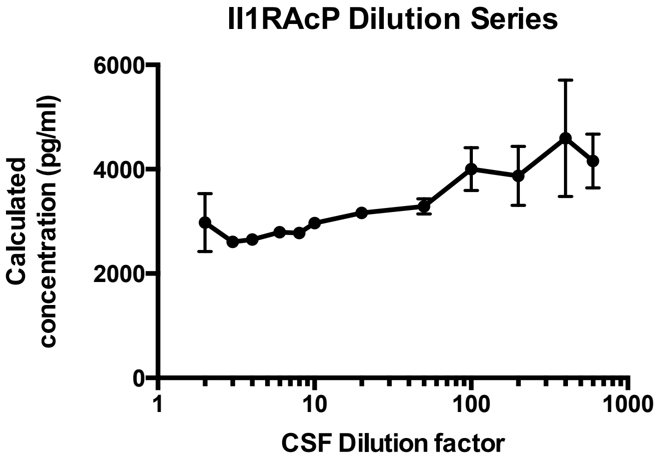

D) Test sample results

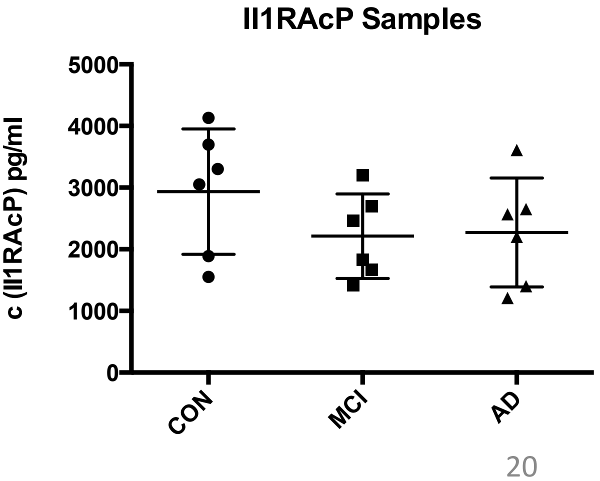

iv. Single analytes results

Il-2 (electrochemiluminescence multiplex; proinflammatory panel)

Evaluation: Not passed

Samples not detectable

- Spike-In-Curve has small but positive difference to standard curve
- Spike-in concentrations are nonlinear
- Sample concentrations < LLOQ (0.89 pg/ml)
- No significant difference between test samples

A) Spike In Curve

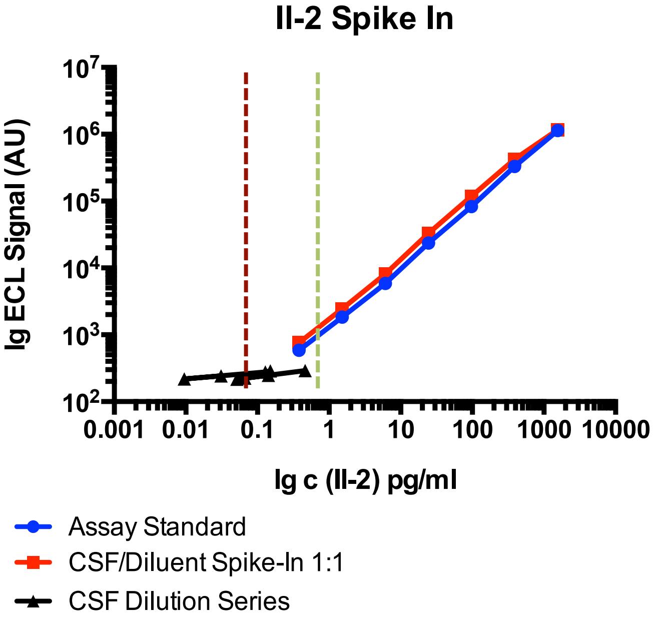

B) Recalculated concentrations curve

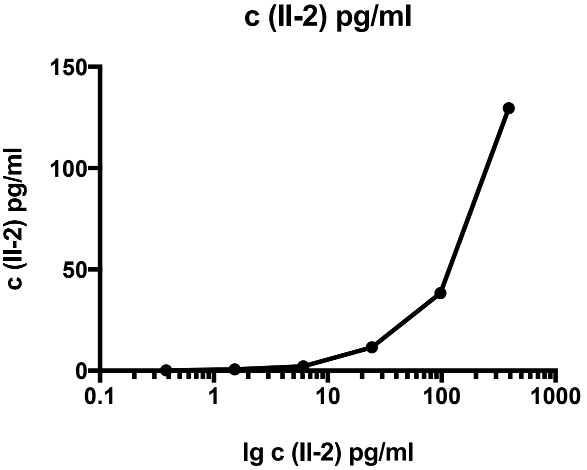

C) CSF dilution series

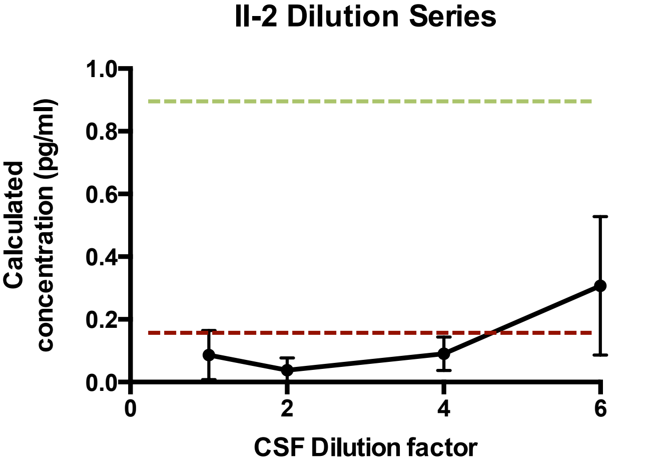

D) Test sample results

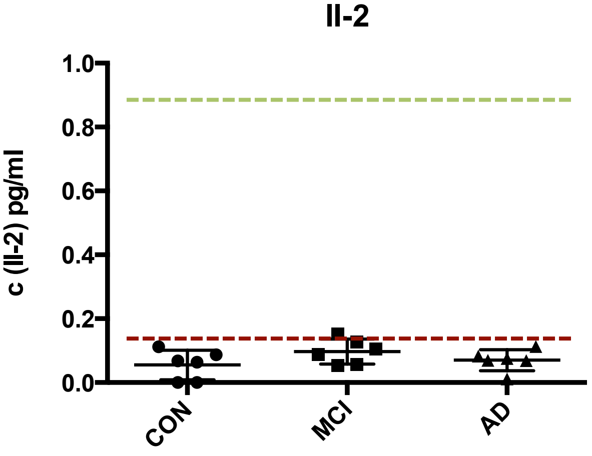

iv. Single analytes results

Il-4 (electrochemiluminescence multiplex; proinflammatory panel)

Evaluation: Not passed

Samples not detectable

- Spike-In-Curve overlaps with standard curve
- Spike in Concentrations are nonlinear
- Sample concentrations < LLOD (0.02 pg/ml)
- No significant difference between test samples

A) Spike In Curve

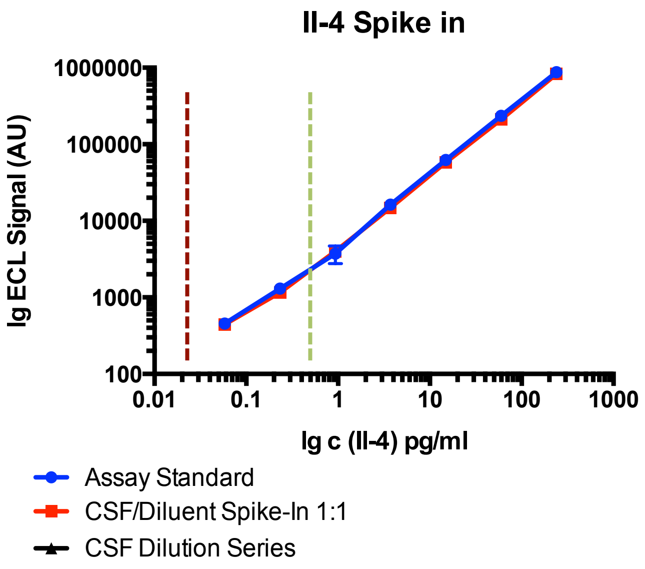

B) Recalculated concentrations curve

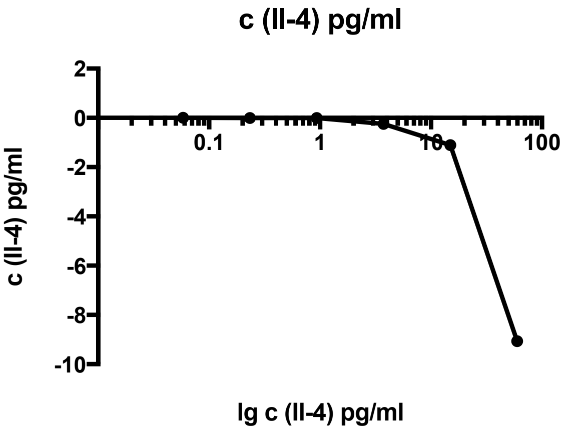

C) CSF dilution series

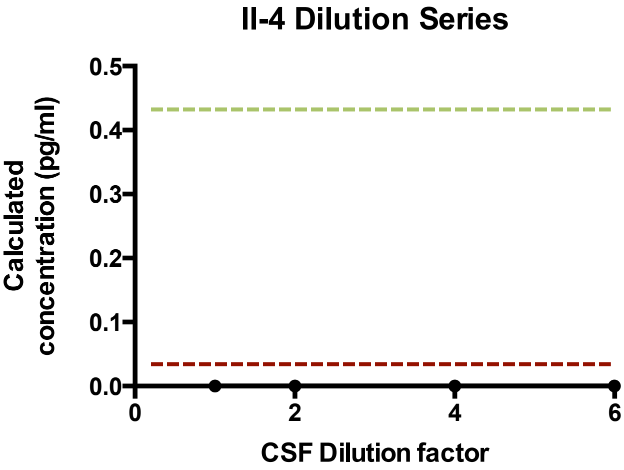

D) Test sample results

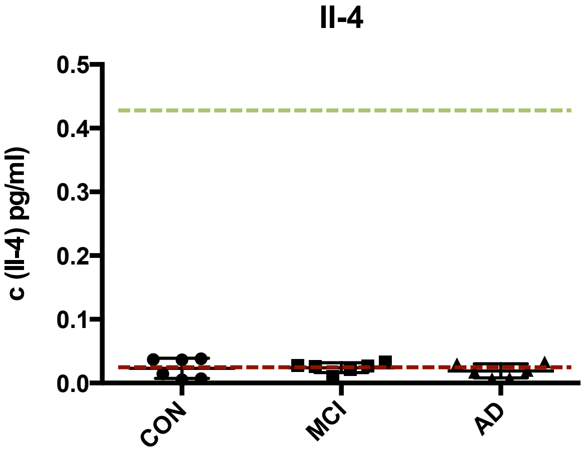

iv. Single analytes results

Il-5 (electrochemiluminescence multiplex; cytokine panel)

Evaluation: **Borderline**

Samples not detectable but might be drawn in detection range by spike-in of protein

- Spike-In-Curve overlaps with standard curve
- Spike in Concentrations are linear in range of standard 5 – 8 at 0.5 pg/ml (2x Dilution)
- Sample concentrations < LLOQ (6.28 pg/ml)
- No significant difference between test samples

A) Spike In Curve

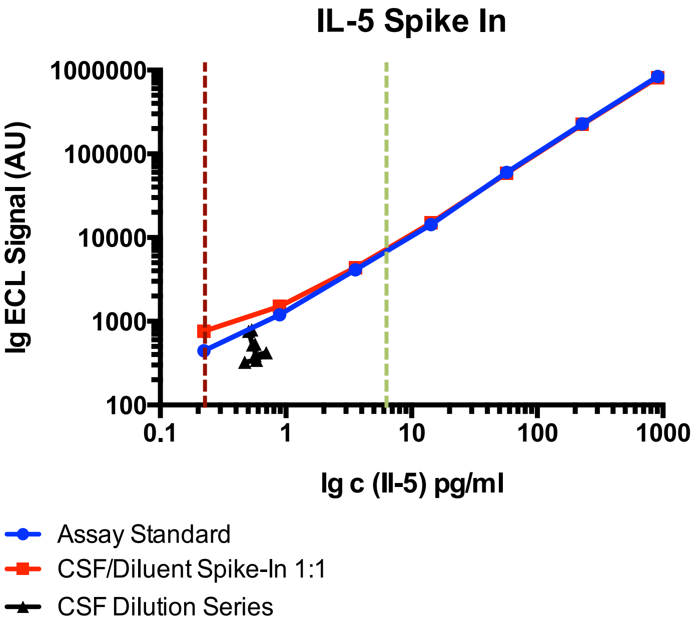

B) Recalculated concentrations curve

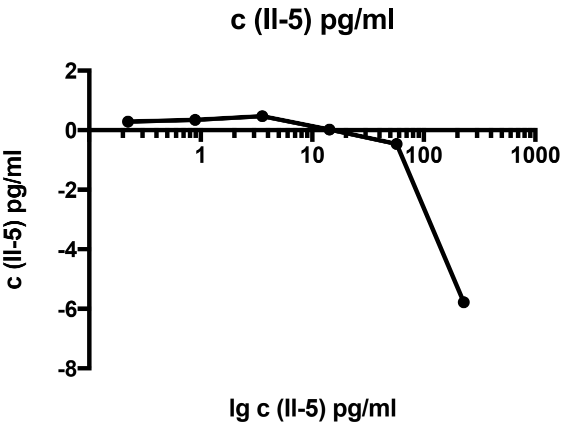

C) CSF dilution series

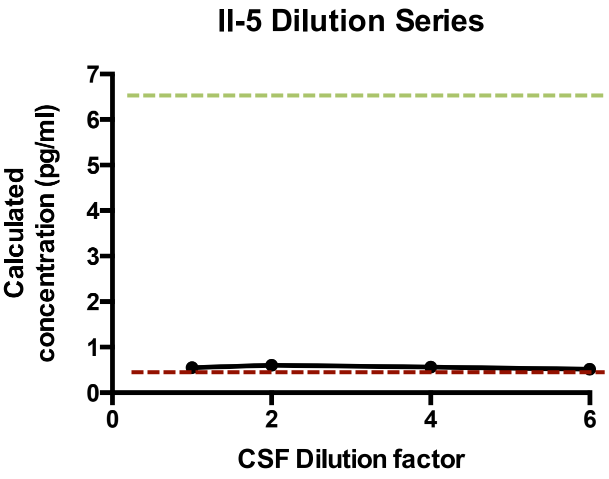

D) Test sample results

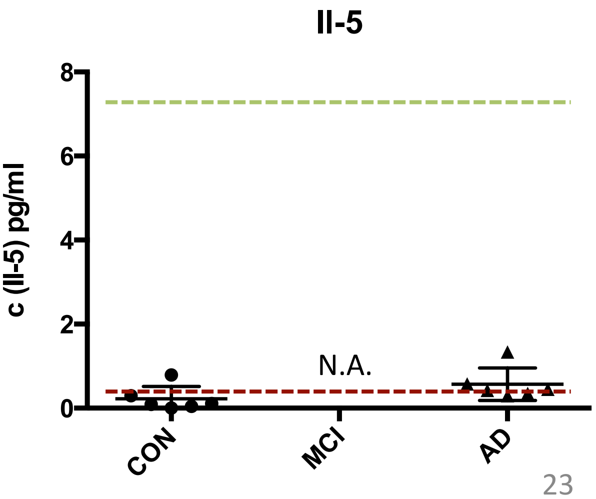

iv. Single analytes results

Il-6 (electrochemiluminescence multiplex; proinflammatory panel)

Evaluation: Borderline

Samples not detectable but might be drawn in detection range by spike-in of protein

- Spike-In-Curve has small but positive difference to standard curve
- Spike in Concentrations are linear in range of standard 4 – 8 at 10.0 pg/ml (2x Dilution)
- Sample concentrations < LLOQ (1.58 pg/ml)
- No significant difference between test samples

A) Spike In Curve

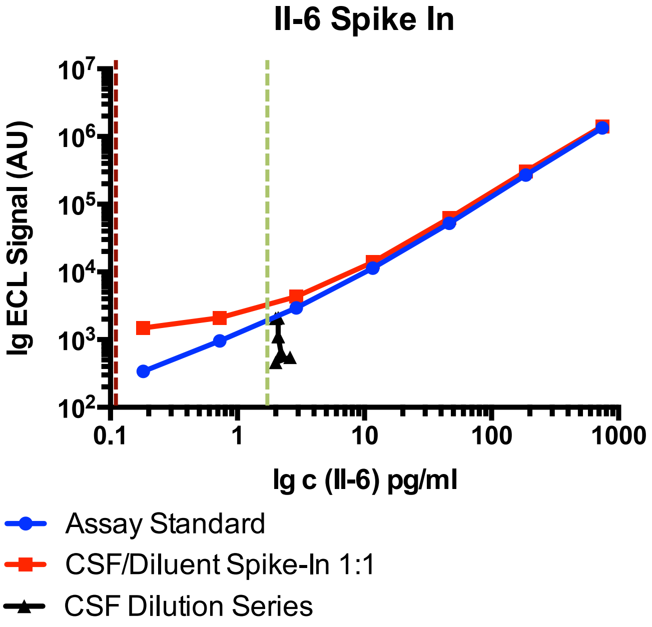

B) Recalculated concentrations curve

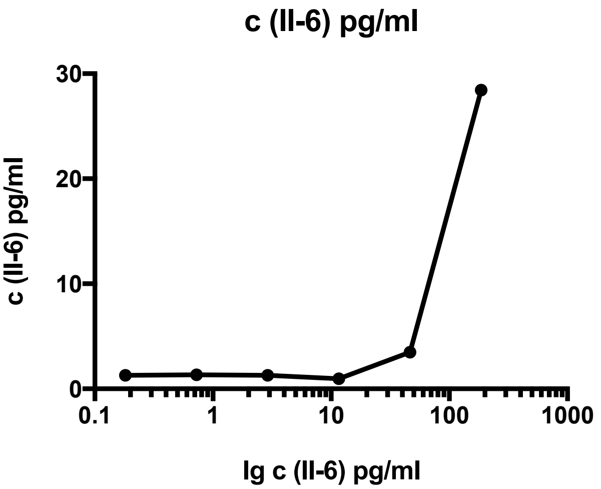

C) CSF dilution series

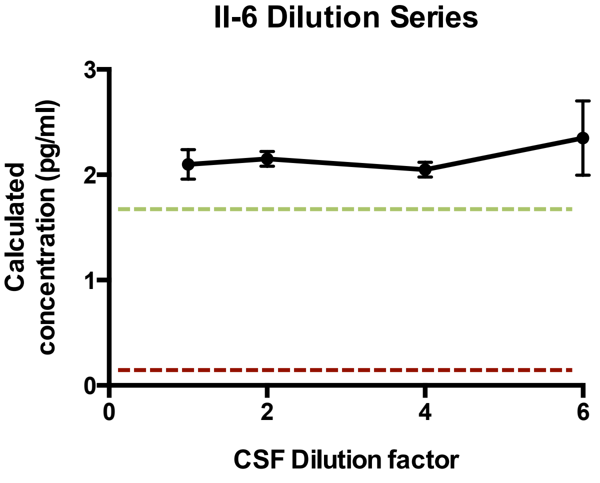

D) Test sample results

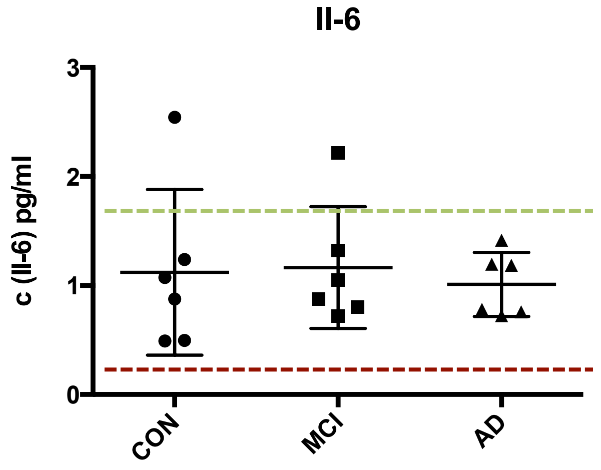

iv. Single analytes results

Il-7 (electrochemiluminescence multiplex; cytokine panel)

Evaluation: **Borderline**
Samples not detectable but might be drawn in detection range by spike-in of protein

- Spike-In-Curve overlaps with standard curve
- Spike in Concentrations are linear in range of standard 5 – 8 at 0.5 pg/ml (2x Dilution)
- Sample concentrations < LLOQ (1.37 pg/ml)
- Sample concentrations below assay range
- Conflicting QC:
    - Majority of samples < LLOQ
    - Majority of samples still has low CV (75% of samples has CV < 20%)
    - Tendency is not significant in nonparametric test
- No significant difference between test samples

A) Spike In Curve

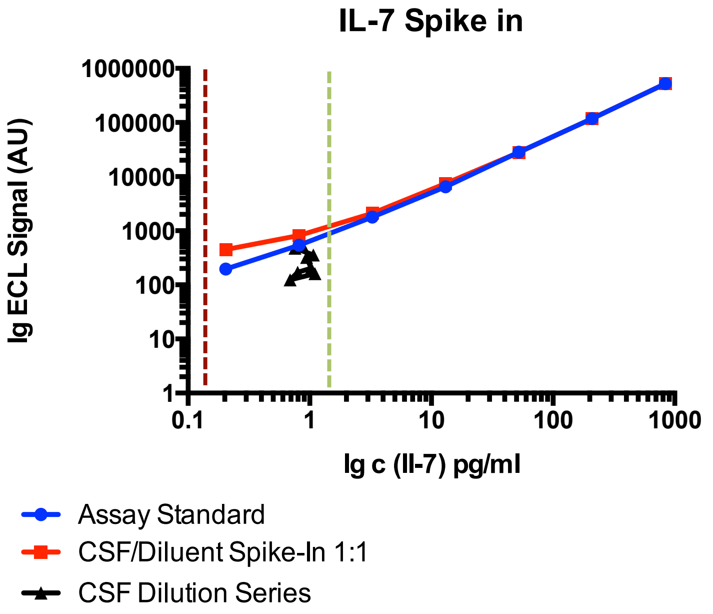

B) Recalculated concentrations curve

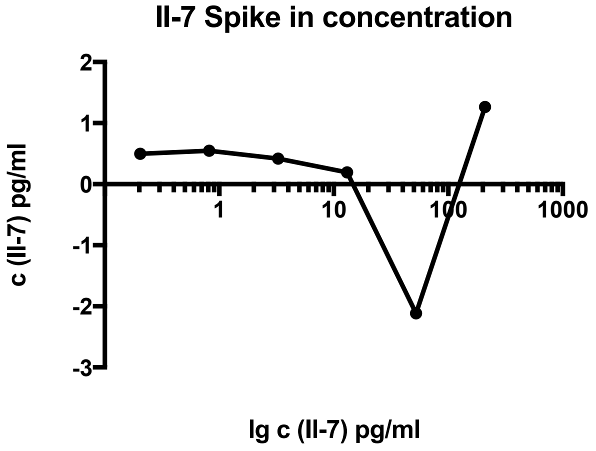

C) CSF dilution series

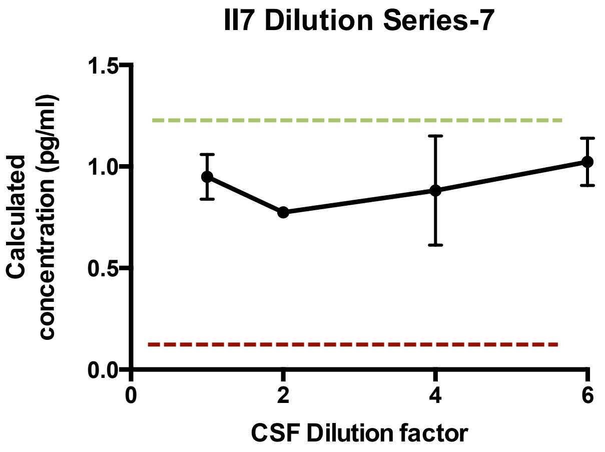

D) Test sample results

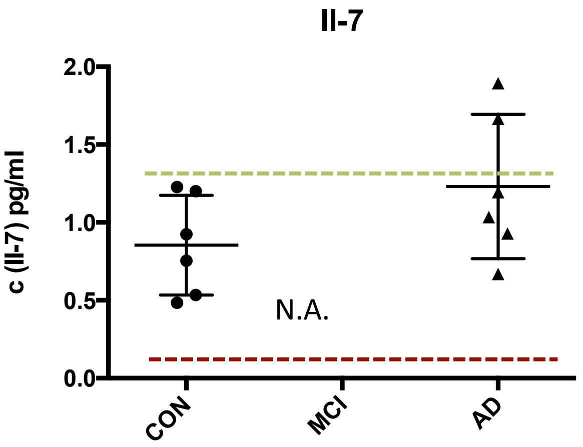

iv. Single analytes results

Il-8 (electrochemiluminescence multiplex; chemokine panel)

Note: This assay was also tested as part of the proinflammatory panel (p. 27) were it passed due to differences in concentration range
Evaluation: Not passed

Samples not detectable (use proinflammatory panel version instead)

- Spike-In-Curve overlaps with standard curve
- Spike in Concentrations are nonlinear
- Sample concentrations < LLOQ (713.0 pg/ml)
- No significant difference between test samples

A) Spike In Curve

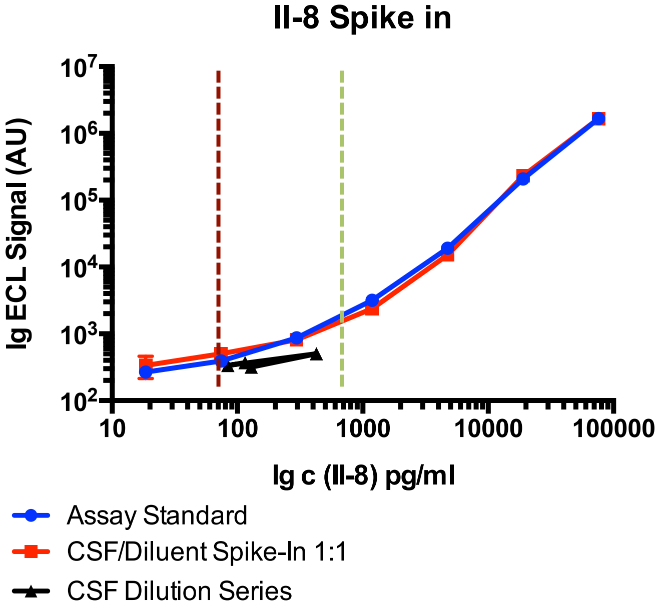

B) Recalculated concentrations curve

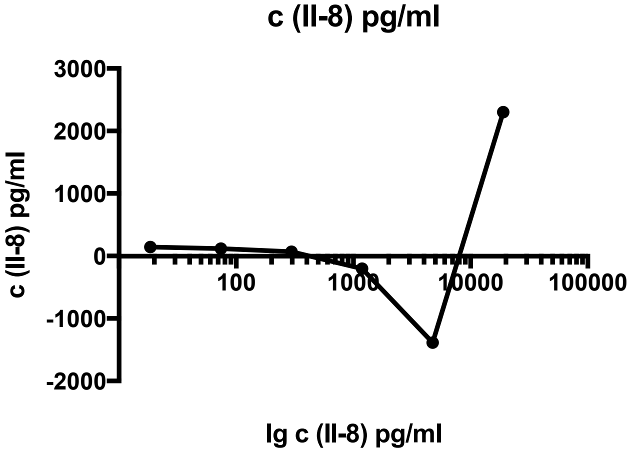

C) CSF dilution series

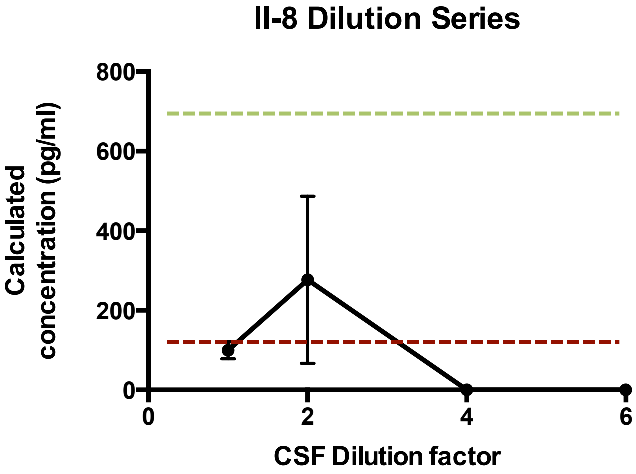

D) Test sample results

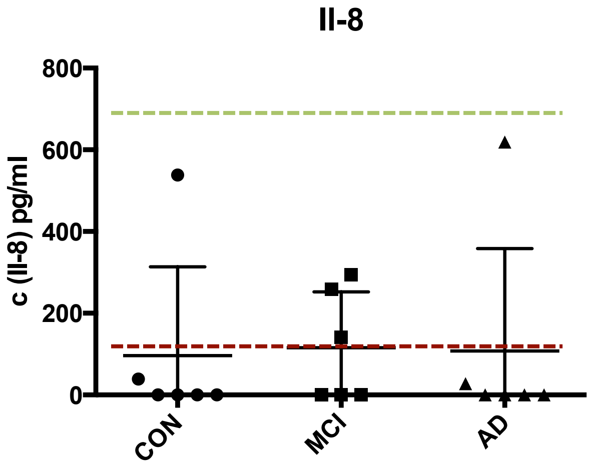

iv. Single analytes results

Il-8 (electrochemiluminescence multiplex; proinflammatory panel)

Note: This assay was also tested as part of the chemokine panel (p. 26) were it failed due to differences in concentration range
Evaluation: Passed

Optimal dilution factor is 4x

- Spike-In-Curve has positive difference to standard curve
- Spike in Concentrations are linear in range of standard 2 – 8 at 30 pg/ml (2x Dilution)
- Sample concentrations > LLOQ (1.13 pg/ml)
- Sample concentrations in linear range
- No significant difference between test samples

A) Spike In Curve

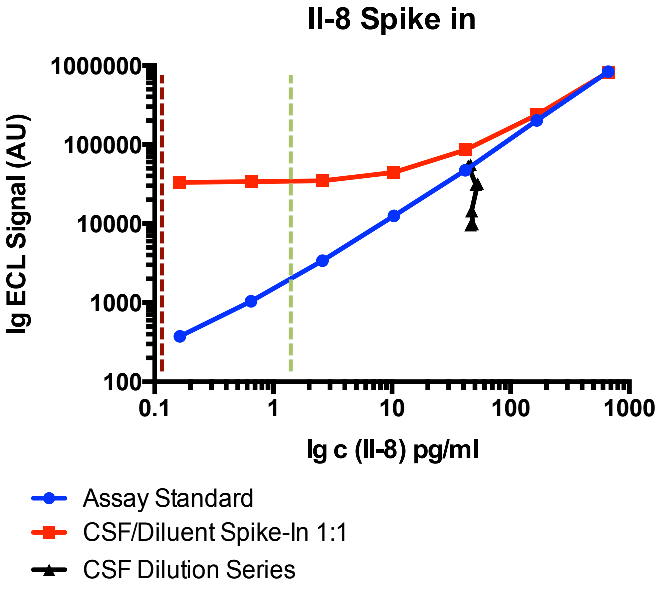

B) Recalculated concentrations curve

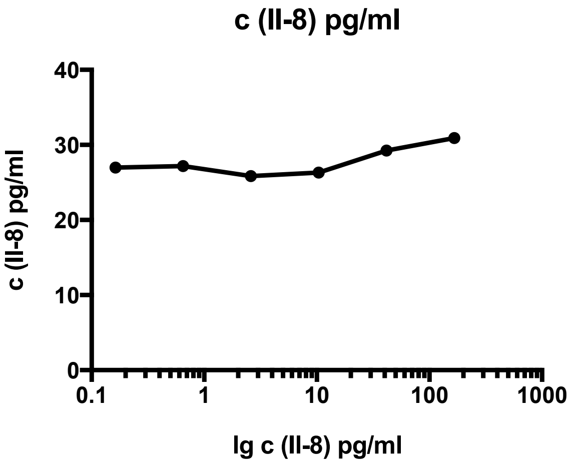

C) CSF dilution series

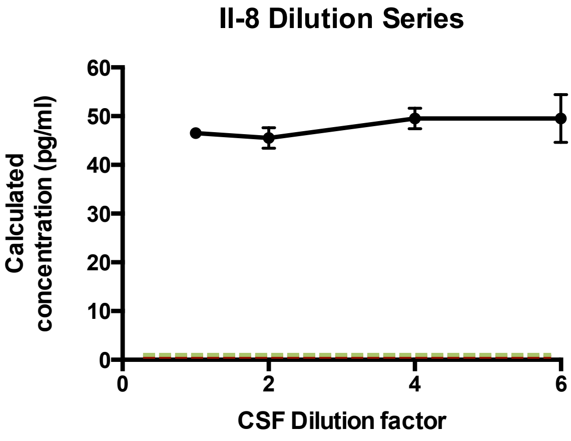

D) Test sample results

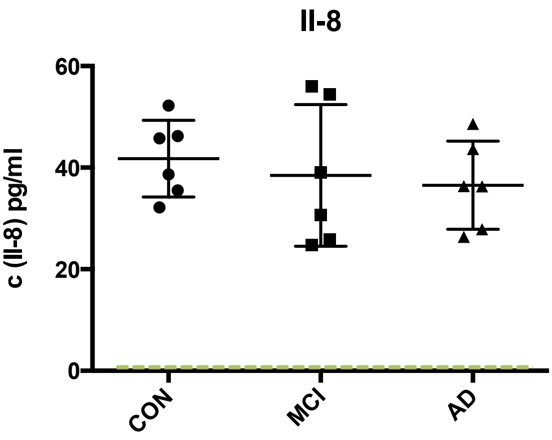

iv. Single analytes results

Il-10 (electrochemiluminescence multiplex; proinflammatory panel)

Evaluation: Not passed

Samples not detectable

- Spike-In-Curve overlaps with standard curve
- Spike in Concentrations close to 0 in range of standard 5 – 8 (2x Dilution)
- Sample concentrations < LLOQ (0.68 pg/ml)
- No significant difference between test samples

A) Spike In Curve

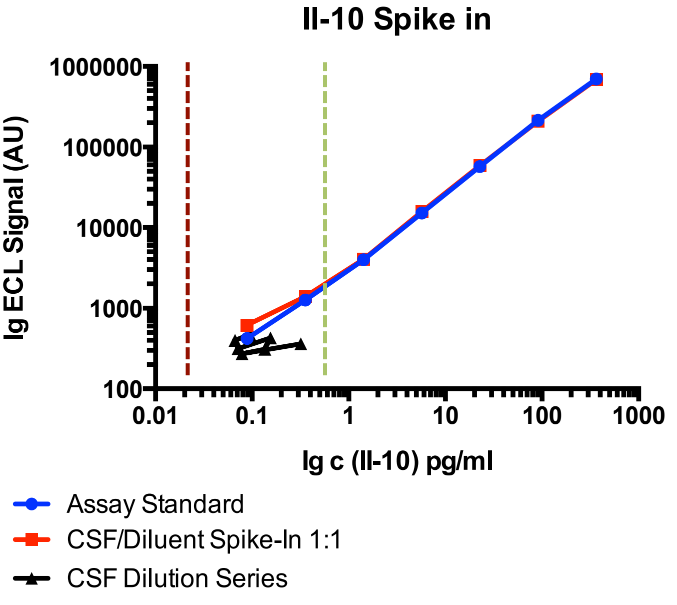

B) Recalculated concentrations curve

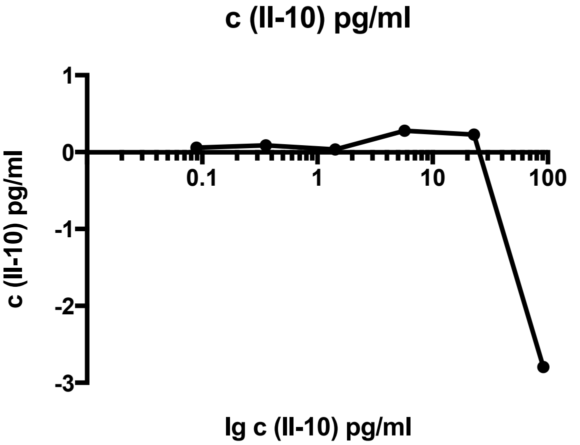

C) CSF dilution series

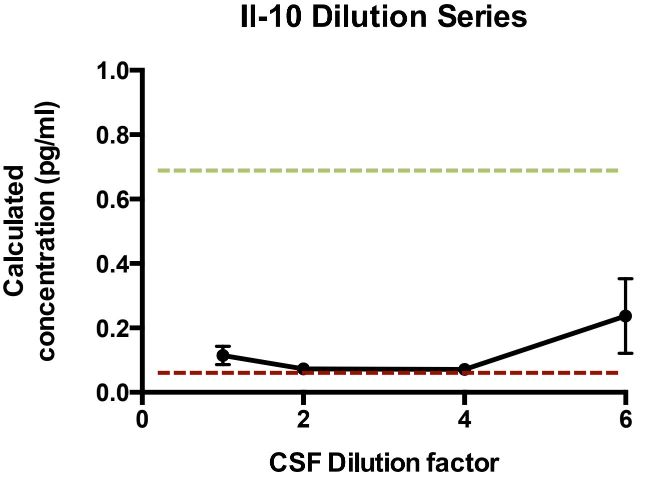

D) Test sample results

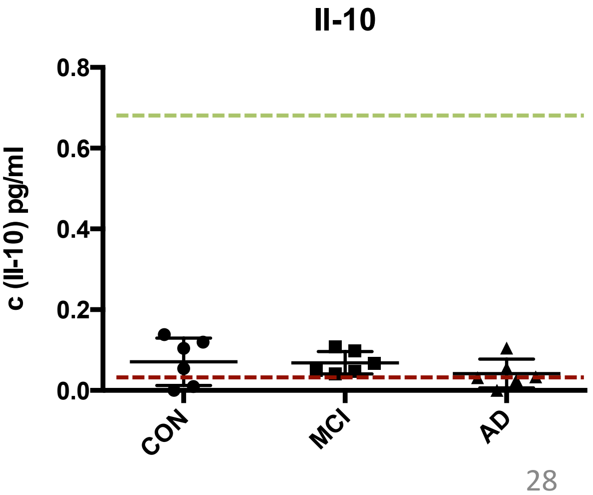

iv. Single analytes results

Il-12 p40 (electrochemiluminescence multiplex; cytokine panel)

Evaluation: Borderline
Samples not detectable but might be drawn in detection range by spike-in of protein

- Spike-In-Curve overlaps with standard curve
- Spike in Concentrations linear in range of standard 4 – 8 at 2.0 pg/ml (2x Dilution)
- Sample concentrations < LLOQ (5.68 pg/ml)
- Sample concentrations below assay range
- Conflicting QC:
  - Majority of samples < LLOQ
  - All tested samples still have low CV (100% of samples has CV < 20%)
  - No tendency between tested samples
- No significant difference between test samples

A) Spike In Curve

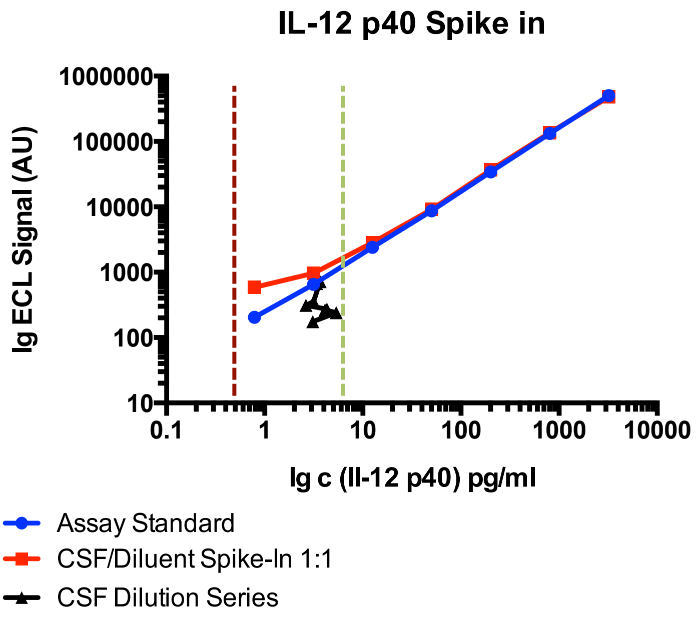

B) Recalculated concentrations curve

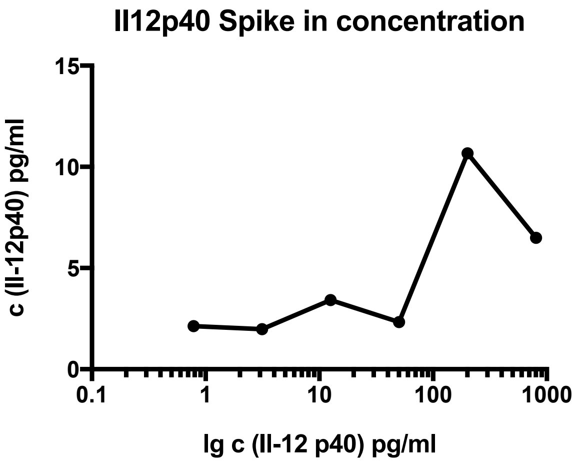

C) CSF dilution series

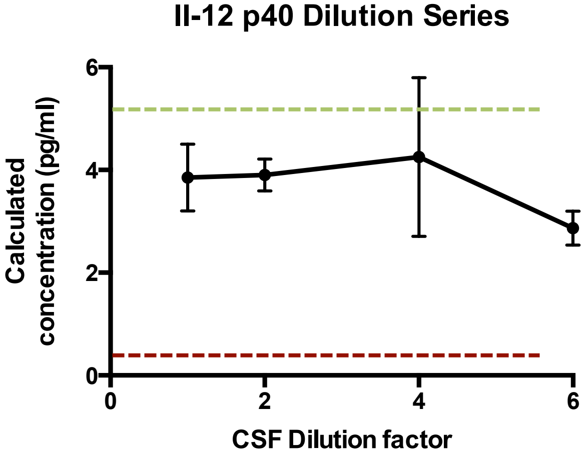

D) Test sample results

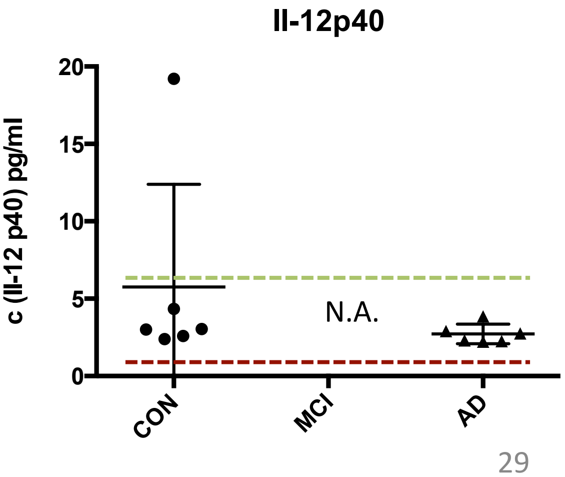

iv. Single analytes results

Il-12 p70 (electrochemiluminescence multiplex; proinflammatory panel)

Evaluation: Not passed

Samples not detectable

- Spike-In-Curve overlaps with standard curve
- Spike in Concentrations are nonlinear
- Sample concentrations < LLOQ (1.22 pg/ml)
- No significant difference between test samples

A) Spike In Curve

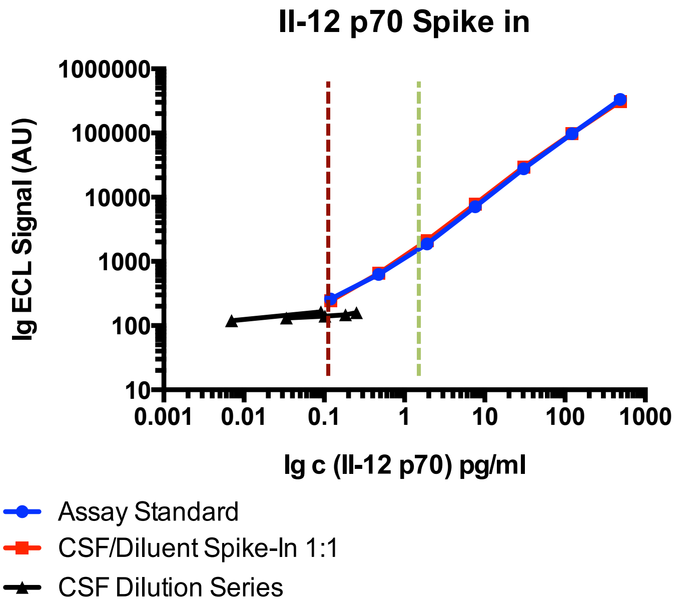

B) Recalculated concentrations curve

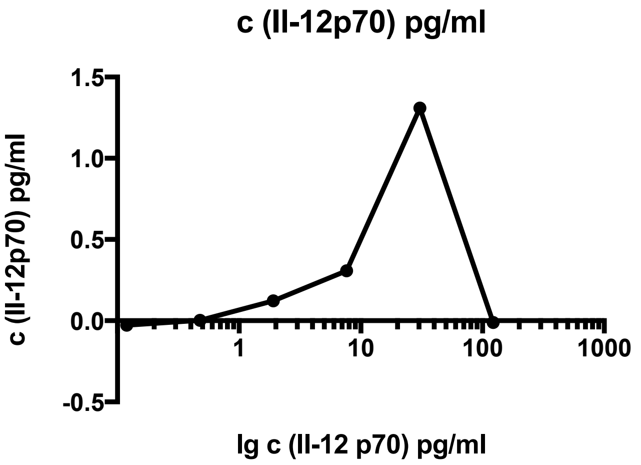

C) CSF dilution series

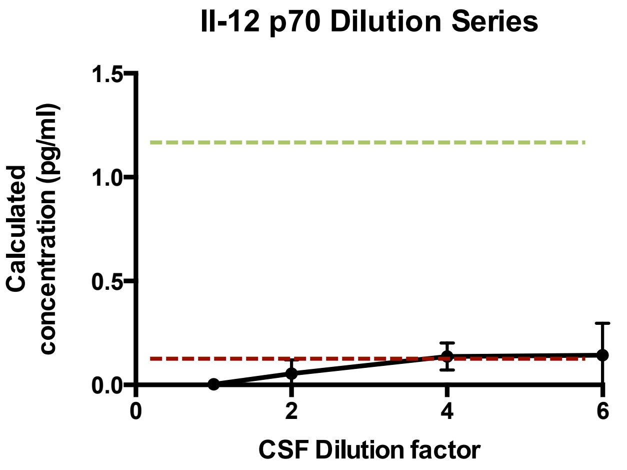

D) Test sample results

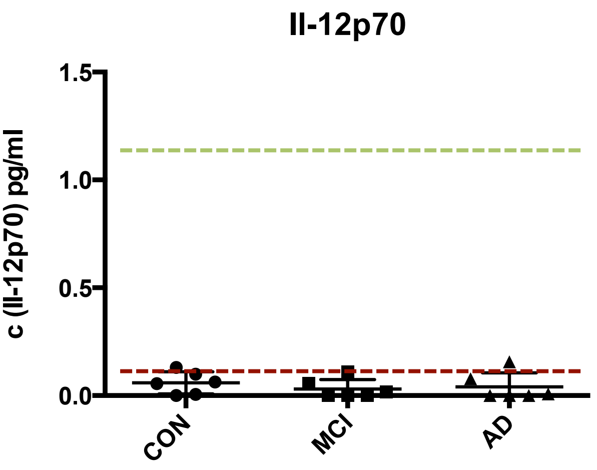

iv. Single analytes results

Il-13 (electrochemiluminescence multiplex; proinflammatory panel)

Evaluation: Not passed

Samples not detectable

- Spike-In-Curve overlaps with standard curve
- Spike in Concentrations are nonlinear
- Sample concentrations < LLOQ (4.21 pg/ml)
- No significant difference between test samples

A) Spike In Curve

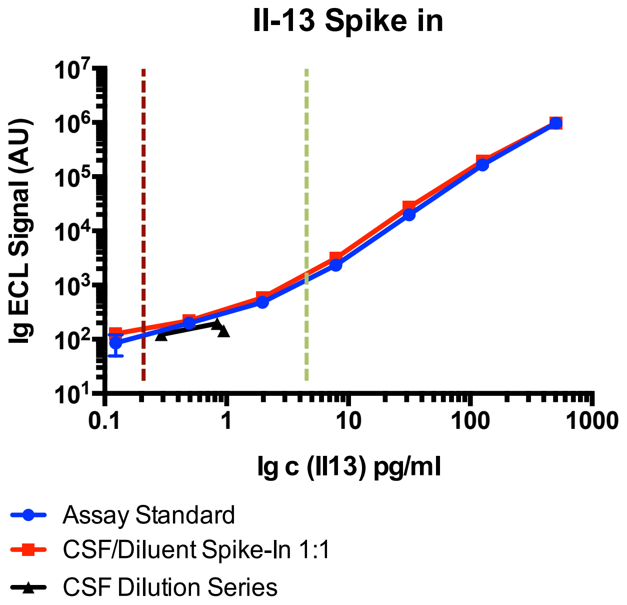

B) Recalculated concentrations curve

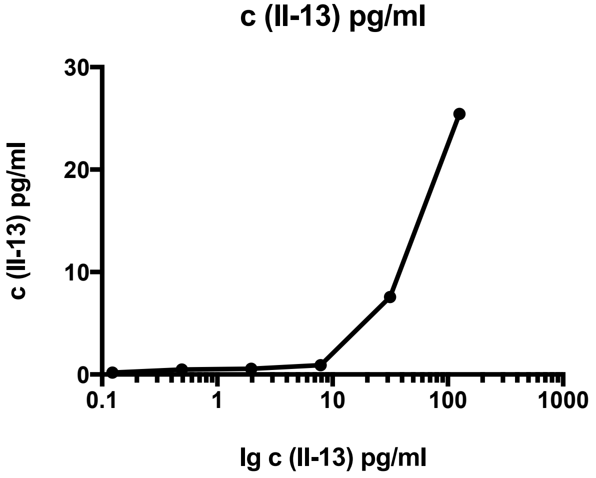

C) CSF dilution series

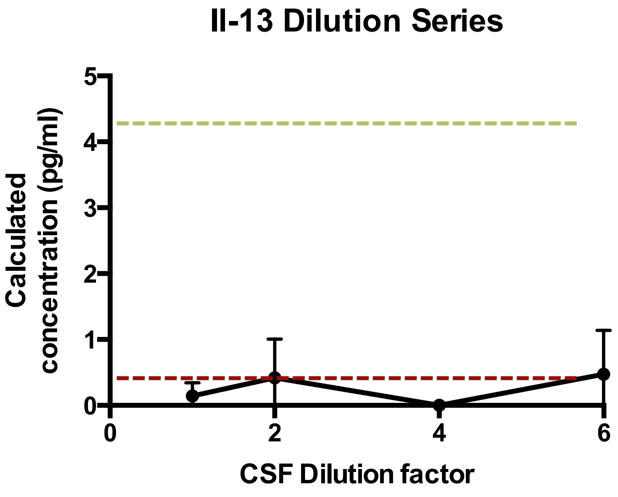

D) Test sample results

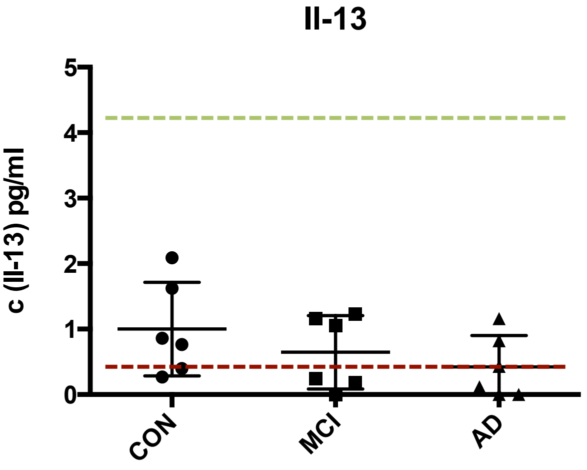

iv. Single analytes results

Il-15 (electrochemiluminescence multiplex; cytokine panel)

Evaluation: Borderline

Optimal dilution factor is 2x; spike-in setup might improve detectability

- Spike-In-Curve has positive difference to standard curve
- Spike in Concentrations are linear in range of standard 5 – 8 at 2.0 pg/ml (2x Dilution)
- Sample concentrations > LLOQ (1.4 pg/ml)
- Sample concentrations in linear range
- Conflicting QC:
    - One of 12 tested samples below LLOQ
    - One of 12 tested Samples has slightly elevated CV (23%)
- No significant difference between samples

A) Spike In Curve

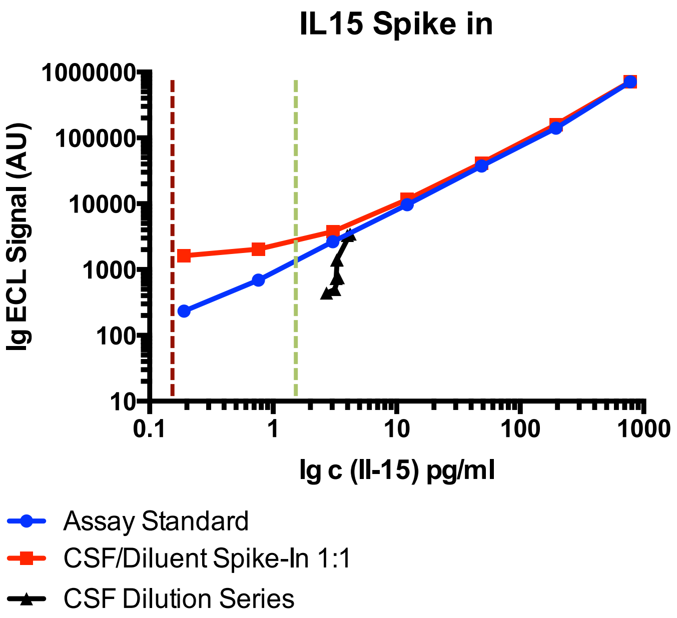

B) Recalculated concentrations curve

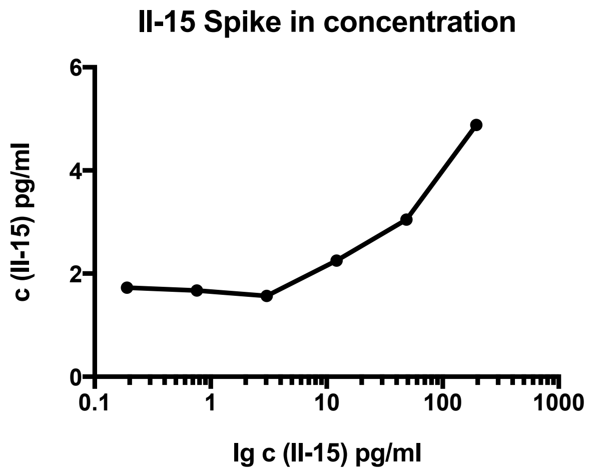

C) CSF dilution series

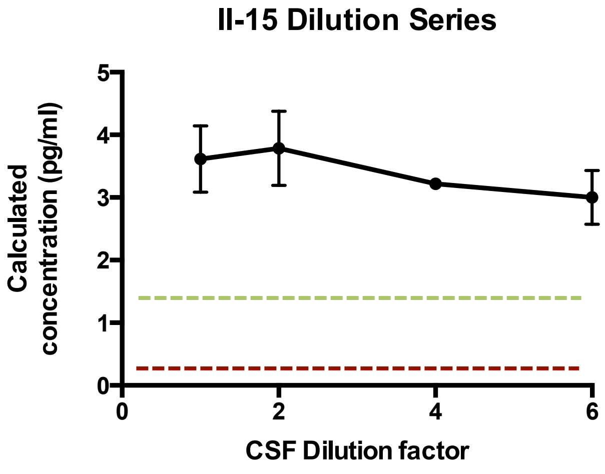

D) Test sample results

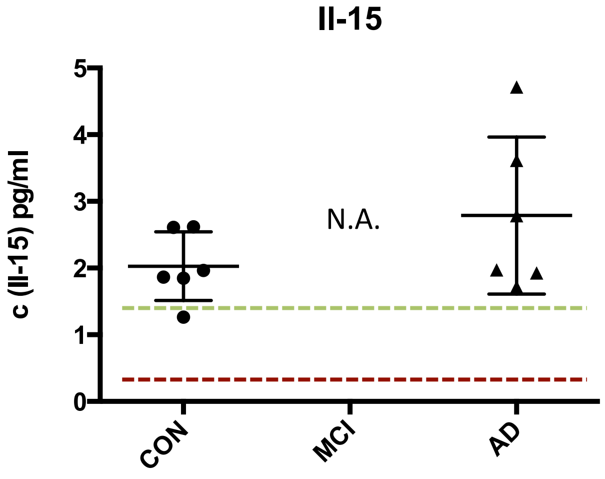

iv. Single analytes results

Il-16 (electrochemiluminescence multiplex; cytokine panel)

Evaluation: Borderline

Samples not detectable but might be drawn in detection range by spike-in of protein

- Spike-In-Curve overlaps with standard curve
- Spike in Concentrations are linear in range of standard 5 – 8 at 10.0 pg/ml (2x Dilution)
- Sample concentrations < LLOQ (19.1 pg/ml)
- No significant difference between test samples

A) Spike In Curve

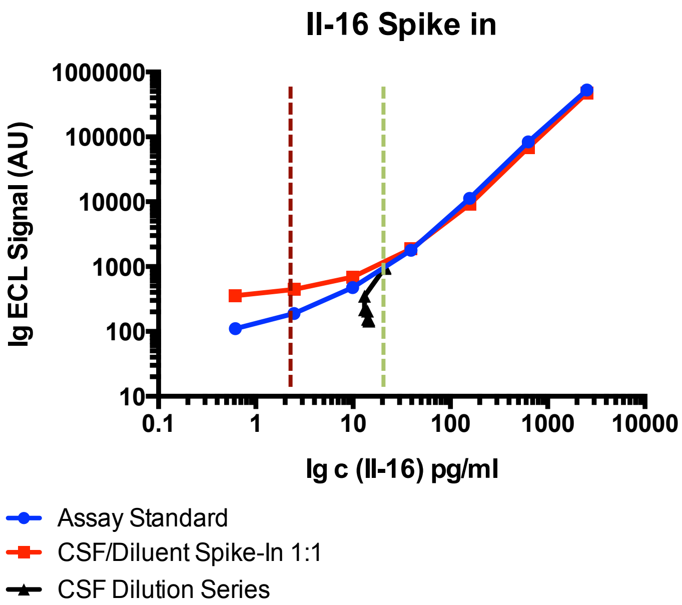

B) Recalculated concentrations curve

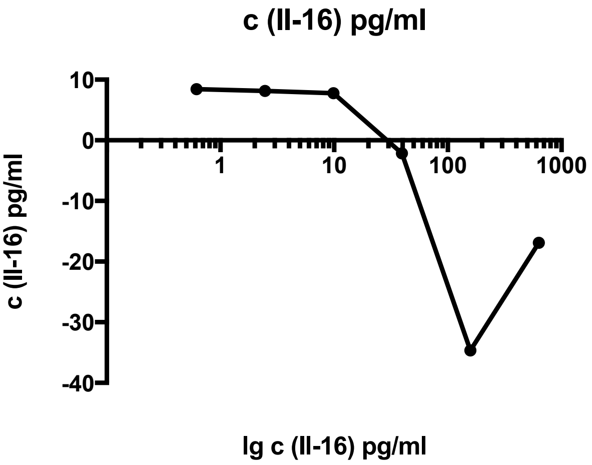

C) CSF dilution series

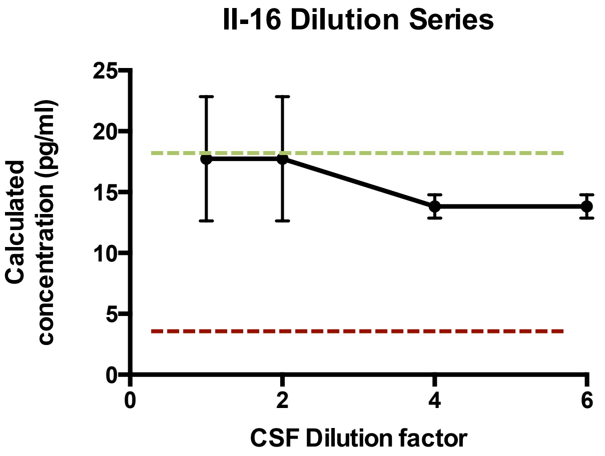

D) Test sample results

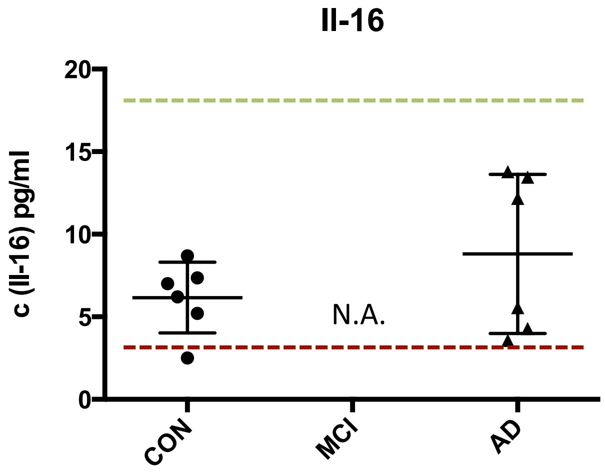

iv. Single analytes results

Il-17A (electrochemiluminescence multiplex; cytokine panel)

Evaluation: Not passed

Samples not detectable

- Spike-In-Curve overlaps with standard curve
- Spike in Concentrations are nonlinear
- Sample concentrations < LLOD (0.74 pg/ml)
- No significant difference between test samples

A) Spike In Curve

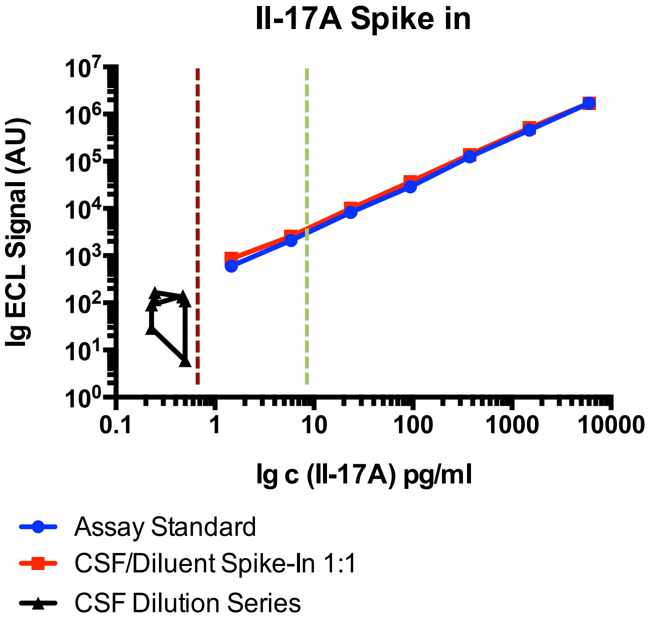

B) Recalculated concentrations curve

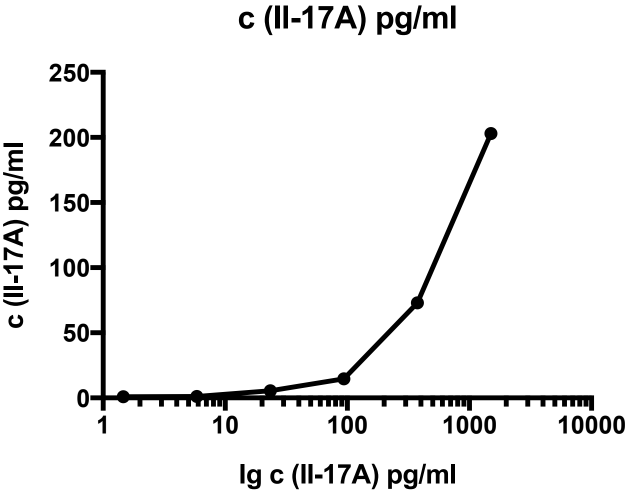

C) CSF dilution series

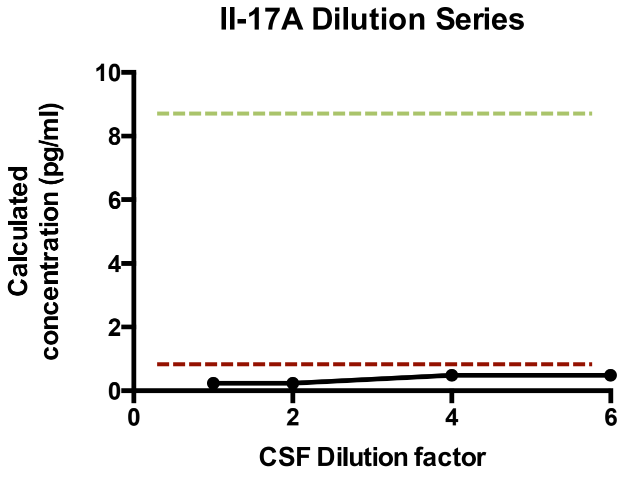

D) Test sample results

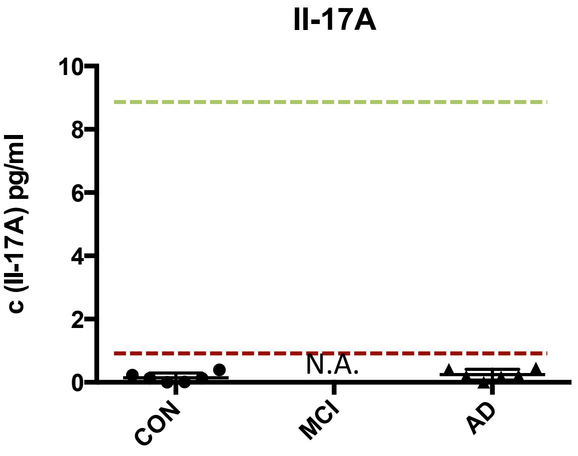

iv. Single analytes results

IP-10 (electrochemiluminescence multiplex; chemokine panel)

Evaluation: **Passed**

Optimal dilution factor is >6x

- Spike-In-Curve has positive difference to standard curve
- Spike in Concentrations are linear in range of standard 4 – 8 at 400 pg/ml (2x Dilution)
- Sample concentrations > LLOD (1.37 pg/ml)
- Sample concentrations in linear range if diluted
- No significant difference between test samples

A) Spike In Curve

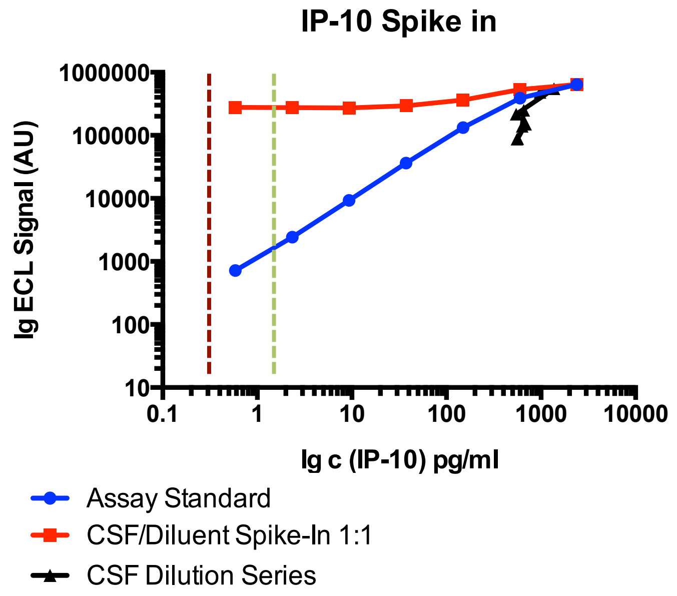

B) Recalculated concentrations curve

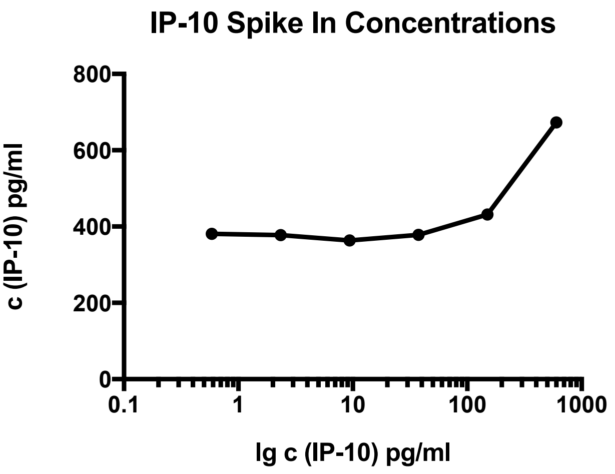

C) CSF dilution series

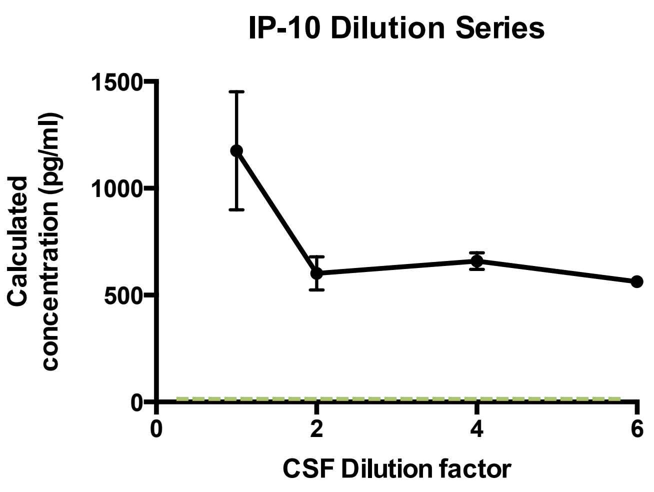

D) Test sample results

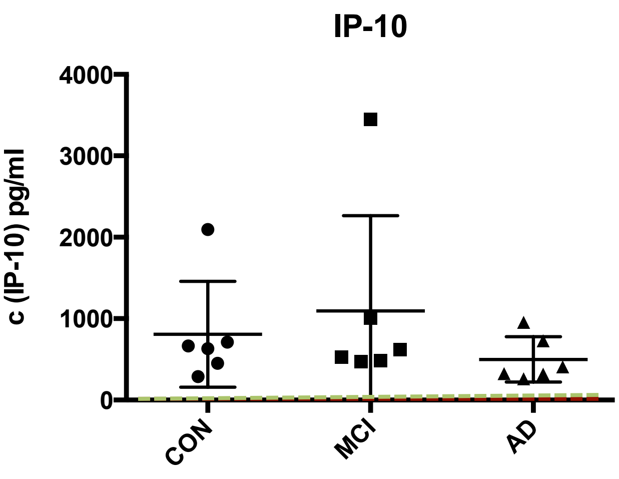

iv. Single analytes results

MCP-1 (electrochemiluminescence multiplex; chemokine panel)

Evaluation: Passed

Optimal dilution factor is >6x

- Spike-In-Curve has positive difference to standard curve
- Spike in Concentrations are linear in range of standard 3 – 8 at 250 pg/ml (2x Dilution)
- Sample concentrations > LLOD (1.09 pg/ml)
- Sample concentrations in linear range if diluted
- No significant difference between test samples

A) Spike In Curve

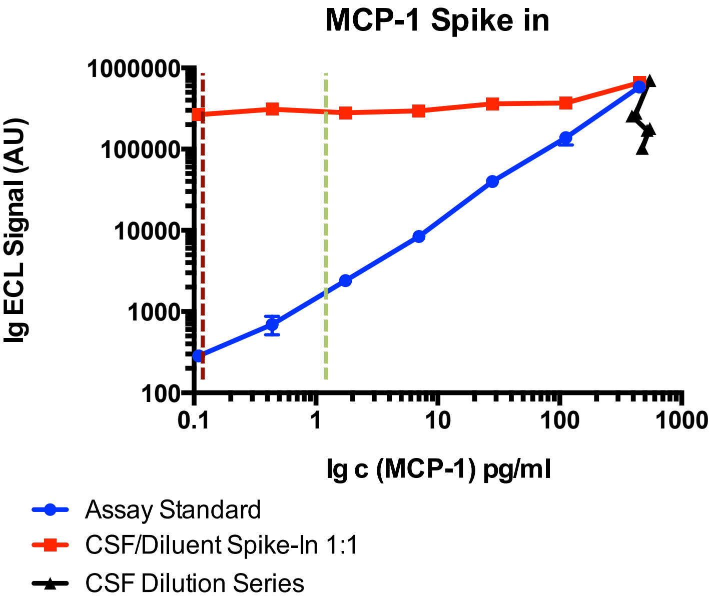

B) Recalculated concentrations curve

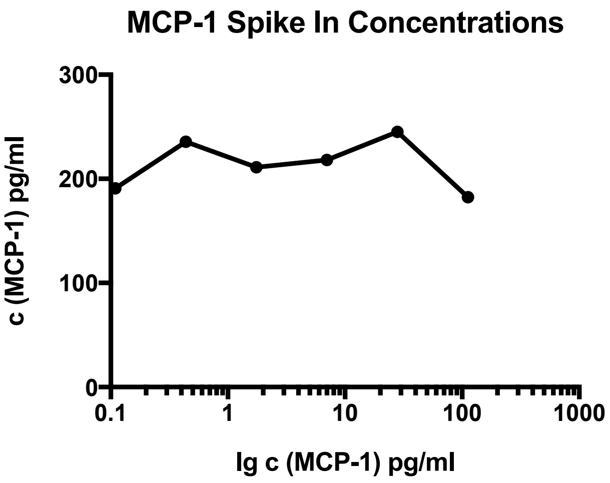

C) CSF dilution series

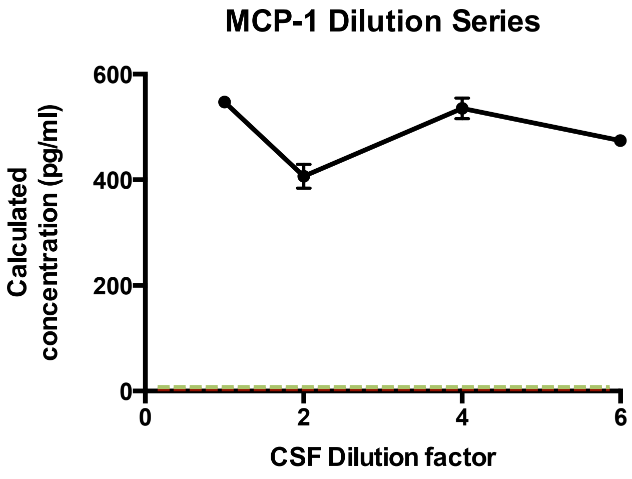

D) Test sample results

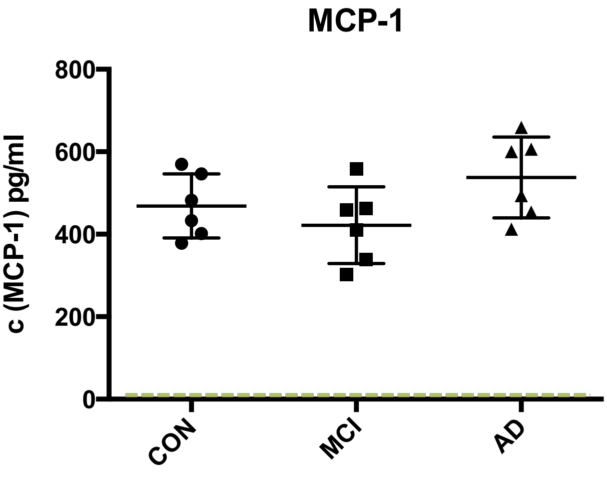

iv. Single analytes results

MCP-4 (electrochemiluminescence multiplex; chemokine panel)

Evaluation: Not passed

Samples not detectable

- Spike-In-Curve overlaps with standard curve
- Spike in Concentrations are nonlinear
- Sample concentrations < LLOQ (5.13 pg/ml)
- No significant difference between test samples

A) Spike In Curve

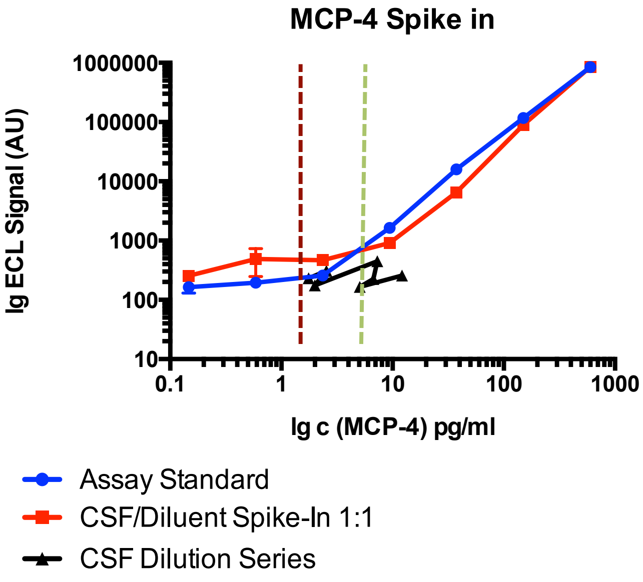

B) Recalculated concentrations curve

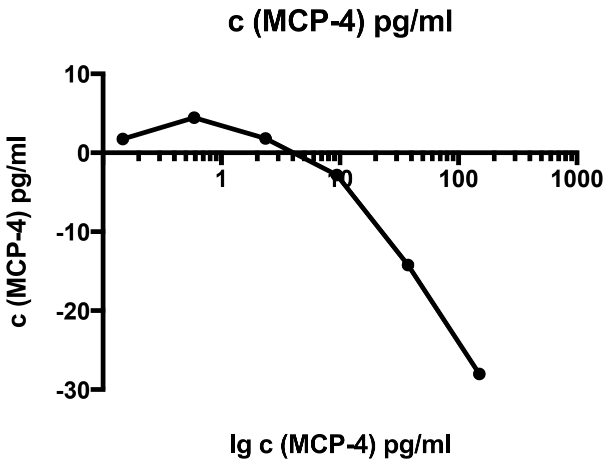

C) CSF dilution series

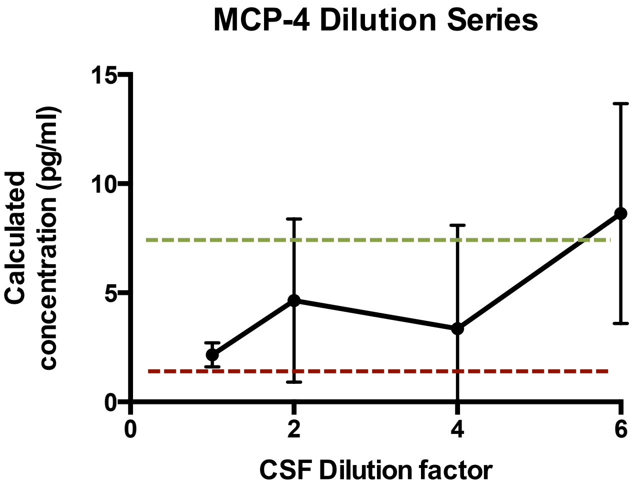

D) Test sample results

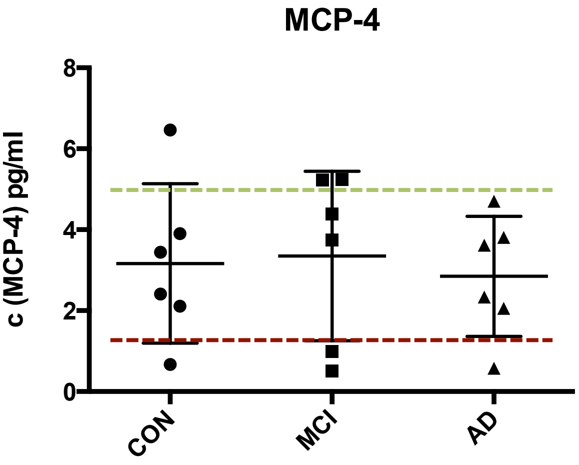

iv. Single analytes results

MDC (electrochemiluminescence multiplex; chemokine panel)

Evaluation: Borderline

Samples not detectable but might be drawn in detection range by spike-in of protein

- Spike-In-Curve has positive difference to standard curve
- Spike-In concentrations are linear only in the range of standard 6-8 at 20 pg/ml
- Sample concentrations < LLOQ (88.3 pg/ml)
- No significant difference between test samples

A) Spike In Curve

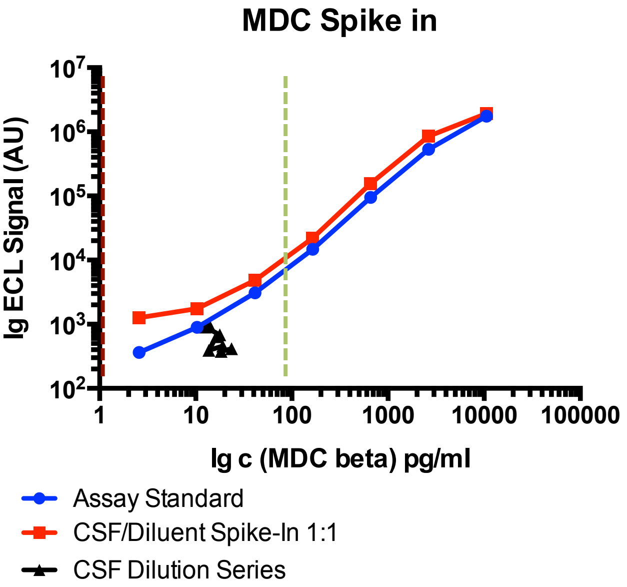

B) Recalculated concentrations curve

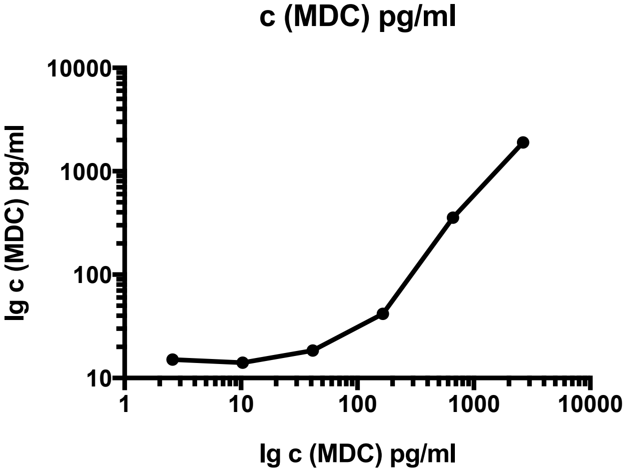

C) CSF dilution series

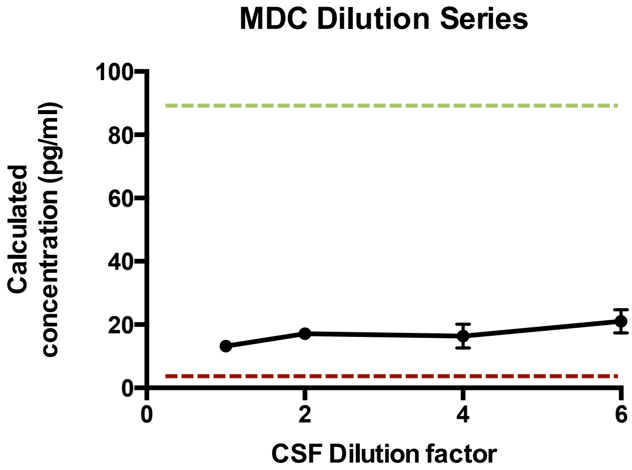

D) Test sample results

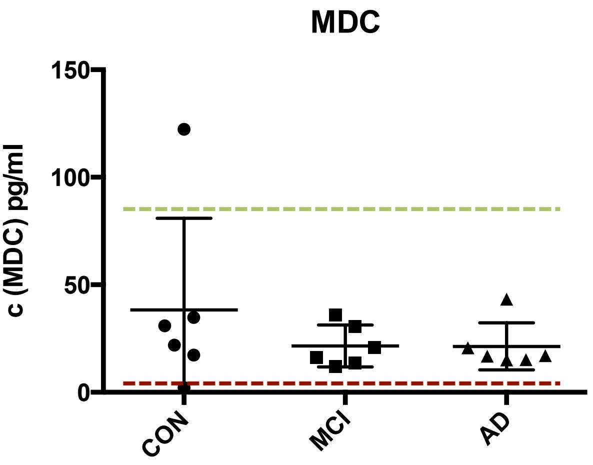

iv. Single analytes results

MIF (electrochemiluminescence singleplex)

Evaluation: Passed

Optimal dilution factor is 5x

- Spike-In-Curve has positive difference to standard curve
- Spike in Concentrations are linear in range of standard 4 – 8 at 1000 pg/ml (2x Dilution)
- Sample concentrations > LLOD (2.4 pg/ml)
- Sample concentrations in linear range
- All QC parameters passed
- No significant difference between test samples

A) Spike In Curve

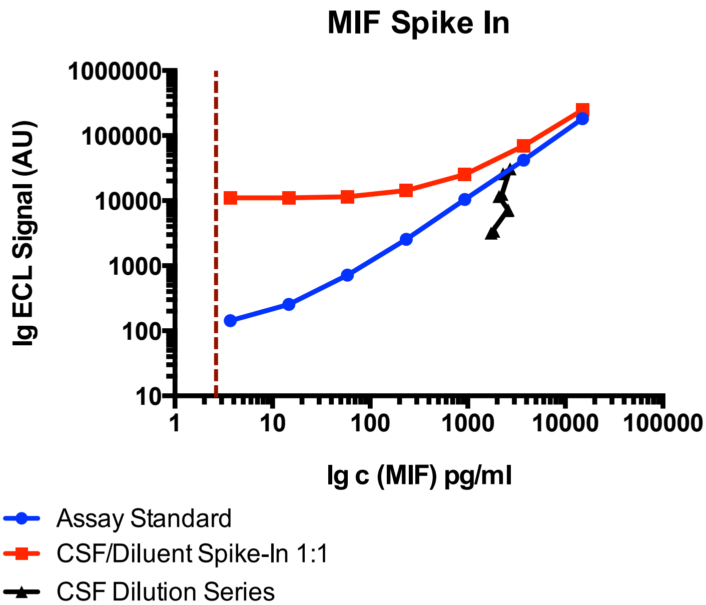

B) Recalculated concentrations curve

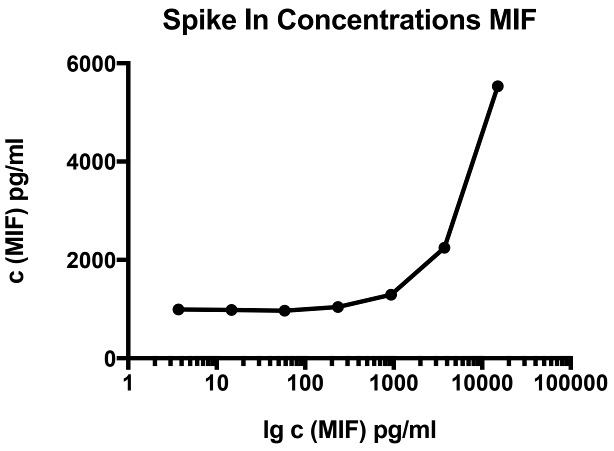

C) CSF dilution series

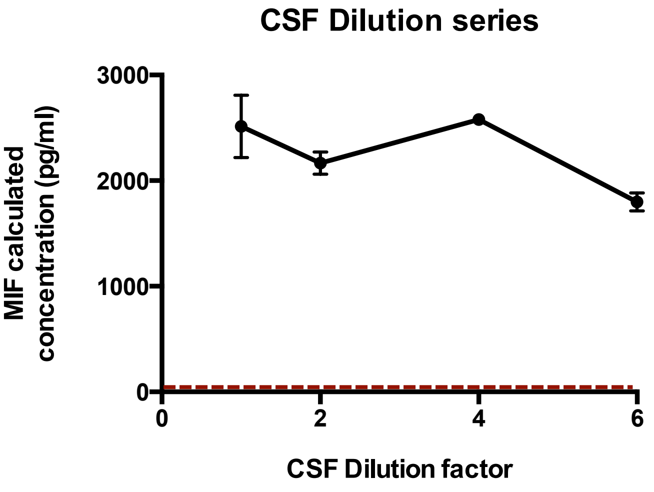

D) Test sample results

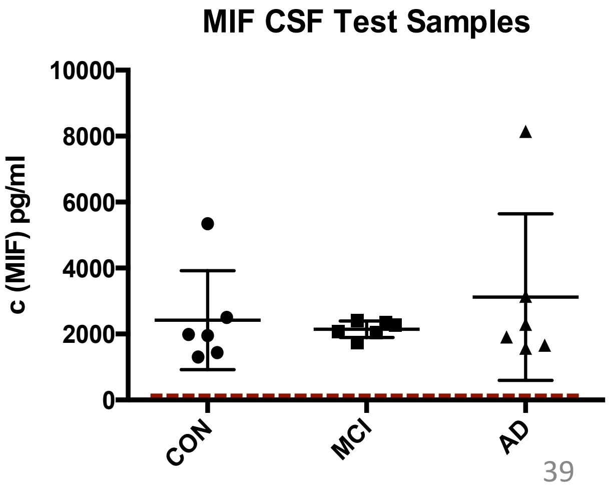

iv. Single analytes results

MIP-1α (electrochemiluminescence multiplex; chemokine panel)

Evaluation: Not passed

Samples not detectable

- Spike-In-Curve has positive difference to standard curve
- Spike in Concentrations are nonlinear
- Sample concentrations < LLOQ (13.8 pg/ml)
- No significant difference between test samples

A) Spike In Curve

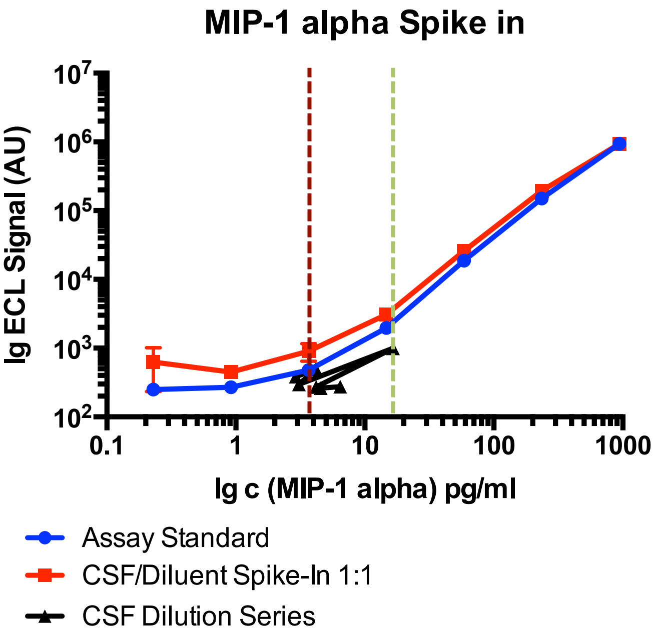

B) Recalculated concentrations curve

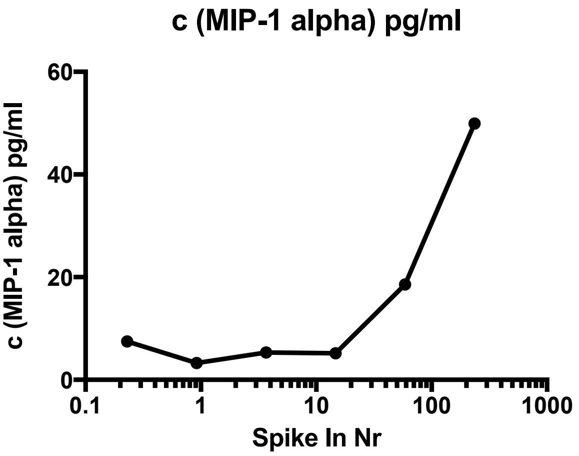

C) CSF dilution series

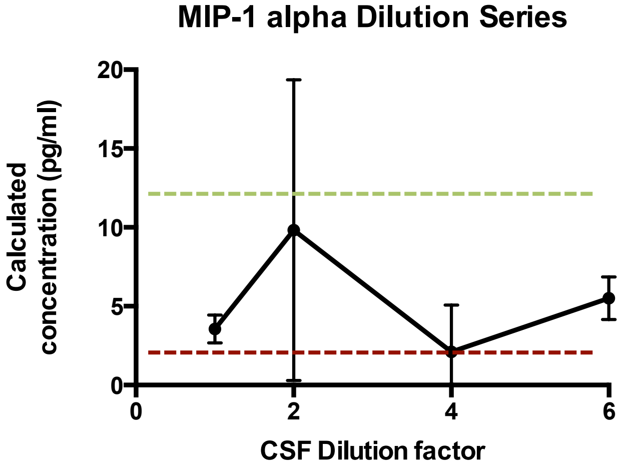

D) Test sample results

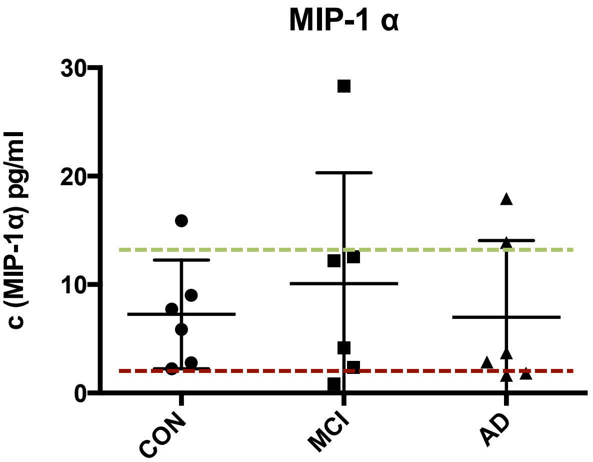

iv. Single analytes results

MIP-1β (electrochemiluminescence multiplex; chemokine panel)

Evaluation: Passed (close to borderline)

Optimal dilution factor is 2x; spike-in setup would probably improve detectability

- Spike-In-Curve has positive difference to standard curve
- Spike in Concentrations are linear in range of standard 4 – 7 at 5.0 pg/ml (2x Dilution)
- Sample concentrations > LLOD (2.27 pg/ml)
- Sample concentrations in linear range
- No significant difference between test samples

A) Spike In Curve

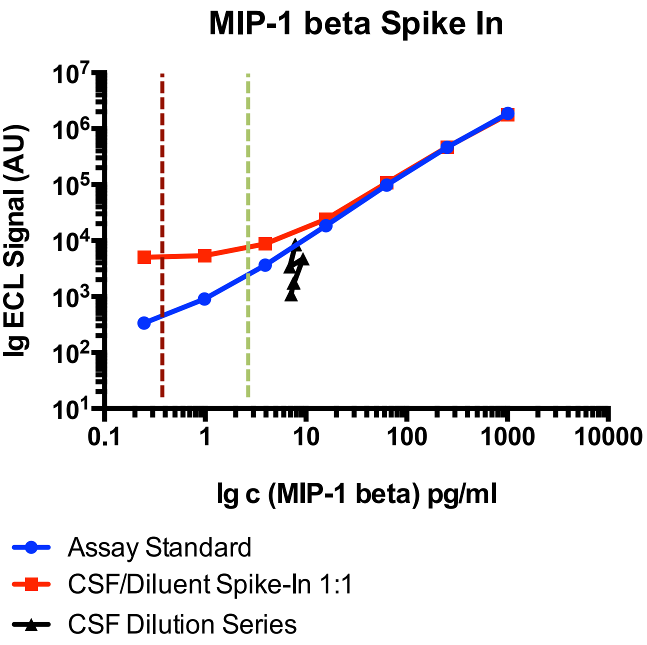

B) Recalculated concentrations curve

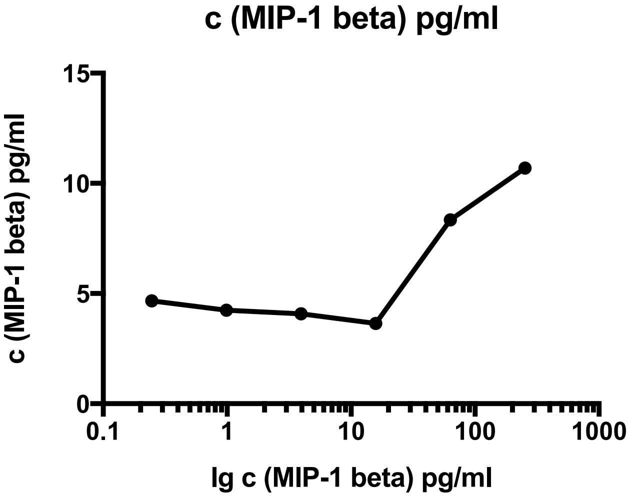

C) CSF dilution series

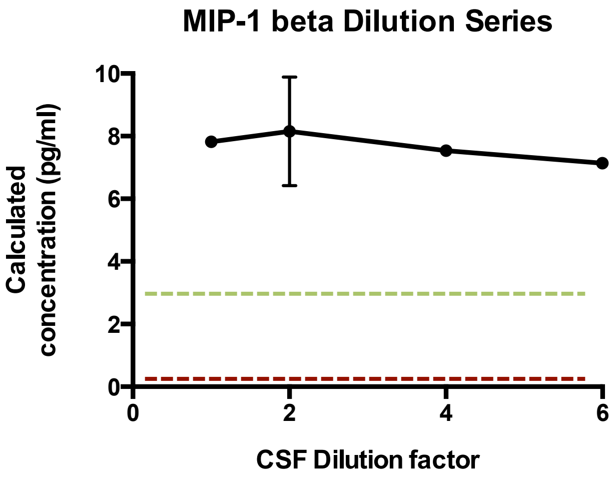

D) Test sample results

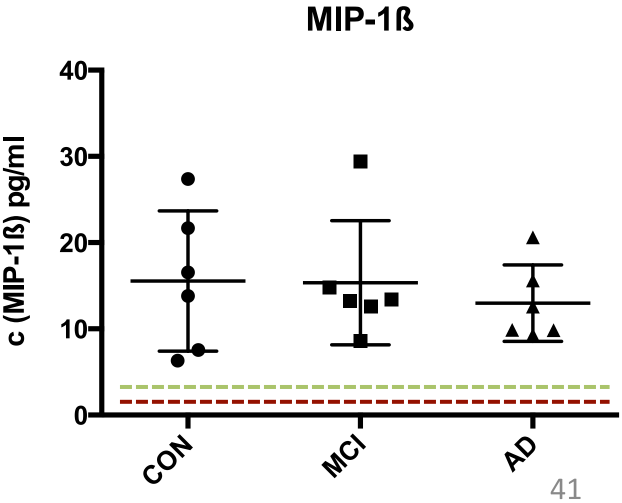

iv. Single analytes results

Mrp8/14 (colorimetric singleplex)

Evaluation: Not passed

Inconsistent CSF data

- Spike-In-Curve overlaps with standard curve (standard curve was extended in low range)
- Spike in Concentrations are nonlinear and equal to zero
- CSF signal low even in extended assay range
- For this analyte, data from a previous pilot study with larger numbers was used. Yet, 50% of samples were undetectable due to low CSF levels
- No significant difference between detectable test samples

A) Spike In Curve

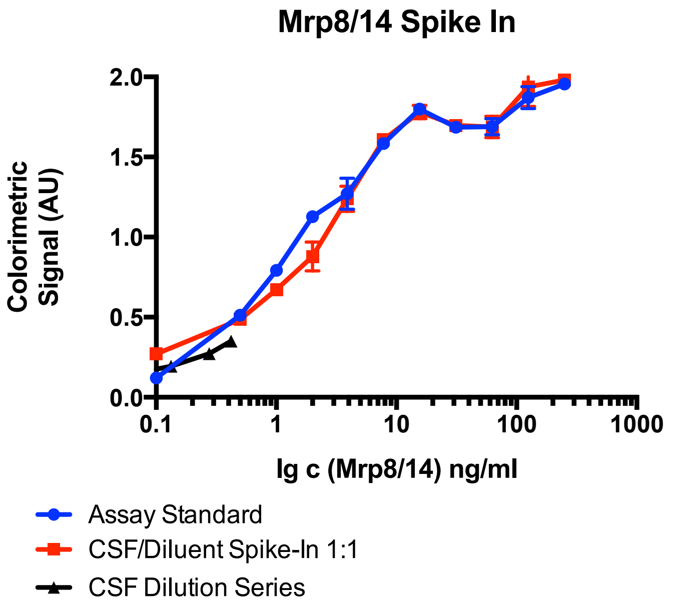

B) Recalculated concentrations curve

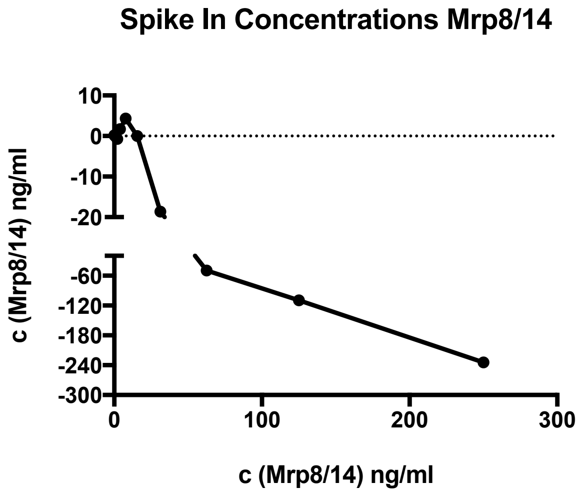

C) CSF dilution series

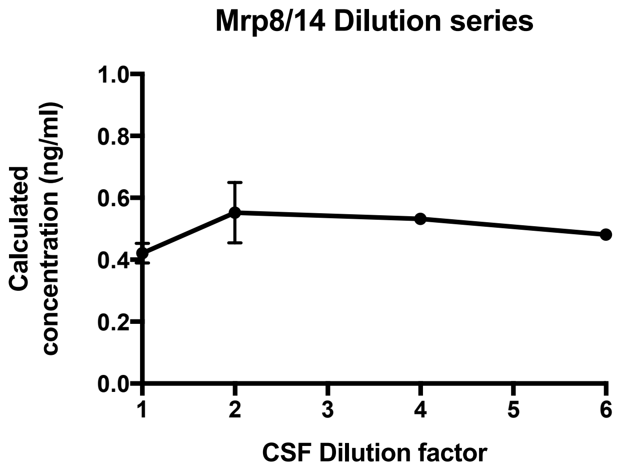

D) Test sample results

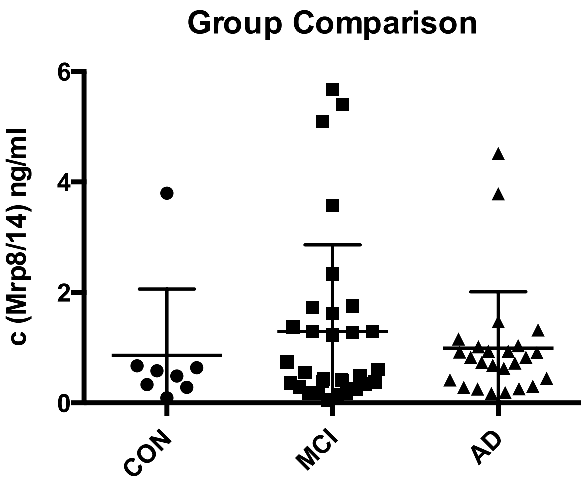

iv. Single analytes results

PIGF (electrochemiluminescence multiplex; angiogenesis panel)

Evaluation: Passed

Optimal dilution factor is 2-4x

- Spike-In-Curve has positive difference to standard curve
- Spike in Concentrations are linear in range of standard 4 – 8 at 50 pg/ml (2x Dilution)
- Sample concentrations > LLOQ (10.7 pg/ml)
- Sample concentrations in linear range
- No significant difference between test samples

A) Spike In Curve

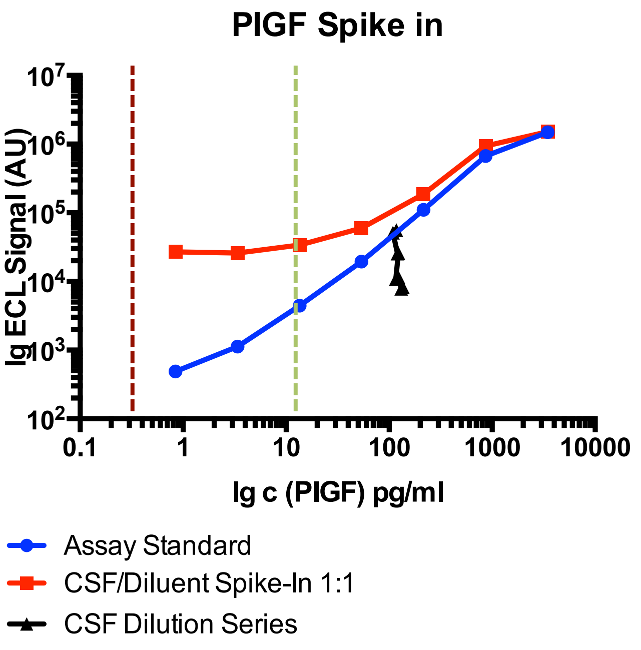

B) Recalculated concentrations curve

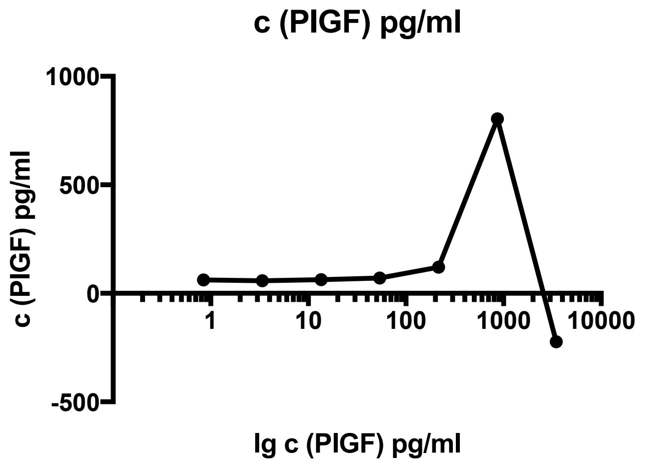

C) CSF dilution series

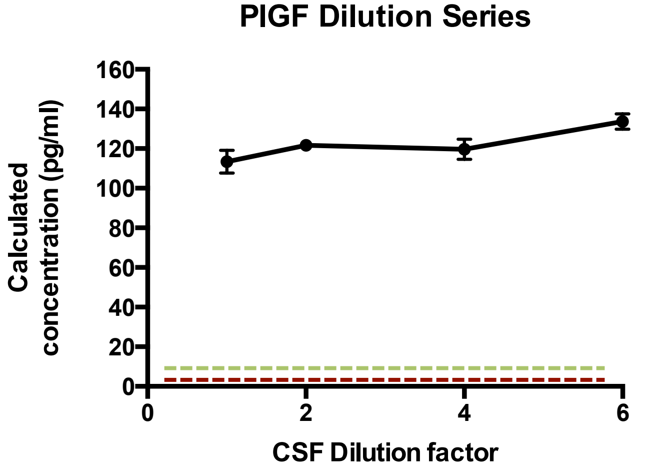

D) Test sample results

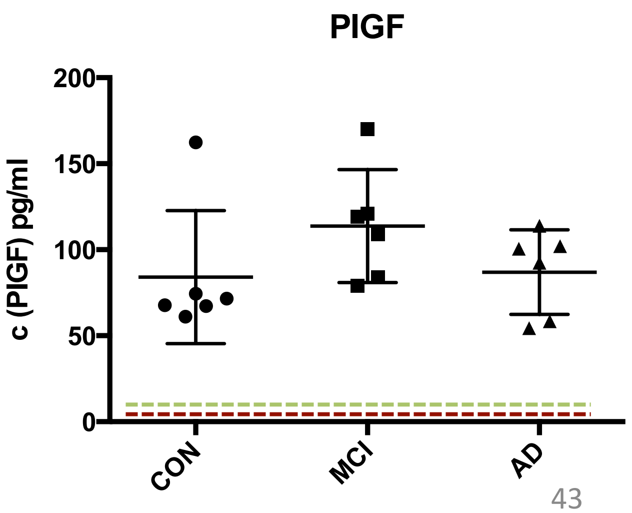

iv. Single analytes results

SAA (electrochemiluminescence multiplex; vascular injury panel)

Evaluation: Passed

Optimal dilution factor is 4-6x

- Spike-In-Curve has positive difference to standard curve
- Spike in Concentrations are linear in range of standard 3 – 8 at 20.000 pg/ml (2x Dilution)
- Sample concentrations > LLOQ (54.1 pg/ml)
- Sample concentrations in linear range
- All QC parameters passed
- No significant difference between test samples

A) Spike In Curve

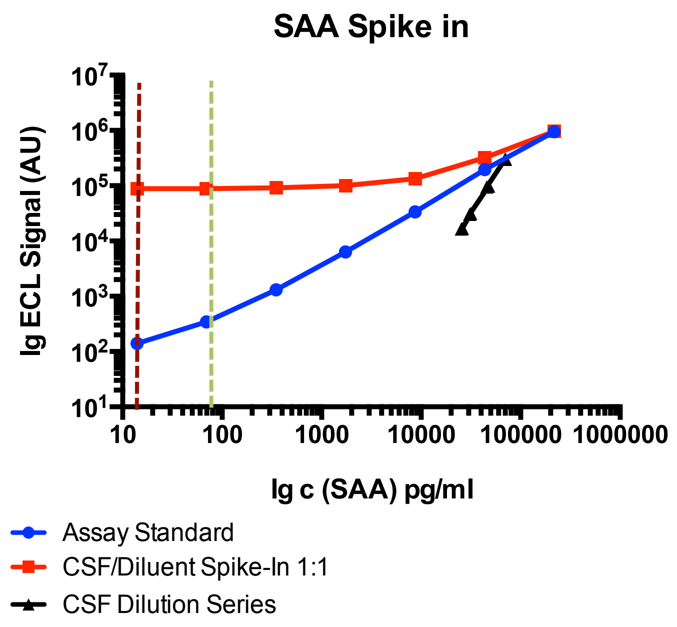

B) Recalculated concentrations curve

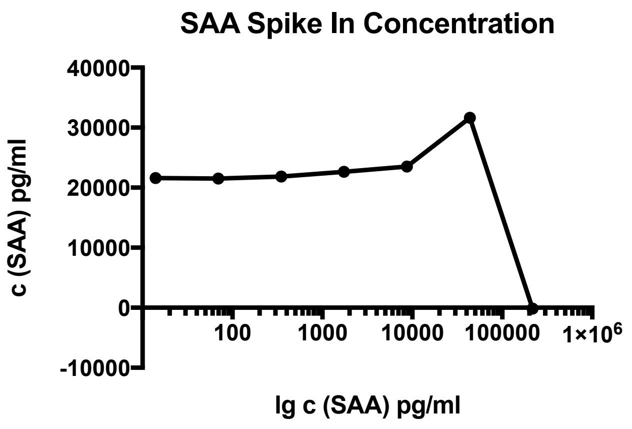

C) CSF dilution series

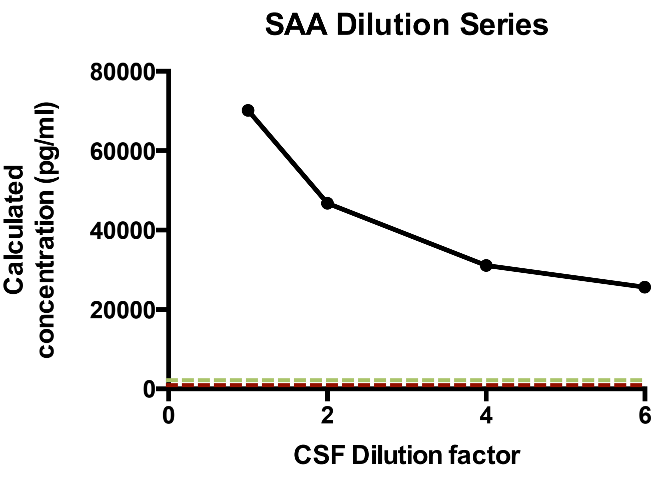

D) Test sample results

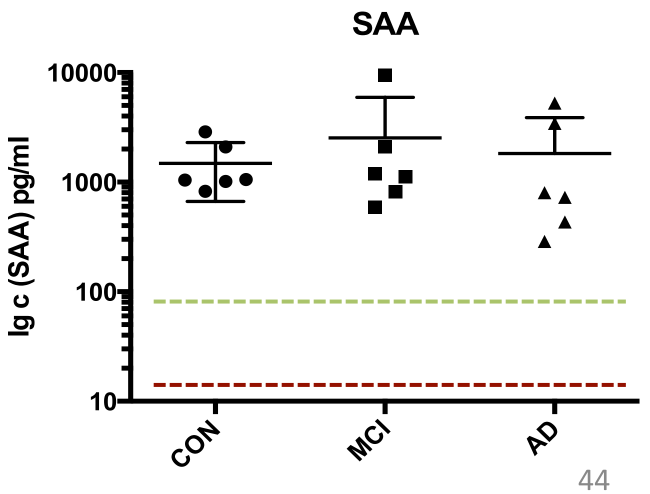

iv. Single analytes results

TARC (electrochemiluminescence multiplex; chemokine panel)

Evaluation: **Borderline**

Samples not detectable but might be drawn in detection range by spike-in of protein

- Spike-In-Curve has positive difference to standard curve
- Spike in Concentrations are linear in range of standard 4 – 8 at 4.0 pg/ml (2x Dilution)
- Sample concentrations overlap with LLOQ (3.32 pg/ml)
- Sample concentrations at borderline of quantitation
- No significant difference between test samples

A) Spike In Curve

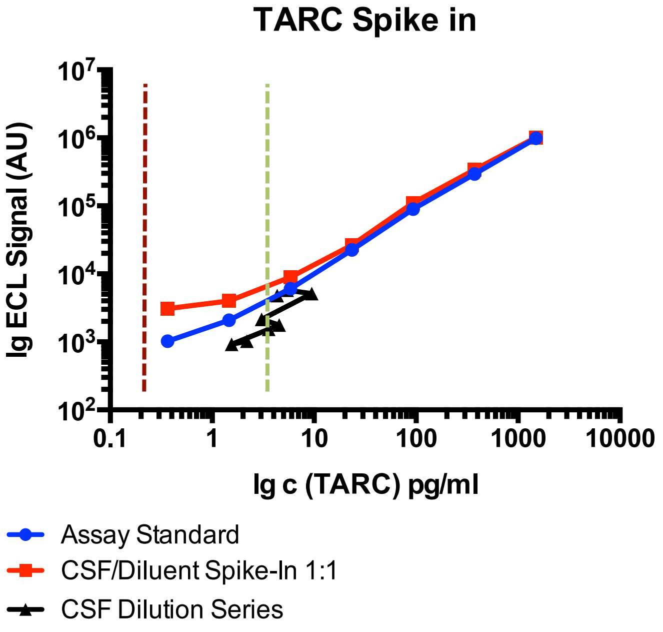

B) Recalculated concentrations curve

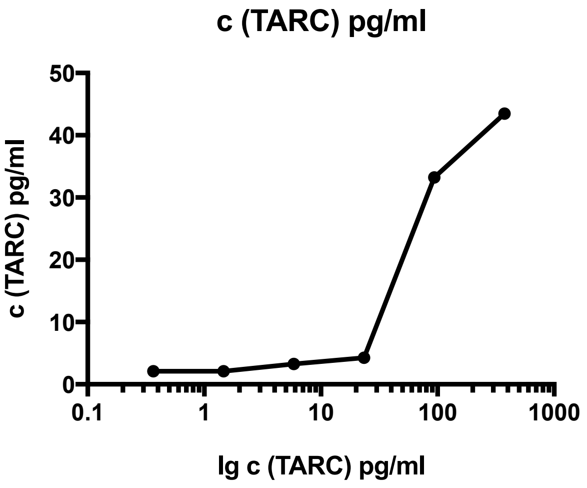

C) CSF dilution series

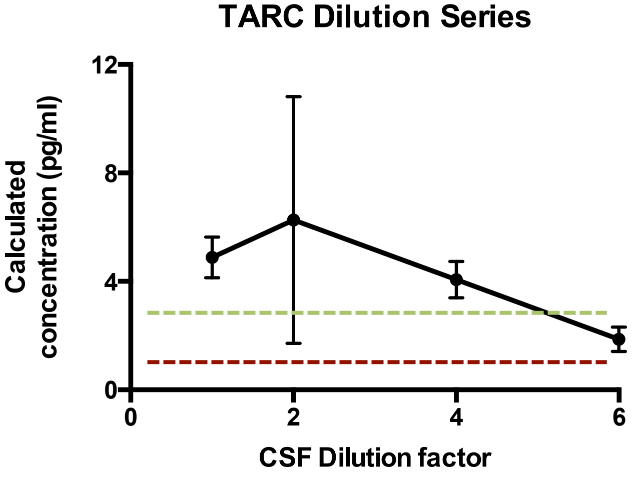

D) Test sample results

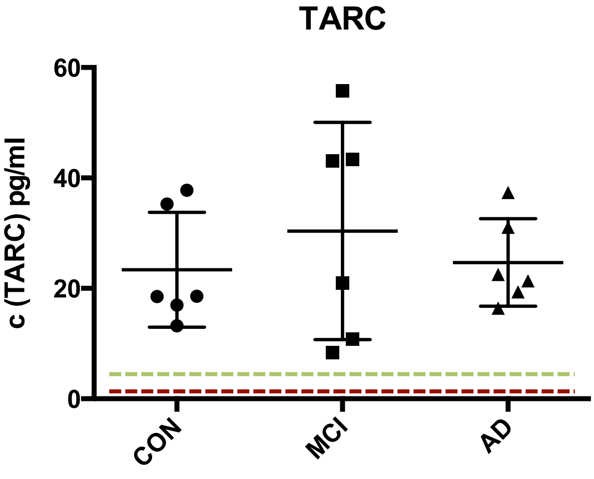

iv. Single analytes results

Tie-2 (electrochemiluminescence multiplex; angiogenesis panel)

Evaluation: Not passed

Samples not detectable

- Spike-In-Curve has overlaps with standard curve
- Spike-In concentrations are close to 0 in range of standard 4 - 8
- Sample concentrations < LLOQ (396.0 pg/ml)
- No significant difference between test samples

A) Spike In Curve

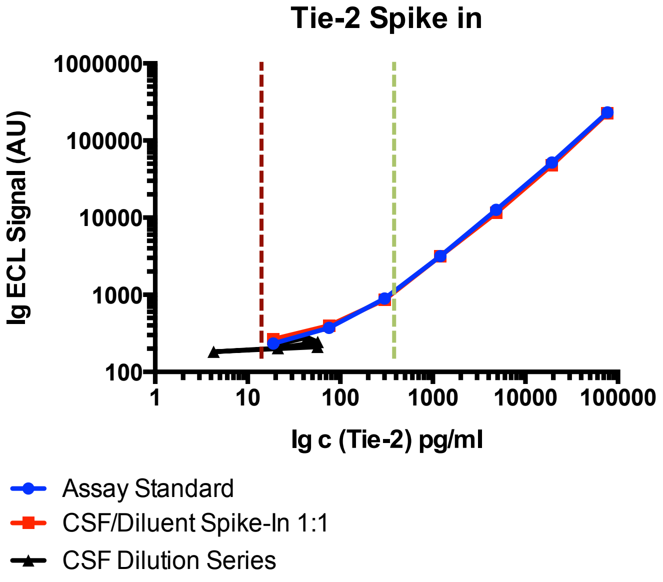

B) Recalculated concentrations curve

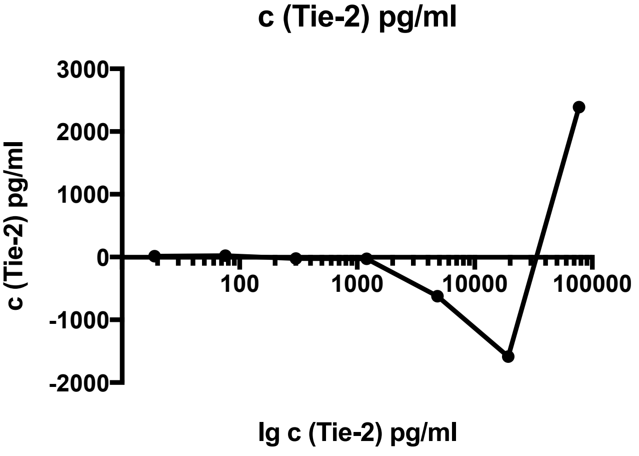

C) CSF dilution series

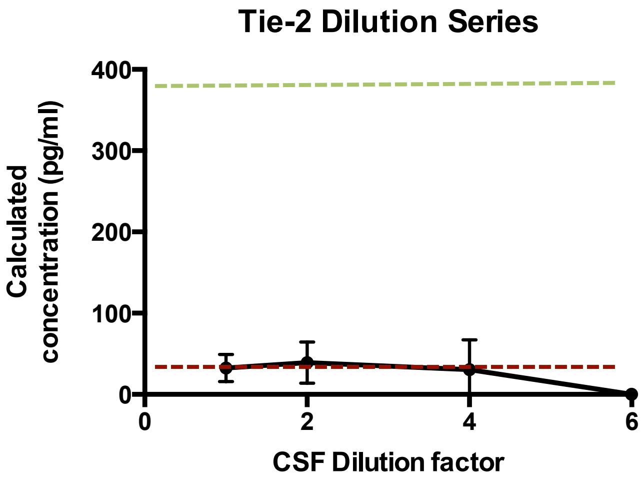

D) Test sample results

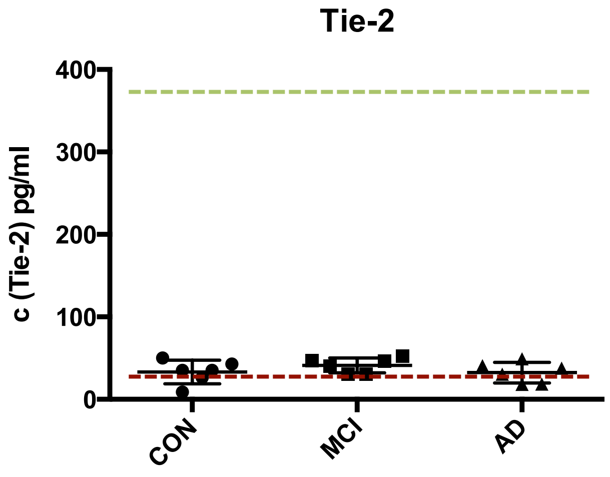

iv. Single analytes results

TNF-α (electrochemiluminescence multiplex; proinflammatory panel)

Evaluation: Not passed

Samples not detectable

- Spike-In-Curve overlaps with standard curve
- Spike in Concentrations are nonlinear
- Sample concentrations < LLOQ (0.69 pg/ml)
- No significant difference between test samples

A) Spike In Curve

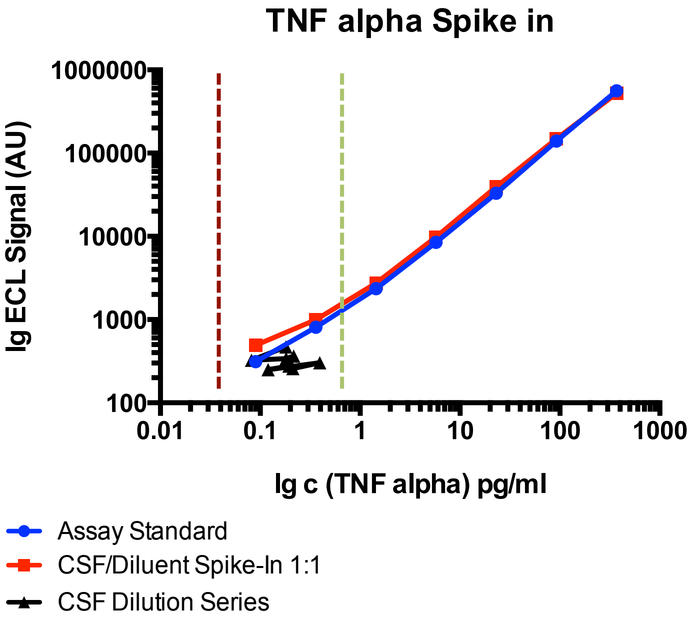

B) Recalculated concentrations curve

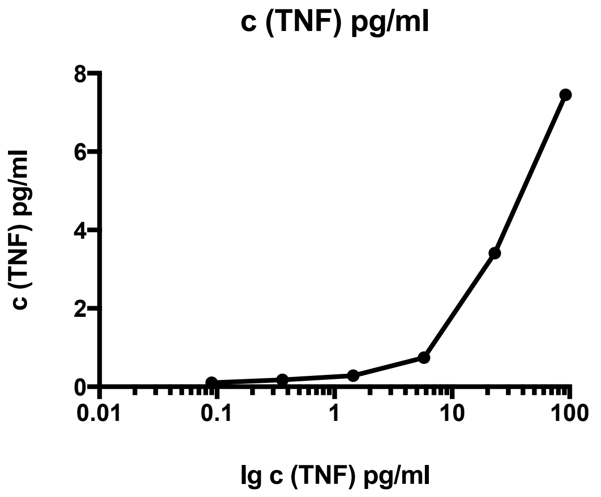

C) CSF dilution series

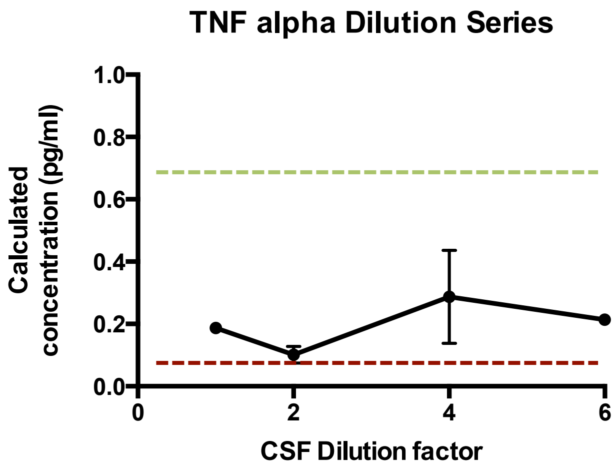

D) Test sample results

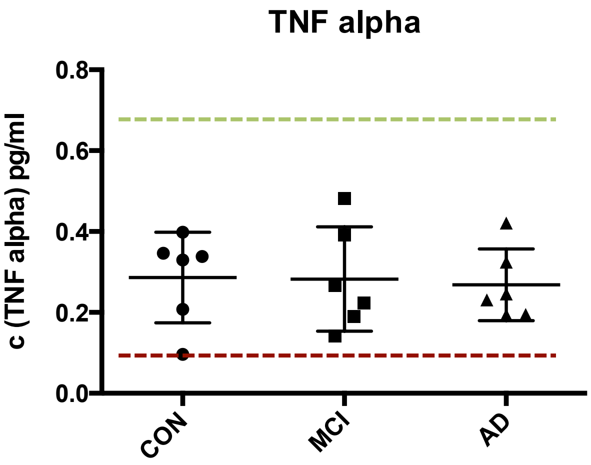

iv. Single analytes results

TNF-β (electrochemiluminescence multiplex; cytokine panel)

Evaluation: Not passed

Samples not detectable

- Spike-In-Curve overlaps with standard curve
- Spike in Concentrations are nonlinear
- Sample concentrations < LLOQ (1.15 pg/ml)
- No significant differences between test samples

A) Spike In Curve

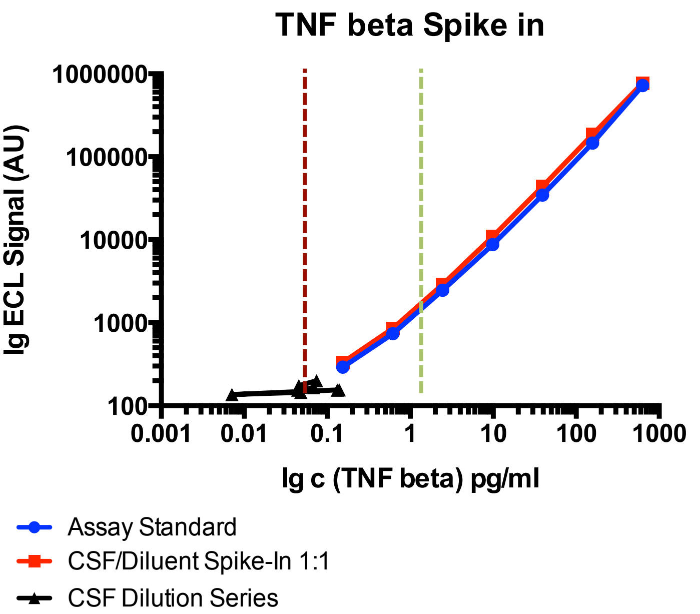

B) Recalculated concentrations curve

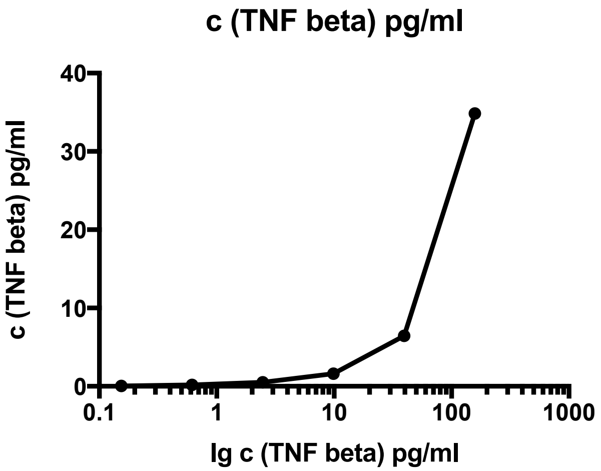

C) CSF dilution series

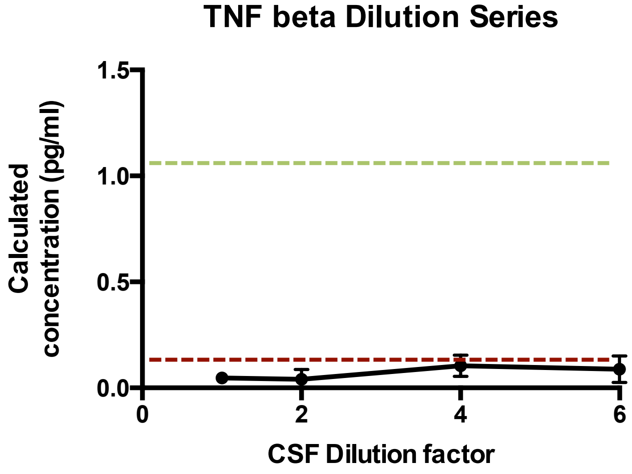

D) Test sample results

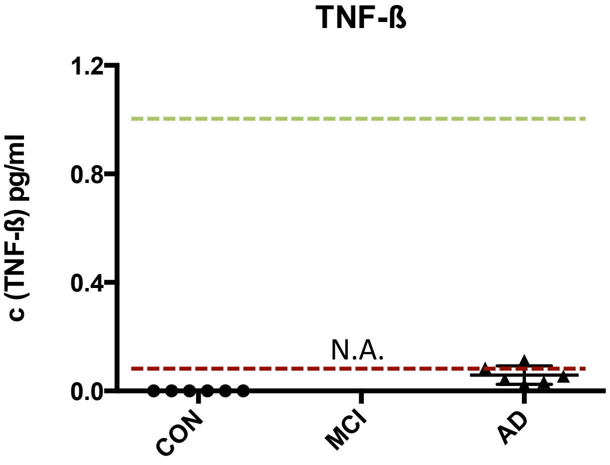

iv. Single analytes results

TREM2 (electrochemiluminescence singleplex)

Evaluation: Passed

Optimal dilution factor is 5x

- Spike-In-Curve has positive difference to standard curve
- Spike in Concentrations are in linear in range of standard 4 – 8 at 12.000 pg/ml (5x Dilution)
- Sample concentrations in linear range
- All QC parameters passed
- No significant difference between test samples

A) Spike In Curve

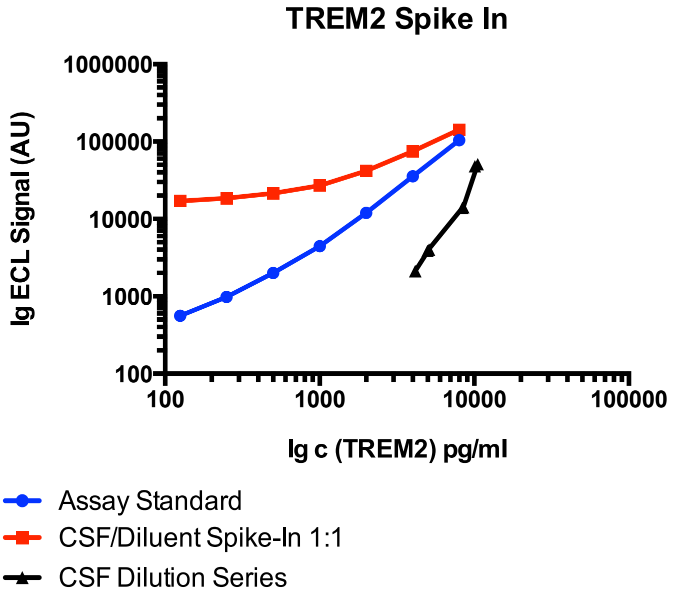

B) Recalculated concentrations curve

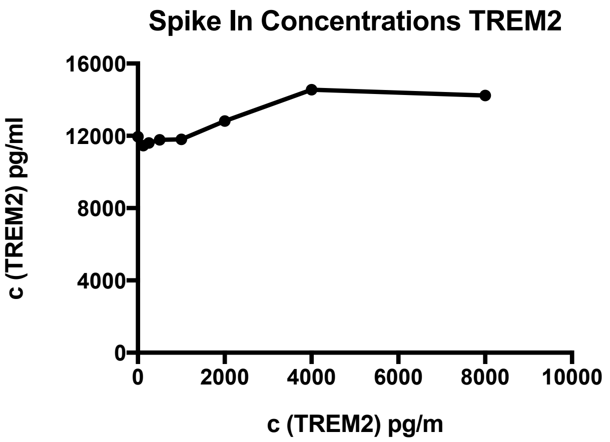

C) CSF dilution series

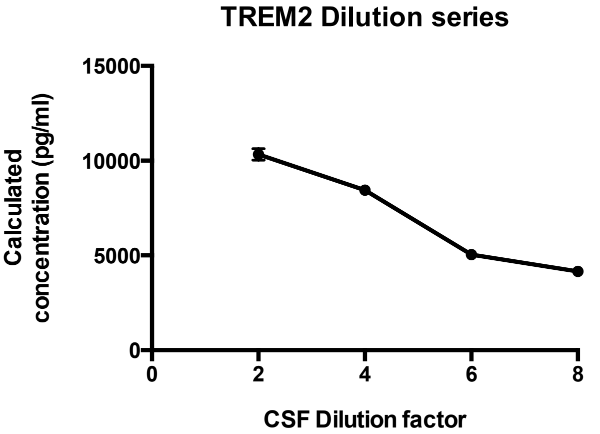

D) Test sample results

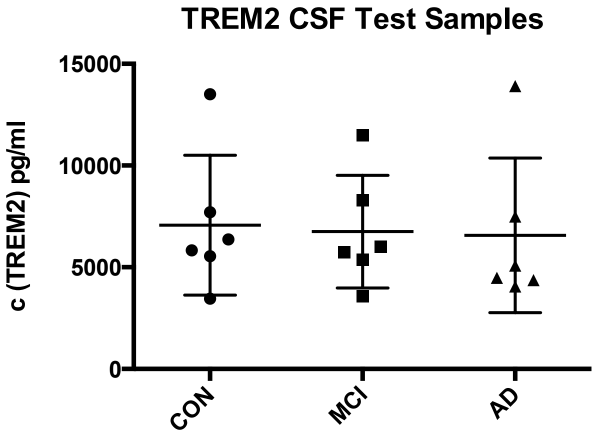

iv. Single analytes results

VCAM-1 (electrochemiluminescence multiplex; vascular Injury Panel)

Evaluation: Passed

Optimal dilution factor is >6x

- Spike-In-Curve has positive difference to standard curve
- Spike in Concentrations are linear in range of standard 2 – 8 at 5000 pg/ml (2x Dilution)
- Sample concentrations > LLOQ (37.6 pg/ml)
- Sample concentrations in linear range
- All QC parameters passed
- No significant difference between test samples

A) Spike In Curve

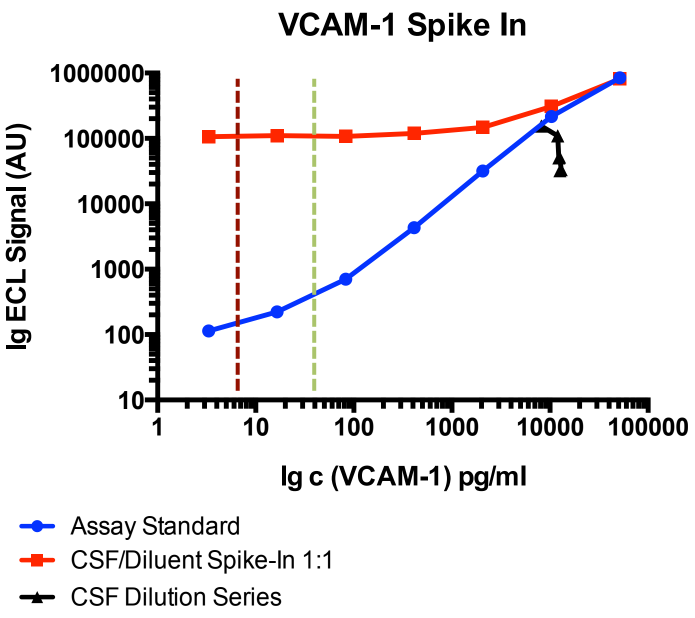

B) Recalculated concentrations curve

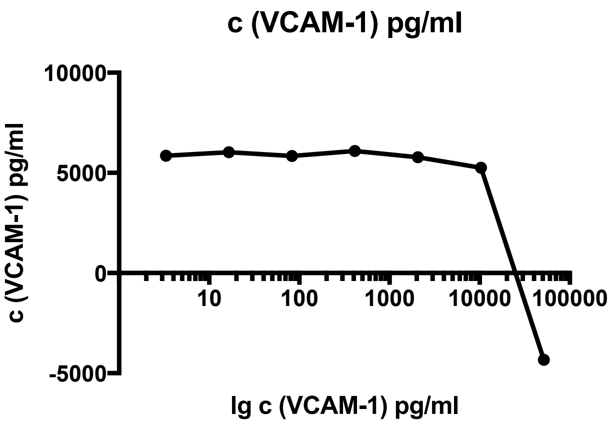

C) CSF dilution series

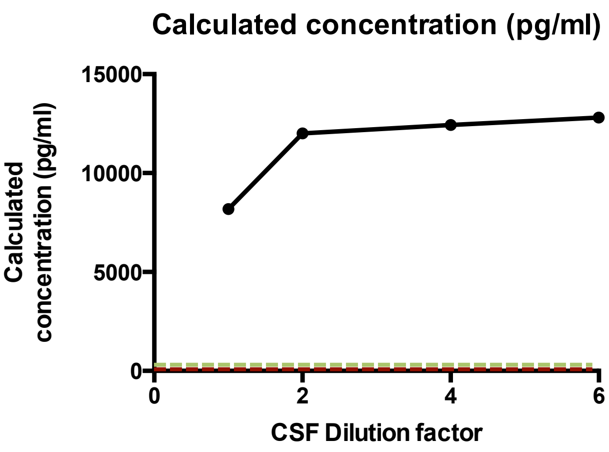

D) Test sample results

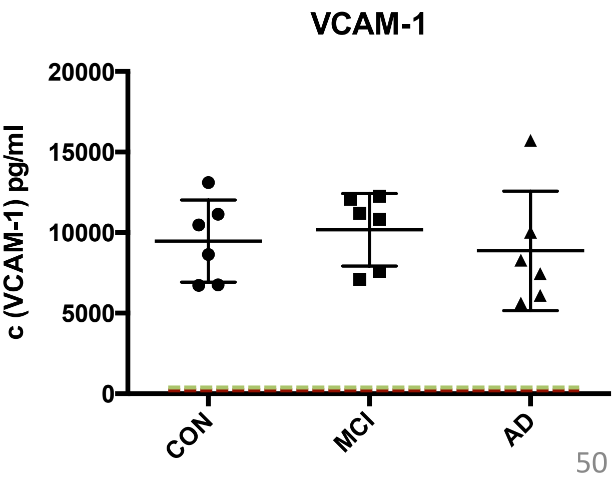

iv. Single analytes results

VEGF (electrochemiluminescence multiplex; cytokine panel)

Note: This assay was also tested as part of the angiogenesis panel (p. 52) were it was classified as borderline
Evaluation: Not passed

Samples not detectable

- Spike-In-Curve overlaps with standard curve
- Spike in Concentrations are nonlinear
- Sample concentrations < LLOQ (7.7 pg/ml)
- No significant difference between test samples (p = 0.06 in nonparametric testing)

A) Spike In Curve

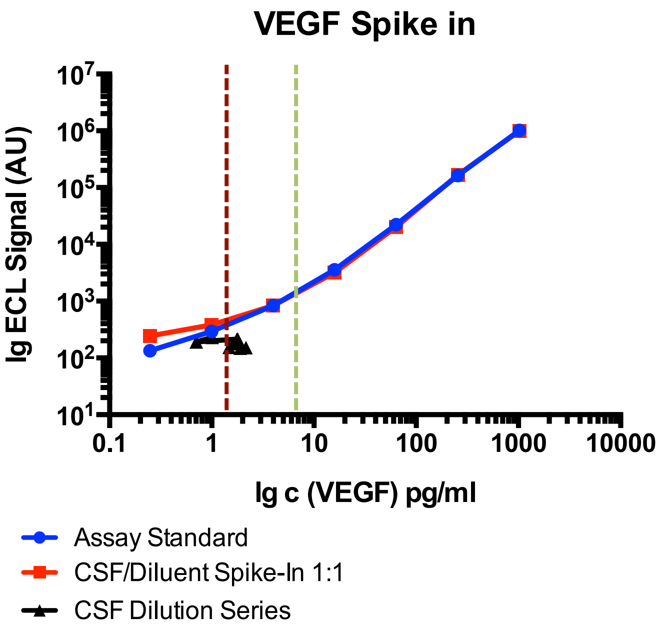

B) Recalculated concentrations curve

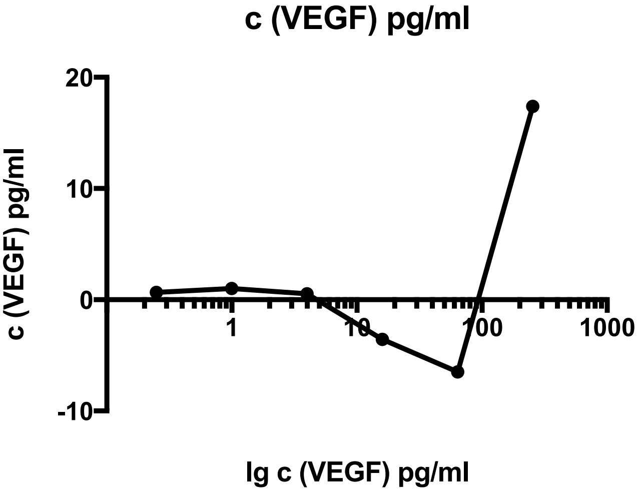

C) CSF dilution series

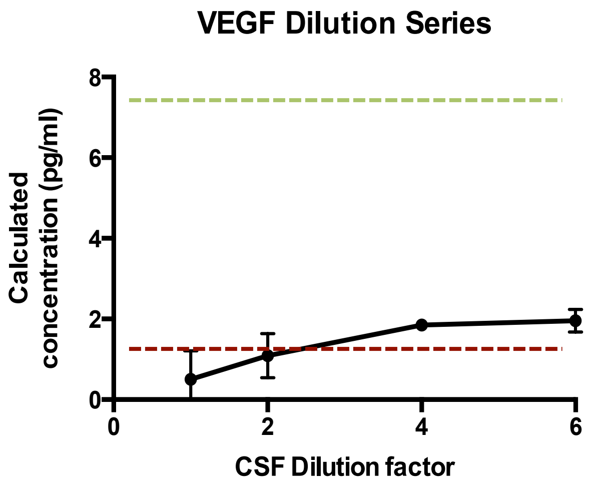

D) Test sample results

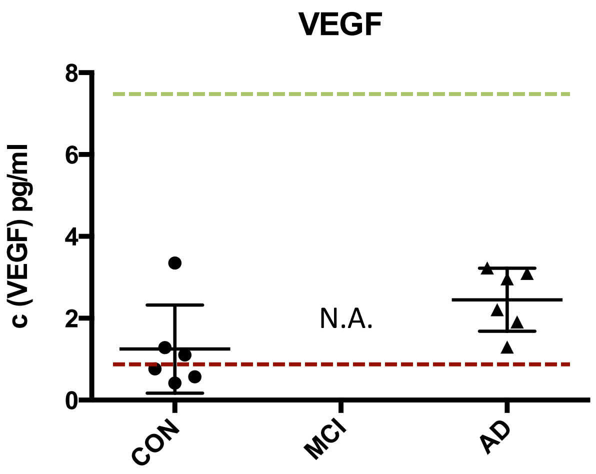

iv. Single analytes results

VEGF (electrochemiluminescence multiplex; angiogenesis panel)

Note: This assay was also tested as part of the cytokine panel (p. 51) were it was classified as not passed
Evaluation: Borderline

Samples not detectable but might be drawn in detection range by spike-in of protein

- Spike-In-Curve has positive difference to standard curve
- Spike in Concentrations are linear in range of standard 4 – 8 at 2.0 pg/ml (2x Dilution)
- Sample concentrations overlap with LLOQ (5.0 pg/ml)
- No significant difference between test samples

A) Spike In Curve

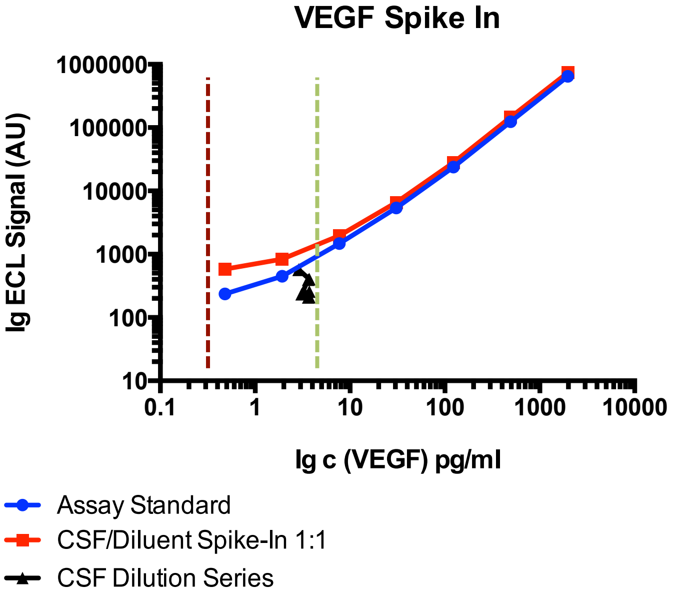

B) Recalculated concentrations curve

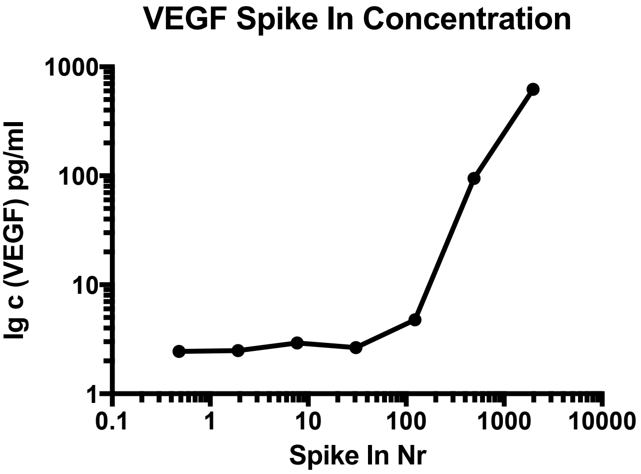

C) CSF dilution series

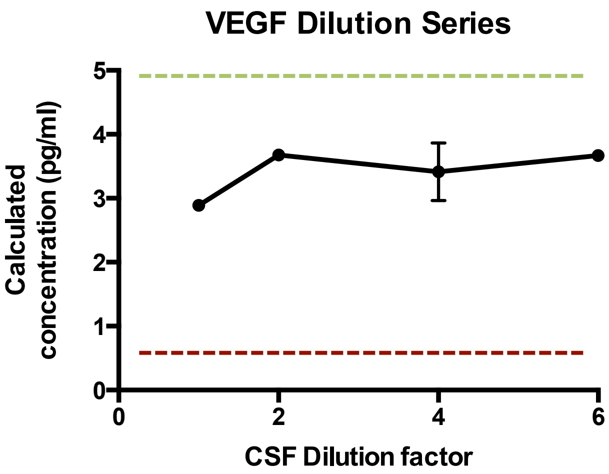

D) Test sample results

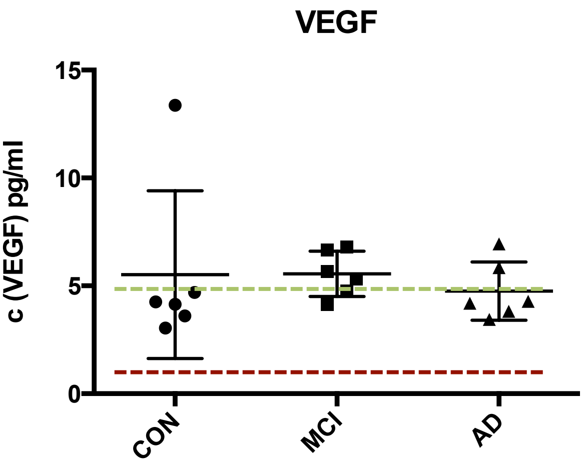

iv. Single analytes results

VEGF-C (electrochemiluminescence multiplex; angiogenesis panel)

Evaluation: Not passed

Samples not detectable

- Spike-In-Curve has positive difference to standard curve
- Spike in concentrations are nonlinear
- Sample concentrations < LLOD (11.1 pg/ml)
- No significant difference between test samples

A) Spike In Curve

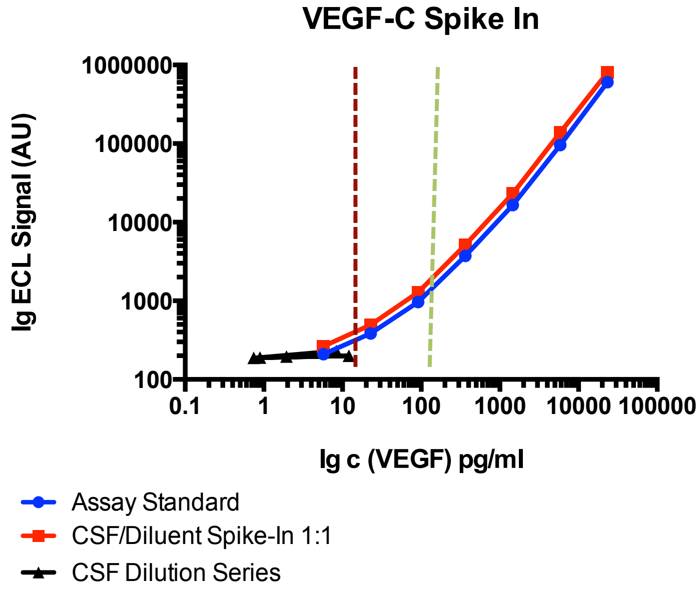

B) Recalculated concentrations curve

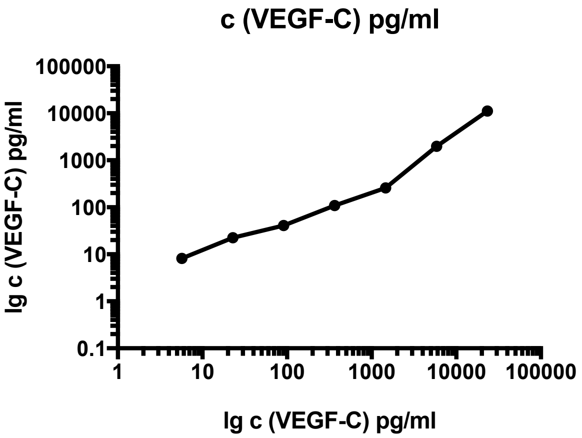

C) CSF dilution series

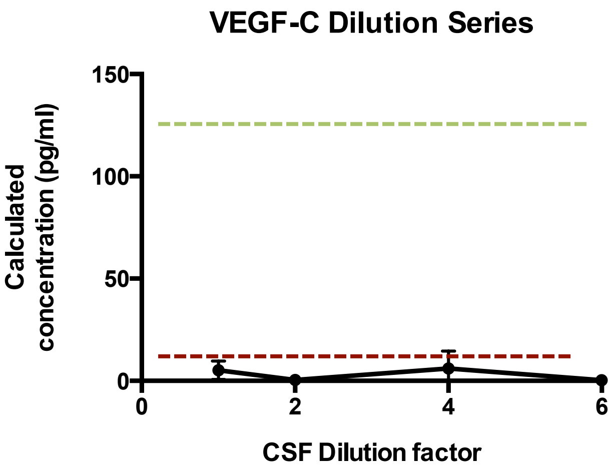

D) Test sample results

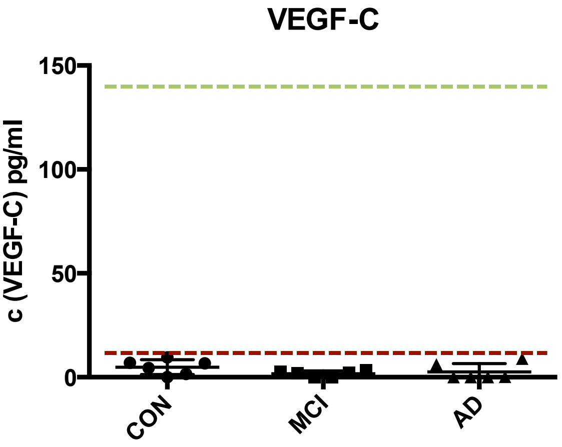

iv. Single analytes results

VEGF-D (electrochemiluminescence multiplex; angiogenesis panel)

Evaluation: Not passed

Samples not detectable

- Spike-In-Curve has positive difference to standard curve
- Spike-In concentrations are nonlinear
- Sample concentrations < LLOQ (67.1 pg/ml)
- No significant difference between test samples

A) Spike In Curve

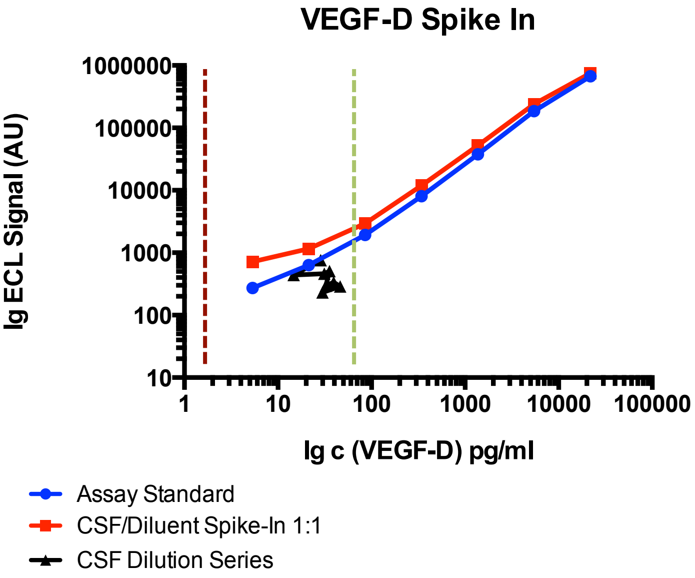

B) Recalculated concentrations curve

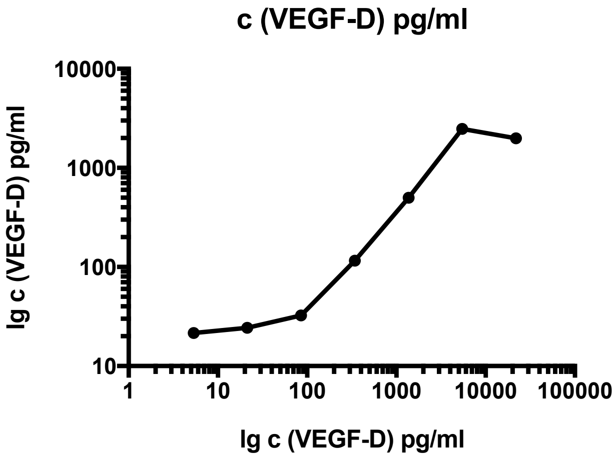

C) CSF dilution series

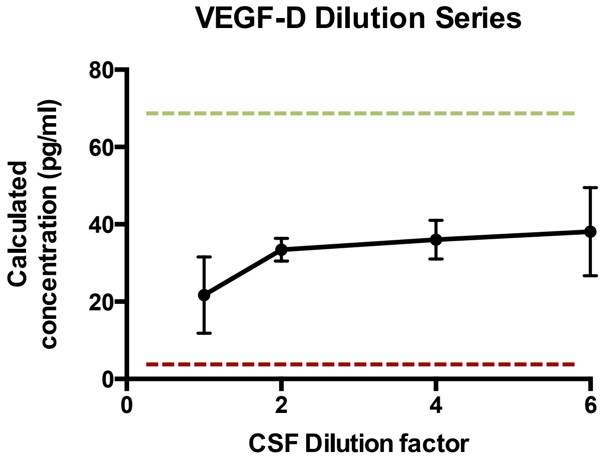

D) Test sample results

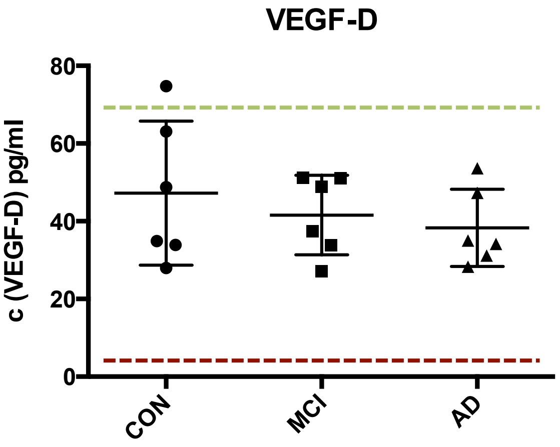

Supplement: Supplementary file 1 — Technical supplement: initial test phase results. (PDF 3888 kb) [file 13195_2018_353_MOESM1_ESM.pdf]
