# Supplementary material for: Characterization and clinical use of inflammatory cerebrospinal fluid protein markers in Alzheimer’s disease
Source: Alzheimers Res Ther. 2018 Feb 26;10:25. doi: 10.1186/s13195-018-0353-3 (PMC5828084; doi:10.1186/s13195-018-0353-3)
Supplement: Supplementary file 2 — Statistical supplement. (PDF 781 kb) [file 13195_2018_353_MOESM2_ESM.pdf]

# Statistical Supplement – Table of contents:

## Tables


| | Tables | Page |
|---|---|---|
| **1** | Data distribution analysis | 2 |
| **2** | Outlier analysis | 3 |
| **3** | Description of main cohort | 4 |
| **4** | Description of supplementary cohort | 6 |
| **5** | Inflammatory protein levels in main cohort | 8 |
| **6** | Inflammatory protein levels in supplementary cohort | 9 |
| **7** | Association of inflammatory proteins with pathology in main cohort | 11 |
| **8** | Influence of ApoE genotype in the main cohort | 13 |
| **9** | Influence of sex on inflammatory protein levels | 14 |
| **10** | Homogeneity of the ND (non-demented) cohort | 15 |
| **11** | Protein ratios | 16 |
| **12** | Confounder adjusted comparisons | 17 |
| **13** | Discriminative power of significant proteins | 18 |
| **14** | Association of inflammatory proteins with neuropsychological performance | 20 |
| **15** | Regression analysis of cognitive correlations | 21 |


## Figures


| | Figures | Page |
|---|---|---|
| **1** | Correlation matrix of MCI and AD cohorts | 22 |

## Table 1: Data distribution analysis

Data distribution was analyzed using D´Agostino-Pearson omnibus test and Quantile-quantile plot analysis (QQ plot analysis) at α = 0.05. None of the tested inflammatory marker data was normal distributed according to both tests. Most of the data followed a distribution slightly skewed towards lower values. SAA and CRP levels followed a lognormal distribution.

| Parameter | D'Agostino-Pearson omnibus test | | QQ plot analysis | |
|---|---|---|---|---|
| | p-value | Data normal distributed? | Fit to normal distribution ($R^2$) | Type of distribution |
| **Age** | 0.0002 | No | 0.95 | normal |
| **Storage Time** | <0.0001 | No | 0.94 | skewed |
| **Aß42** | <0.0001 | No | 0.88 | skewed |
| **Tau** | <0.0001 | No | 0.89 | skewed |
| **p-tau** | <0.0001 | No | 0.91 | skewed |
| **Aß42/40** | 0.0001 | No | 0.95 | normal |
| **Aß42/ptau** | <0.0001 | No | 0.88 | skewed |
| **CSF protein** | <0.0001 | No | 0.85 | skewed |
| **CSF albumin** | <0.0001 | No | 0.86 | skewed |
| **CSF IgG** | <0.0001 | No | 0.71 | skewed |
| **MIF** | <0.0001 | No | 0.70 | skewed |
| **VEGF** | <0.0001 | No | 0.90 | skewed |
| **Flt-1** | <0.0001 | No | 0.96 | normal |
| **Il-8** | <0.0001 | No | 0.88 | skewed |
| **Il-6** | <0.0001 | No | 0.75 | skewed |
| **MCP-1** | <0.0001 | No | 0.81 | inconclusive |
| **IP-10** | <0.0001 | No | 0.74 | skewed |
| **ICAM-1** | <0.0001 | No | 0.92 | skewed |
| **VCAM-1** | <0.0001 | No | 0.84 | skewed |
| **SAA** | <0.0001 | No | 0.16 | lognormal |
| **CRP** | <0.0001 | No | 0.01 | lognormal |
| **C1q** | <0.0001 | No | 0.69 | inconclusive, |
| **C3a** | <0.0001 | No | 0.72 | skewed |
| **Il1RAcP** | <0.0001 | No | 0.83 | skewed |
| **TREM2** | <0.0001 | No | 0.84 | skewed |

## Table 2: Outlier analysis

Samples with high values of at least one inflammatory protein marker were considered as outliers (value sorted by decreasing size; values at least 2-fold higher than next value in list considered outliers). Medical records of respective patients were searched for conditions with potential influence on markers.

| Potential Outlier No. | Suspicious values | Medical Record | Conclusion |
|---|---|---|---|
| **1** | MCP-1 | History of stroke, but no acute comorbidities | Sample not excluded |
| **2** | CRP | No indication of comorbidities | Sample not excluded |
| **3** | High erythrocytes | Blood contamination during LP in first fractions, research sample obtained from “clear” fraction | Sample not excluded as aliquots used for study where free of blood |
| **4** | Il1-RAcP | No indication of comorbidities | Sample not excluded |
| **5** | Il-8 | No indication of comorbidities | Sample not excluded |
| **6** | Il-8 | No indication of comorbidities | Sample not excluded |
| **7** | Il-8 | No indication of comorbidities | Sample not excluded |
| **8** | Il-8 | No indication of comorbidities | Sample not excluded |
| **9** | Il-8, sICAM | No indication of comorbidities | Sample not excluded |

**Table 3: Description of main cohorts**

This table is redundant to table 1 from the main text, but includes the exact p values of cohort comparisons. Overview of the main patient cohorts (ND, MCI and AD cases), potential confounders such as age, sex and biobank storage time, as well as standard AD biomarker and neuropsychology findings. Results are described by median ± standard deviation, range and coverage in the dataset (in percent), and statistical results (p values of Kruskal-Wallis test and significant post-hoc comparisons adjusted by the Bonferroni method). Consistent with the difference in diagnosis, CSF biomarkers and neuropsychological results were highly discriminative. Age, sex and storage time varied between cohorts and were therefore considered as confounders in subsequent covariate testing.

| **Feature** | **Main Clinical Cohorts** | | | **p-value** (Significant cohort-wise comparisons) |
|---|---|---|---|---|
| | **ND** | **MCI** | **AD** | |
| **Number (n)** | 85 | 130 | 116 | - |
| **Age (yrs.)** | 67 ± 11<br>43 - 81; 100% | 71 ± 8<br>48 - 85; 100% | 74 ± 8<br>50 - 88; 100% | **$1x10E^{-10}$**<br>(ND vs. MCI, AD) |
| **Sex (% male)** | 65 | 65 | 45 | **$Chi^2 = 2x10E^{-3}$**<br>(AD Vs. ND, MCI) |
| **Storage Time (mth.)** | 27 ± 21<br>0.3 - 95.0; 100% | 36 ± 23<br>4.0 - 91.0; 100% | 22 ± 19<br>4.0 - 94.0; 100% | **$7x10E^{-5}$**<br>(MCI Vs. ND, AD) |
| **Aβ42 pg/ml** | 552 ± 315<br>138 – 1653; 100% | 432 ± 302<br>87 – 1326; 100% | 319 ± 191<br>91 – 1505; 100% | **$1x10E^{-9}$**<br>(All cohorts) |
| **Aβ42/40** | 0.10 ± 0.03<br>0.02 – 0.25; 100% | 0.07 ± 0.04<br>0.02 – 0.23; 100% | 0.05 ± 0.02<br>0.03 – 0.15; 100% | **$1x10E^{-23}$**<br>(All cohorts) |
| **t-tau pg/ml** | 289 ± 228<br>36 – 1210; 100% | 419 ± 290<br>71 – 1528; 100% | 680 ± 344<br>126 – 2157; 100% | **$3x10E^{-14}$**<br>(All cohorts) |
| **p-tau-181 pg/ml** | 43 ± 25<br>16 – 157; 100% | 59 ± 30<br>18 – 158; 100% | 76 ± 36<br>23 – 248; 100% | **$1x10E^{-12}$**<br>(All cohorts) |
| **Aβ42/p-tau-181** | 17.0 ± 9.2<br>2.0 – 42.8; 100% | 9.7 ± 9.1<br>1.5 – 48.8; 100% | 4.5 ± 3.8<br>1.3 – 25.2; 100% | **$6x10E^{-27}$**<br>(All cohorts) |
| **CSF protein (mg/l)** | 488 ± 167<br>257 – 999; 93% | 458 ± 148<br>248 – 1090; 98% | 453 ± 144<br>245 – 1055; 99% | 0.423 |
| **CSF Albumin (mg/l)** | 268 ± 137<br>103 – 688; 88% | 256 ± 116<br>100 – 724; 98% | 260 ± 116<br>118 – 803; 98% | 0.485 |
| **CSF IgG (mg/l)** | 31 ± 17<br>8 – 94; 88% | 28 ± 13<br>9 – 108; 98% | 28 ± 16<br>11 – 128; 98% | 0.263 |
| **CSF leukocytes (cells/µl)** | 1 ± 2<br>0 – 11; 93% | 1 ± 1<br>0 – 7; 98% | 1 ± 1<br>0 – 7; 99% | 0.942 |
| **MMSE** | 29 ± 1<br>27 – 30; 73% | 26 ± 3<br>18 – 30; 91% | 21 ± 4<br>6 – 29; 96% | **$1x10E^{-48}$**<br>(All cohorts) |
| **Education (yrs.)** | 15 ± 2<br>11 – 18; 18% | 14 ± 4<br>8 – 22; 82% | 13 ± 4<br>8 – 22; 72% | 0.129 |
| **Word list recall** | 20 ± 3<br>16 – 23; 18% | 16 ± 4<br>5 – 27; 81% | 13 ± 4<br>3 – 19; 69% | **$1x10E^{-11}$**<br>(All cohorts) |
| **Word list delayed recall** | 6 ± 1<br>4 – 9; 18% | 4 ± 2<br>0 – 10; 78% | 2 ± 2<br>0 – 7; 60% | **$6x10E^{-16}$**<br>(All cohorts) |
| **Word list discrimination (%)** | 100 ± 3<br>90 – 100; 18% | 95 ± 15<br>0 – 100; 81% | 85 ± 21<br>0 – 100; 69% | **$7x10E^{-6}$**<br>(All cohorts) |
| **Figure recall** | 8 ± 2<br>2 – 11; 16% | 5 ± 3<br>0 – 11; 81% | 3 ± 3<br>0 – 10; 60% | **$4x10E^{-10}$**<br>(All cohorts) |
| **TMT A** | 52 ± 27<br>20 – 119; 16% | 57 ± 33<br>26 – 180; 80% | 84 ± 46<br>29 – 180; 69% | **$4x10E^{-3}$**<br>(AD Vs. ND, MCI) |
| **TMT B** | 115 ± 50<br>53 – 245; 16% | 166 ± 80<br>52 – 300; 75% | 300 ± 71<br>76 – 300; 60% | **$5x10E^{-11}$**<br>(All cohorts) |
| **Semantic Fluency** | 22 ± 7<br>14 – 39; 18% | 16 ± 6<br>5 – 34; 81% | 12 ± 5<br>3 – 26; 72% | **$2x10E^{-10}$**<br>(All cohorts) |
| **Phonetic Fluency** | 12 ± 4<br>7 – 19; 16% | 10 ± 5<br>1 – 26; 79% | 9 ± 4<br>2 – 22; 70% | **$7x10E^{-3}$**<br>(MCI Vs. AD) |
| **FCSRT free** | 32 ± 8<br>14 – 40; 14% | 19 ± 8<br>0 – 40; 63% | 9 ± 8<br>0 – 33; 42% | **$2x10E^{-12}$**<br>(All cohorts) |
| **FCSRT Sum** | 48 ± 3<br>45 – 57; 14% | 47 ± 8<br>0 – 48; 63% | 36 ± 15<br>2 – 48; 42% | **$6x10E^{-10}$**<br>(AD Vs. ND, MCI) |

**Table 4: Description of supplementary cohort**

The table provides an overview of the supplementary patient cohorts (PD, DLB, FTD and ALS cases), potential confounders such as age, sex and biobank storage time, as well as standard AD biomarker and NPT findings. Each feature is described with median value ± standard deviation, Minimum to maximum value and coverage of data within the dataset (in %). N.A.: Data not available for at least one patient. Respective data for the larger main cohort is described in the article.

42

| Feature | PD | DLB | FTD | ALS |
|---|---|---|---|---|
| **Number (n)** | 16 | 11 | 20 | 21 |
| **Age (yrs.)** | 73 ± 7<br>57 - 85; 100% | 71 ± 8<br>56 - 83; 100% | 62 ± 9<br>42 - 77; 100% | 67 ± 10<br>42 - 83; 100% |
| **Gender (% male)** | 75 | 73 | 65 | 57 |
| **Storage Time (mth.)** | 28 ± 22<br>4.0 - 72.0; 100% | 38 ± 22<br>2.0 - 69.0; 100% | 51 ± 22<br>4.0 - 85.0; 100% | 42 ± 22<br>4.0 – 80.0; 100% |
| **Aβ42 pg/ml** | 522 ± 323<br>141 - 1160; 100% | 346 ± 202<br>200 - 869; 100% | 488 ± 198<br>187 - 901; 100% | 583 ± 214<br>247 - 917; 100% |
| **Aβ42/40** | 0.09 ± 0.02<br>0.04 – 0.11; 100% | 0.06 ± 0.02<br>0.03 – 0.11; 100% | 0.09 ± 0.02<br>0.06 – 0.12; 100% | 0.09 ± 0.02<br>0.06 – 0.13; 100% |
| **t-tau pg/ml** | 249 ± 306<br>98 - 1404; 100% | 407 ± 184<br>54 - 666; 100% | 437 ± 296<br>81 - 1039; 100% | 250 ± 151<br>48 - 581; 100% |
| **p-tau-181 pg/ml** | 45 ± 28<br>17 - 137; 100% | 54 ± 22<br>30 - 92; 100% | 40 ± 30<br>20 - 140; 100% | 39 ± 13<br>21 - 83; 100% |
| **Aβ42/p-tau-181** | 11.2 ± 6.1<br>0.1 – 21.9; 100% | 8.7 ± 5.4<br>2.8 – 22.7; 100% | 12.7 ± 6.4<br>3.5 – 24.2; 100% | 13.6 ± 6.5<br>0.1 – 26.3; 100% |
| **CSF protein (mg/l)** | 501 ± 173<br>362 - 968; 94% | 545 ± 188<br>437 - 982; 91% | 464 ± 109<br>269 - 675; 100% | 446 ± 181<br>233 - 1047; 100% |
| **CSF Albumin (mg/l)** | 309 ± 130<br>141 - 608; 88% | 320 ± 153<br>218 - 676; 91% | 266 ± 96<br>133 - 477; 100% | 216 ± 154<br>113 - 726; 90% |
| **CSF IgG (mg/l)** | 32 ± 17<br>14 - 70; 88% | 34 ± 13<br>27 - 65; 91% | 25 ± 10<br>11 - 47; 100% | 27 ± 18<br>14 - 66; 90% |
| **CSF leukocytes (cells/µl)** | 2 ± 2<br>0 – 9; 94% | 2 ± 1<br>1 – 4; 91% | 1 ± 2<br>0 – 10; 100% | 1 ± 2<br>0 – 7; 100% |
| **MMSE** | 25 ± 4<br>16 - 27; 38% | 21 ± 5<br>13 - 27; 82% | 24 ± 4<br>15 - 30; 95% | 30<br>5% (single value) |
| **Education (yrs.)** | 15 ± 1<br>11 – 18; 13% | 13 ± 4<br>8 – 22; 64% | 16 ± 4<br>8 – 22; 80% | N.A. |
| **Word list recall** | 13 ± 8<br>7 – 18; 13% | 11 ± 4<br>4 - 15; 64% | 19 ± 7<br>3 - 23; 70% | N.A. |
| **Word list delayed recall** | 4<br>6% (single value) | 4 ± 3<br>1 - 7; 45% | 6 ± 2<br>2 - 9; 60% | N.A. |
| **Word list discrimination (%)** | 83 ± 25<br>65 - 100; 13% | 95 ± 10<br>75 - 100; 55% | 93 ± 17<br>50 - 100; 70% | N.A. |
| **Figure recall** | 8<br>6% (single value) | 7 ± 1<br>6 - 8; 36% | 7 ± 3<br>2 - 11; 55% | N.A. |
| **TMT A** | 113 ± 95<br>46 - 180; 13% | 127 ± 51<br>57 - 180; 64% | 58 ± 36<br>30 - 142; 65% | N.A. |
| **TMT B** | 213 ± 123<br>126 - 300; 13% | 300 ± 44<br>201 - 300; 45% | 137 ± 85<br>62 - 300; 60% | N.A. |
| **Semantic Fluency** | 14 ± 7<br>9 - 19; 13% | 11 ± 4<br>2 - 14; 64% | 10 ± 5<br>2 - 22; 75% | N.A. |
| **Phonetic Fluency** | 9<br>6% (single value) | 6 ± 3<br>1 - 10; 55% | 7 ± 5<br>1 - 18; 65% | N.A. |
| **FCSRT free** | 16<br>6% (single value) | 15 ± 13<br>5 - 24; 18% | 18 ± 7<br>12 - 32; 45% | N.A. |
| **FCSRT Sum** | 48<br>6% (single value) | 39 ± 13<br>29 - 48; 18% | 48 ± 5<br>32 - 48; 45% | N.A. |

## Table 5: Inflammatory protein levels in main cohort

The table shows the results of cohort-wise comparisons for the inflammatory protein panel in the main cohort of ND, MCI and AD patients. For each protein, median, standard deviation, minimum and maximum values are provided. Significant results are highlighted bold. For pairwise inter-cohort comparisons, Bonferroni adjusted p values are reported and significant at α = 0.05. In this setting, CRP and sTREM2 showed significant differences between the ND cohort on the one hand and MCI as well as AD cohorts on the other. A comparison including the supplementary cohort is included in the article.

| Protein | ND | MCI | AD | Kruskal-Wallis p | Pairwise adjusted p |
|---|---|---|---|---|---|
| **VEGF (pg/ml)** | 6.0 ± 3.1<br>0.0 - 18.6 | 6.0 ± 2.3<br>2.0 - 19.8 | 5.6 ± 3.3<br>0.0 - 25.3 | 0.168 | - |
| **sVEGFR-1 (pg/ml)** | 47.3 ± 23.7<br>18.2 - 139.4 | 49.3 ± 16.7<br>21.8 - 102.5 | 51.9 ± 15.9<br>21.0 - 102.6 | 0.276 | - |
| **MCP-1 (pg/ml)** | 323 ± 112<br>169 - 753 | 342 ± 102<br>204 - 948 | 342 ± 96<br>183 - 644 | 0.541 | - |
| **IP-10 (pg/ml)** | 283 ± 173<br>76 - 901 | 290 ± 172<br>66 - 953 | 306 ± 295<br>69 - 1662 | 0.549 | - |
| **Il-6 (pg/ml)** | 0.9 ± 0.8<br>0.0 - 6.0 | 1.1 ± 1.0<br>0.0 - 6.6 | 1.0 ± 0.8<br>0.0 - 5.3 | 0.127 | - |
| **Il-8 (pg/ml)** | 42.9 ± 15.8<br>18.2 - 147.9 | 45.1 ± 32.8<br>26.1 - 346.5 | 44.7 ± 13.9<br>24.9 - 102.7 | 0.354 | - |
| **SAA (pg/ml)** | 1377 ± 9100<br>383 - 64597 | 1264 ± 2405<br>148 - 22212 | 1342 ± 3994<br>205 - 37163 | 0.790 | - |
| **CRP (pg/ml)** | 5410 ± 12840<br>262 - 67633 | 3111 ± 9868<br>135 - 59843 | 2750 ± 10767<br>372 - 64675 | **$4x10E^{-4}$**** | ND-MCI: 0.003<br>ND-AD: $5x10E^{-4}$ |
| **sICAM-1 (pg/ml)** | 2730 ± 1105<br>1138 - 6684 | 3061 ± 1272<br>1287 - 10583 | 3005 ± 1024<br>1377 - 6107 | 0.321 | - |
| **sVCAM-1 (pg/ml)** | 8188 ± 3108<br>4116 - 23498 | 8946 ± 3069<br>3631 - 21950 | 8753 ± 2473<br>4700 - 15076 | 0.321 | - |
| **MIF (pg/ml)** | 1932 ± 1227<br>745 - 8530 | 2030 ± 1432<br>970 - 12779 | 2021 ± 846<br>856 - 6681 | 0.421 | - |
| **C1q (ng/ml)** | 197 ± 80<br>82 - 383 | 209 ± 91<br>73 - 642 | 215 ± 84<br>62 - 567 | 0.463 | - |
| **C3aDesArg (ng/ml)** | 26.9 ± 38.4<br>0 - 197.6 | 29.9 ± 28.9<br>6.1 - 185.6 | 28.3 ± 21.4<br>4.6 - 151.5 | 0.295 | - |
| **sIl-1RAcP (pg/ml)** | 2982 ± 2035<br>1228 - 10469 | 3503 ± 2604<br>519 - 15714 | 3589 ± 3039<br>1363 - 26852 | 0.351 | - |
| **sTREM2 (pg/ml)** | 2994 ± 2269<br>690 - 13746 | 4071 ± 2544<br>795 - 12950 | 4315 ± 2235<br>756 - 11965 | **0.001**** | ND-MCI: 0.004<br>ND-AD: 0.002 |

**Table 6: Inflammatory protein levels in supplementary cohort**

The table shows the distribution of inflammatory marker levels in the cohorts of PD; DLB, FTD and ALS patients. Significant results in Kruskal-Wallis tests are highlighted bold. For pairwise comparisons, Bonferroni adjusted p values are reported and significant at $\alpha = 0.05$ (adjusted for pairwise comparisons of each cohort against each other). In this setting, CRP and sTREM2 and their ratio still differentiated between the main cohorts. Several other markers were significant in comparisons based on the supplemental cohorts with different strength of the respective adjusted pairwise comparisons. “None significant” indicates that pairwise comparisons did not sustain Bonferroni adjustment.

| Protein | PD | DLB | FTD | ALS | Kruskal-Wallis p | Pairwise adjusted p |
|---|---|---|---|---|---|---|
| **Number (n)** | 16 | 11 | 20 | 21 | | |
| **VEGF (pg/ml)** | 6.1 ± 2.1<br>3.5 – 11.0 | 5.9 ± 1.8<br>3.4 – 8.4 | 5.0 ± 1.2<br>2.9 – 7.2 | 4.6 ± 3.9<br>3.1 – 17.9 | **0.038** | None significant |
| **sVEGFR-1 (pg/ml)** | 41.1 ± 13.2<br>21.4 – 70.6 | 40.4 ± 9.7<br>30.0 – 60.1 | 36.4 ± 11.1<br>25.2 – 76.6 | 36.0 ± 10.4<br>28.8 – 69.0 | **1x10E$^{-6}$** | FTD-ND: 0.003<br>FTD-MCI: 0.003<br>FTD-AD: 7x10E$^{-6}$<br>ALS-ND: 0.046<br>ALS-MCI: 0.047<br>ALS-AD: 0.002 |
| **MCP-1 (pg/ml)** | 335 ± 73<br>217 - 473 | 309 ± 110<br>194 - 565 | 336 ± 112<br>202 - 722 | 335 ± 112<br>194 - 637 | 0.862 | - |
| **IP-10 (pg/ml)** | 217 ± 79<br>81 - 363 | 285 ± 185<br>84 - 728 | 198 ± 126<br>48 - 638 | 198 ± 108<br>44 - 528 | **5x10E$^{-4}$** | FTD-ND: 0.037<br>FTD-MCI: 0.038<br>FTD-AD: 0.006<br>ALS-AD: 0.033 |
| **Il-6 (pg/ml)** | 1.3 ± 0.3<br>1-0 – 2.3 | 1.2 ± 1.1<br>0.9 – 4.7 | 1.1 ± 0.4<br>0.6 – 1.8 | 1.3 ± 0.4<br>0.8 – 2.5 | **4x10E$^{-5}$** | ALS-ND: 0.006<br>PD-ND: 0.005<br>PD-AD: 0.036 |
| **Il-8 (pg/ml)** | 49.1 ± 9.1<br>37.9 – 67.1 | 51.4 ± 8.7<br>37.6 – 64.7 | 41.4 ± 7.8<br>26.9 – 55.3 | 48.7 ± 11.9<br>33.3 – 80.8 | **0.038** | None significant |
| **SAA (pg/ml)** | 1415 ± 1707<br>478 - 7173 | 1664 ± 13463<br>533 - 43319 | 851 ± 1097<br>244 - 5009 | 1071 ± 758<br>524 - 3538 | 0.087 | - |
| **CRP (pg/ml)** | 3906 ± 6556<br>984 - 20099 | 4735 ± 11074<br>521 - 28337 | 2910 ± 6794<br>370 - 26426 | 5792 ± 6567<br>458 - 29691 | **0.001** | ND-MCI: 0.022<br>ND-AD: 0.003 |
| **sICAM-1 (pg/ml)** | 3465 ± 858<br>2457 - 5704 | 4129 ± 748<br>3053 - 5849 | 2909 ± 1098<br>1599 - 6156 | 3427 ± 835<br>1988 - 5442 | **0.005** | DLB-ND: 0.006<br>DLB-MCI: 0.040 |
| **sVCAM-1 (pg/ml)** | 9055 ± 3188<br>5228 - 15110 | 9479 ± 2111<br>6535 - 13866 | 7818 ± 2214<br>4008 - 13119 | 8619 ± 1831<br>5163 - 11832 | 0.481 | - |
| **MIF (pg/ml)** | 1966 ± 961<br>827 - 4586 | 1837 ± 515<br>1279 - 2708 | 1611 ± 1362<br>935 - 7176 | 1432 ± 368<br>958 - 2683 | **5x10E$^{-4}$** | ALS-ND: 0.005<br>ALS-MCI: 2x10E$^{-4}$<br>ALS-AD: 0.002 |
| **C1q (ng/ml)** | 233 ± 73<br>101 - 386 | 212 ± 57<br>127 - 330 | 189 ± 48<br>92 - 280 | 192 ± 70<br>120 - 360 | 0.555 | - |
| **C3aDesArg (ng/ml)** | 33.2 ± 23.5<br>17.0 – 101.9 | 37.2 ± 38.3<br>16.4 – 141.5 | 30.8 ± 30.3<br>11.3 – 141.2 | 23.7 ± 24.7<br>12.4 – 83.9 | 0.272 | - |
| **sIl-1RAcP (pg/ml)** | 5925 ± 3424<br>3053 - 15887 | 6705 ± 3152<br>3839 - 13051 | 4310 ± 2422<br>2213 - 11079 | 4382 ± 2341<br>1587 - 9897 | **2x10E$^{-7}$** | PD-ND: 2x10E$^{-4}$<br>PD-MCI: 0.001<br>PD-AD: 0.003<br>DLB-ND: 5x10E$^{-4}$<br>DLB-MCI: 0.002<br>DLB-AD: 0.005 |
| **sTREM2 (pg/ml)** | 4186 ± 2243<br>1796 - 8978 | 4222 ± 1675<br>2853 - 8785 | 3343 ± 2031<br>525 - 8826 | 3623 ± 2456<br>1245 - 9680 | **0.017** | ND-MCI: 0.025<br>ND-AD: 0.016 |

**Table 7: Association between inflammatory and AD biomarkers in main cohort**

The table shows the results of cohort-wise comparisons for the inflammatory protein panel if samples of the main cohort are divided by pathological vs. non-pathological levels of amyloid or tau biomarkers. Discrimination was based on the following cutoff values: Aβ42 350 pg/ml, Aβ42/40 0.07, t-tau 470 pg/ml, p-tau-181 56 pg/ml. Significant results are highlighted bold. Values are provided with Mann-Whitney U test p values and, for significant findings, Spearman correlation p – and r values derived from the correlation matrix. In this setting, there were a large number of markers consistently associated with tau pathology. Some of these were likewise associated with pathological amyloid values.

| Protein | By Aβ42 | By Aβ42/40 | By t-tau | By p-tau-181 |
|---|---|---|---|---|
| **VEGF** | 0.419 | **0.044**<br>p = 0.007<br>r = 0.148 | **0.027**<br>p = 0.005<br>r = -0.156 | **0.008**<br>p = 0.004<br>r = -0.160 |
| **sVEGFR-1** | **0.003**<br>p = 1x10E$^{-4}$<br>r = 0.208 | **0.033**<br>p = 0.019<br>r = -0.129 | **2x10E$^{-12}$**<br>p = 6x10E$^{-21}$<br>r = 0.485 | **1x10E$^{-15}$**<br>p = 3x10E$^{-28}$<br>r = 0.556 |
| **MCP-1** | 0.963 | 0.190 | **0.006**<br>p = 0.001<br>r = 0.185 | 0.101 |
| **IP-10** | 0.951 | 0.773 | **0.024**<br>p = 0.001<br>r = 0.185 | **0.034**<br>p = 0.004<br>r = 0.157 |
| **Il-6** | 0.832 | 0.876 | 0.513 | 0.644 |
| **Il-8** | 0.290 | 0.336 | 0.053 | 0.232 |
| **SAA** | 0.079 | 0.104 | 0.443 | 0.102 |
| **CRP** | **0.005**<br>p = 0.013<br>r = 0.136 | **3x10E$^{-5}$**<br>p = 9x10E$^{-5}$<br>r = 0.214 | **0.005**<br>p = 0.001<br>r = -0.175 | **0.002**<br>p = 0.003<br>r = -0.162 |
| **sICAM-1** | 0.118 | **0.033**<br>p = 0.075<br>r = -0.098 | **3x10E$^{-4}$**<br>p = 8x10E$^{-5}$<br>r = 0.215 | **2x10E$^{-4}$**<br>p = 8x10E$^{-7}$<br>r = 0.267 |
| **sVCAM-1** | **0.015**<br>p = 0.095<br>r = 0.086 | **0.042**<br>p = 0.131<br>r = -0.083 | **2x10E$^{-6}$**<br>p = 3x10E$^{-8}$<br>r = 0.300 | **1x10E$^{-8}$**<br>p = 5x10E$^{-15}$<br>r = 0.413 |
| **MIF** | 0.699 | 0.067 | **2x10E$^{-11}$**<br>p = 2x10E$^{-15}$<br>r = 0.419 | **3x10E$^{-11}$**<br>p = 1x10E$^{-20}$<br>r = 0.481 |
| **C1q** | **0.017**<br>p = 0.063<br>r = 0.102 | 0.500 | **3x10E$^{-8}$**<br>p = 3x10E$^{-11}$<br>r = 0.354 | **2x10E$^{-10}$**<br>p = 2x10E$^{-14}$<br>r = 0.403 |
| **C3aDesArg** | 0.422 | 0.606 | 0.888 | 0.611 |
| **sIl-1RAcP** | 0.810 | **0.036**<br>p = 0.117<br>r = -0.086 | **0.046**<br>p = 0.019<br>r = 0.128 | 0.109 |
| **sTREM2** | 0.070 | **0.002**<br>p = 0.037<br>r = -0.115 | **2x10E$^{-12}$**<br>p = 4x10E$^{-15}$<br>r = 0.414 | **9x10E$^{-11}$**<br>p = 2x10E$^{-17}$<br>r = 0.444 |

## Table 8: Influence of ApoE genotype in the main cohort

ApoE genotype data was available for 213 samples of the main cohort (18 ND, 102 MCI and 93 AD cases). Comparisons were based either on the exact genotype (2/2, 2/3, 2/4, 3/3, 3/4, 4/4) or the number of ApoE4 alleles (0, 1, or 2 alleles). Significant results from Kruskal-Walli-tests are highlighted bold. For pairwise comparisons, Bonferroni-adjusted p values are reported and significant at α = 0.05. CRP was the only inflammatory marker significantly associated with ApoE genotype with a tendency of lower values associated with increasing number of ApoE4 alleles. Homozygous 4/4 carriers compared to non-carriers drove this effect.

| Protein/Marker | By exact ApoE genotype | | By number of ApoE4 alleles | |
|---|---|---|---|---|
| | Kruskal-Wallis p | Pairwise adjusted p | Kruskal-Wallis p | Pairwise adjusted p |
| **Aβ42** | **0.040** | 4/4 – 3/3: 0.023 | **0.004** | 2-1: 0.022<br>2-0: 0.003 |
| **Aβ42/40** | **9x10E$^{-5}$** | 4/4 – 3/3: 1x10E$^{-4}$<br>4/4 – 2/3: 0.042 | **1x10E$^{-3}$** | 2-0: 5x10E$^{-3}$<br>1-0: 0.008 |
| **t-tau** | 0.053 | - | **0.019** | 2-0: 0.022 |
| **p-tau-181** | **0.008** | 4/4 – 2/3: 0.007 | **0.004** | 2-0: 0.005 |
| **Aβ42/p-tau-181** | **2x10E$^{-4}$** | 4/4 – 3/3: 0.001<br>4/4 – 2/3: 0.034 | **4x10E$^{-5}$** | 2-0: 6x10E$^{-5}$<br>1-0: 0.012 |
| **VEGF** | 0.516 | - | 0.403 | - |
| **sVEGFR-1** | 0.194 | - | 0.505 | - |
| **MCP-1** | 0.571 | - | 0.146 | - |
| **IP-10** | 0.435 | - | 0.737 | - |
| **Il-6** | 0.571 | - | 0.524 | - |
| **Il-8** | 0.416 | - | 0.267 | - |
| **SAA** | 0.108 | - | 0.104 | - |
| **CRP** | **0.007** | 4/4 – 3/3: 0.019 | **0.009** | 2-0: 0.014 |
| **sICAM-1** | 0.061 | - | 0.294 | - |
| **sVCAM-1** | 0.316 | - | 0.404 | - |
| **MIF** | 0.231 | - | 0.991 | - |
| **C1q** | 0.216 | - | 0.602 | - |
| **C3aDesArg** | 0.065 | - | 0.150 | - |
| **sIl-1RAcP** | 0.330 | - | 0.157 | - |
| **sTREM2** | 0.119 | - | 0.071 | - |

## Table 9: Influence of sex on inflammatory protein levels

Levels of several inflammatory proteins varied significantly between male and female subjects. Significant results from Mann-Whitney-U tests are highlighted bold. For those markers that showed a significant test result, the tendency was always that levels were higher in males compared to females.

| Protein | Mann-Whitney-U p | Median (male) pg/ml | Median (female) pg/ml |
|---|---|---|---|
| **VEGF** | **0.004*** | 6.1 | 5.5 |
| **sVEGFR-1** | 0.226 | 48.5 | 51.6 |
| **MCP-1** | **0.001**** | 361 | 319 |
| **IP-10** | 0.327 | 299 | 277 |
| **Il-6** | **0.005*** | 1.1 | 0.9 |
| **Il-8** | **0.002*** | 46.1 | 42.8 |
| **SAA** | 0.983 | 1341 | 1348 |
| **CRP** | **0.036*** | 3724 | 2775 |
| **sICAM-1** | **$5x10E^{-7}$**** | 3216 | 2583 |
| **sVCAM-1** | **$1x10E^{-8}$**** | 9607 | 7642 |
| **MIF** | 0.177 | 2045 | 1953 |
| **C1q** | **$1x10E^{-7}$**** | $233x10E^{3}$ | $189 x10E^{3}$ |
| **C3aDesArg** | **$9x10E^{-5}$**** | $31.9x10E^{3}$ | $24.4 x10E^{3}$ |
| **sIl-1RAcP** | **0.003**** | 3612 | 2969 |
| **sTREM2** | **0.004*** | 2650 | 2373 |

## Table 10: Homogeneity of the ND (non-demented) cohort

The ND cohort consisted of patients classified by medical diagnosis as follows: Depression (DEP, n = 6), excluded neuroborreliosis (EN, n = 5), polyneuropathy (PNP, n = 28), other movement disorders (MD, n = 2), subjective cognitive impairment without pathological neuropsychological results (SCI, n = 16), normal pressure hydrocephalus without dementia (NPH, n = 8), idiopathic intracranial hypertension (IIH, n = 3), others (O, n = 17). Classes were based on existence of at least 2 identical clinical diagnoses within the total of ND. Significant results from Kruskal-Wallis tests are highlighted bold. For pairwise comparisons, Bonferroni-adjusted p values are reported and significant at α = 0.05 (adjusted for pairwise comparisons of each cohort against each other). For several markers, there was a tendency of subcohort variances that was not strong enough to be traceable in pairwise comparisons. For 4 markers (VEGF, VEGFR, Il-6 and sIl1-RAcP) the NPH subcohort showed significant differences to at least one other subcohort. Among these, the most robust (in terms of cohort sizes) was the difference of VEGFR levels between NPH and PNP.

| Protein | Kruskal-Wallis p | Pairwise adjusted p |
|---|---|---|
| **VEGF** | **0.047** | NPH-EN; p = 0.042 |
| **sVEGFR-1** | **0.001** | NPH-EN; p = 0.006<br>NPH-PNP; p = 0.008<br>NPH-MD; p = 0.043 |
| **MCP-1** | 0.090 | - |
| **IP-10** | **0.023** | None after adjustment |
| **Il-6** | **0.012** | NPH-Depression; p = 0.028 |
| **Il-8** | **0.024** | None after adjustment |
| **SAA** | 0.182 | - |
| **CRP** | 0.260 | - |
| **sICAM-1** | **0.041** | None after adjustment |
| **sVCAM-1** | 0.095 | - |
| **MIF** | 0.325 | - |
| **C1q** | 0.180 | - |
| **C3aDesArg** | **0.019** | None after adjustment |
| **sIl-1RAcP** | **0.003** | NPH-IIH; p = 0.007<br>IIH-O; p = 0.040 |
| **sTREM2** | **0.046** | None after adjustment |

## Table 11: Protein ratios

Based on effects observed in the cohort comparison data, we tested how ratios of proteins with inverse effects or tendencies (VEGF and its soluble receptor sVEGFR; CRP and sTREM2) perform in cohort-wise comparisons by Kruskal-Wallis test. The ratio VEGF/sVEGFR did not improve discriminative testing in comparison to sVEGFR alone. The ratio CRP/sTREM2 was a slightly better discriminator than its single components in comparisons based on clinical diagnosis and amyloid. For tau, the ratio was stronger than CRP alone but weaker than sTREM2. Noteworthy, other variants of this ratio (e.g., CRP/sTREM2 or lg CRP/sTREM2) did not result in significant test results.

| Ratio | Cohorts | p value |
|---|---|---|
| **VEGF/sVEGFR** | Main Cohorts | 0.297 |
| | Amyloid | 0.076 |
| | Tau | **$3x10E^{-7}$** |
| **CRP/TREM2** | Main Cohorts | **$1x10E^{-6}$**<br>ND - MCI: $3x10E^{-5}$<br>ND – AD: $4x10E^{-6}$ |
| | Amyloid | **$2x10E^{-7}$** |
| | Tau | **$2x10E^{-8}$** |

**Table 12: Confounder-adjusted comparisons**

Based on correlation and cohort comparison analyses, we considered patient age, sex and biobank storage time as the statistically most influential covariates. These variables differed significantly between clinical cohorts while being in many cases correlated to inflammatory protein levels. For CRP and sTREM2, we also included ApoE genotype. Analysis was restricted to those inflammatory proteins with the most significant findings in previous cohort comparisons. To test adjustments for pathological cohorts, we focused on the highly discriminative ratio Aβ42/40 for amyloid and on t-tau as both tau isoforms were highly correlated. Log-transformed protein concentrations were used to ensure compatibility with parametric ANCOVA testing. Cohorting refers to the comparison under investigation. For each comparison, the covariate p value indicates if the covariate is of significant influence. The adjusted p value is the result of the cohort comparison adjusted for the influence of the covariate and is only reported when covariates had significant influence. Significant findings are highlighted bold. When of significant influence, adjustment for age and, in most cases, biobank storage time lead to less significant test results. In contrast, adjusting for sex frequently improved p values of cohort comparisons. Of the tested proteins, CRP was less strongly affected by age, sex or storage time, but strongly by ApoE genotype, leading to less significant findings after adjustment.

| Protein | Cohort | Cohort p value | Covariance feature | Covariates | | | |
|---|---|---|---|---|---|---|---|
| | | | | Age | Gender | Storage Time | ApoE Alleles |
| **sTREM2** | **Clinical** | 0.001 | **Covariate p** | **$1x10E^{-11}$** | **0.003** | 0.328 | 0.098 |
| | | | *Adjusted p* | **0.270** | **$4x10E^{-4}$** | - | - |
| | **Amyloid** | 0.004 | **Covariate p** | **$3x10E^{-12}$** | **0.003** | 0.226 | 0.059 |
| | | | *Adjusted p* | **0.730** | **0.001** | - | - |
| | **Tau** | $2x10E^{-12}$ | **Covariate p** | **$4x10E^{-9}$** | **0.002** | 0.133 | **0.032** |
| | | | *Adjusted p* | **$3x10E^{-7}$** | **$5x10E^{-13}$** | - | **$9x10E^{-8}$** |
| **CRP** | **Clinical** | $5x10E^{-4}$ | **Covariate p** | 0.095 | 0.072 | 0.753 | **0.017** |
| | | | *Adjusted p* | - | - | - | **0.581** |
| | **Amyloid** | $4x10E^{-5}$ | **Covariate p** | 0.083 | 0.084 | 0.906 | **0.030** |
| | | | *Adjusted p* | - | - | - | **0.048** |
| | **Tau** | 0.019 | **Covariate p** | 0.333 | **0.038** | 0.755 | **0.010** |
| | | | *Adjusted p* | - | **0.023** | - | **0.546** |
| **VEGF** | **Amyloid** | 0.078 | **Covariate p** | 0.121 | **0.004** | **$3x10E^{-6}$** | - |
| | | | *Adjusted p* | - | **0.158** | **0.164** | - |
| | **Tau** | 0.006 | **Covariate p** | 0.066 | **0.003** | **$3x10E^{-6}$** | - |
| | | | *Adjusted p* | - | **0.008** | **0.013** | - |
| **sVEGFR** | **Amyloid** | 0.032 | **Covariate p** | **0.016** | 0.407 | **$3x10E^{-4}$** | - |
| | | | *Adjusted p* | **0.243** | - | **0.062** | - |
| | **Tau** | $8x10E^{-11}$ | **Covariate p** | 0.308 | 0.382 | **$4x10E^{-4}$** | - |
| | | | *Adjusted p* | - | - | **$2x10E^{-10}$** | - |
| **sICAM-1** | **Amyloid** | 0.064 | **Covariate p** | **$8x10E^{-11}$** | **$5x10E^{-7}$** | 0.361 | - |
| | | | *Adjusted p* | **0.585** | **0.012** | - | - |
| | **Tau** | 0.001 | **Covariate p** | **$2x10E^{-9}$** | **$7x10E^{-7}$** | 0.322 | - |
| | | | *Adjusted p* | **0.157** | **$2x10E^{-4}$** | - | - |
| **sVCAM-1** | **Aymloid** | 0.044 | **Covariate p** | **$5x10E^{-9}$** | **$7x10E^{-10}$** | 0.199 | - |
| | | | *Adjusted p* | **0.879** | **0.004** | - | - |
| | **Tau** | $5x10^{-6}$ | **Covariate p** | **$4x10E^{-7}$** | **$5x10E^{-10}$** | 0.149 | - |
| | | | *Adjusted p* | **0.005** | **$4x10E^{-7}$** | - | - |
| **sIl-1RAcP** | **Amyloid** | 0.010 | **Covariate p** | **0.003** | **0.003** | 0.613 | - |
| | | | *Adjusted p* | **0.165** | **0.003** | - | - |
| | **Tau** | 0.025 | **Covariate p** | **0.002** | **0.006** | 0.668 | - |
| | | | *Adjusted p* | **0.267** | **0.017** | - | - |
| **C1q** | **Tau** | $4x10^{-8}$ | **Covariate p** | **$4x10^{-10}$** | **$2x10^{-9}$** | 0.566 | - |
| | | | *Adjusted p* | **0.001** | **$2x10^{-9}$** | - | - |
| **MIF** | **Tau** | 0.028 | **Covariate p** | 0.425 | 0.608 | **$2x10^{-6}$** | - |
| | | | *Adjusted p* | - | - | **0.010** | - |
| **MCP-1** | **Tau** | 0.008 | **Covariate p** | **$2x10^{-7}$** | **$4x10^{-4}$** | 0.389 | - |
| | | | *Adjusted p* | **0.375** | **0.004** | - | - |
| **IP-10** | **Tau** | 0.041 | **Covariate p** | 0.439 | 0.289 | 0.262 | - |
| | | | *Adjusted p* | - | - | - | - |

## Table 13: Discriminative power of significant proteins

The table summarizes the results of discriminative power analysis for proteins with significant results in inter-cohort comparisons. Sensitivity and specificity were equally weighted to determine cutoff values. In average, the sensitivity / specificity reached was around 60%.

| Protein | Cohorts to Discriminate | Cutoff Value | Sensitivity / Specificity (%) |
|---|---|---|---|
| **sTREM2** | ND Vs. MCI | 3542 pg/ml | 58.5 |
| | ND Vs. AD | 3531 pg/ml | 58.6 |
| | Amyloid | 3664 pg/ml | 58.3 |
| | Tau | 3852 pg/ml | 63.5 |
| **CRP** | ND Vs. MCI | 3975 pg/ml | 59.5 |
| | ND Vs. AD | 3858 pg/ml | 58.6 |
| | Amyloid | 3524 pg/ml | 60.4 |
| | Tau | 3197 pg/ml | 57.2 |
| **CRP/sTREM2** | ND Vs. MCI | 1.15 | 60.0 |
| | ND Vs. AD | 1.09 | 60.3 |
| | Amyloid | 1.01 | 62.0 |
| | Tau | 0.90 | 64.2 |
| **VEGF** | Amyloid | 5.9 pg/ml | 53.7 |
| | Tau | 5.9 pg/ml | 54.7 |
| **sVEGFR** | Amyloid | 49.9 pg/ml | 55.1 |
| | Tau | 50.9 pg/ml | 69.8 |
| **sICAM-1** | Amyloid | 2985 pg/ml | 53.5 |
| | Tau | 2965 pg/ml | 57.2 |
| **sVCAM-1** | Aymloid | 8650 pg/ml | 55.1 |
| | Tau | 8691 pg/ml | 61.0 |
| **sIl-1RAcP** | Amyloid | 3472 pg/ml | 52.9 |
| | Tau | 3472 pg/ml | 52.2 |
| **C1q** | Tau | 206 ng/ml | 62.3 |
| **MIF** | Tau | 2026 pg/ml | 65.4 |
| **MCP-1** | Tau | 341 pg/ml | 55.3 |
| **IP-10** | Tau | 297 pg/ml | 56.6 |

**Table 14: Association of inflammatory proteins with neuropsychological performance**

The table lists significant findings obtained by Spearman correlation analysis of inflammatory proteins that showed at least one significant association with NPT results. Significant correlations are highlighted bold. Across the main cohorts (ND, MCI and AD patients), there were 16 significant correlations with low strength ($r < 0.3$). In the MCI cohort alone, there were 7 significant correlations of similar strength, of which 3 where also found in the main cohort. The strongest correlations were found in the AD cohort, which showed a total of 10 significant correlations of which 6 matched the main cohort.

| Protein | Correlate | Main Cohort | | MCI cohort | | AD cohort | |
|---|---|---|---|---|---|---|---|
| | | p | r | p | r | p | r |
| **MIF** | FCSRT free | 0.249 | -0.097 | 0.487 | -0.078 | **0.001** | **-0.458** |
| | FCSRT sum | 0.537 | -0.052 | 0.963 | 0.005 | **3x10E$^{-4}$** | **-0.498** |
| **VEGF** | Wordlist delayed recall | **0.007** | **0.197** | 0.133 | 0.151 | 0.496 | 0.083 |
| | Semantic Fluency | 0.275 | -0.077 | 0.237 | -0.116 | **0.015** | **-0.265** |
| | Phonetic Fluency | **0.035** | **-0.150** | 0.414 | -0.081 | **0.003** | **-0.331** |
| **Il-6** | Figure Recall | 0.111 | 0.116 | **0.048** | **0.193** | 0.389 | 0.105 |
| **Il-8** | TMTB | 0.254 | 0.085 | **0.038** | **-0.209** | 0.490 | -0.084 |
| | FCSRT free | **0.020** | **-0.194** | 0.323 | -0.110 | **0.041** | **-0.293** |
| **SAA** | TMTA | **0.030** | **0.154** | **0.025** | **0.220** | 0.374 | 0.101 |
| | Phonetic Fluency | **0.010** | **-0.182** | 0.146 | -0.144 | **0.001** | **-0.348** |
| **CRP** | Semantic Fluency | 0.227 | -0.085 | 0.376 | -0.087 | **0.036** | **-0.230** |
| | Phonetic Fluency | **0.005** | **-0.197** | 0.120 | -0.154 | **3x10E$^{-4}$** | **-0.391** |
| | FCSRT free | **0.004** | **0.241** | 0.051 | 0.217 | 0.270 | 0.161 |
| | FCSRT sum | **0.008** | **0.222** | **0.022** | **0.253** | 0.458 | 0.109 |
| **sICAM-1** | MMSE | **0.004** | **-0.168** | 0.096 | -0.154 | 0.698 | -0.057 |
| | Phonetic Fluency | **0.011** | **-0.180** | 0.309 | 0.100 | **0.014** | **-0.273** |
| **sVCAM-1** | MMSE | **0.023** | **-0.133** | 0.106 | -0.150 | 0.639 | -0.045 |
| **C1q** | MMSE | **0.035** | **-0.124** | 0.513 | -0.061 | 0.478 | -0.068 |
| **C3aDesArg** | Semantic Fluency | 0.142 | -0.104 | **0.033** | **-0.208** | 0.103 | -0.180 |
| | Phonetic Fluency | 0.126 | -0.109 | 0.340 | -0.095 | **0.044** | **-0.224** |
| **sIl-1RAcP** | Wordlist Discrimination | 0.379 | 0.063 | **0.048** | **0.193** | 0.426 | 0.090 |
| | TMTA | **0.001** | **0.233** | **0.032** | **0.210** | 0.082 | 0.196 |
| | TMTB | **0.005** | **0.208** | 0.061 | 0.190 | 0.119 | 0.188 |
| **sTREM2** | MMSE | **3x10E$^{-4}$** | **-0.210** | 0.390 | -0.08 | 0.919 | -0.010 |
| | FCSRT free | **0.019** | **-0.196** | 0.479 | -0.079 | 0.161 | -0.203 |

### Table 15: Regression analysis of significant correlations with cognition

Regression analysis for significant correlations between inflammatory markers and NPT results, controllig for the influuence of age, sex and education. Significant p values and associated standardized coefficients β are highlighted bold. Significant associations were predominantly found for phonetic fluency in the AD group.

| Protein | Correlate | Group | Spearman p | Regression p | Regression β |
|---|---|---|---|---|---|
| **MIF** | FCSRT free | AD | 0.001 | 0.078 | -0.264 |
| | FCSRT sum | AD | $3x10E^{-4}$ | **0.001** | **-0.469** |
| **VEGF** | Semantic fluency | AD | 0.015 | **0.022** | **-0.256** |
| | Phonetic fluency | AD | 0.003 | **0.005** | **-0.298** |
| **Il-6** | Figure recall | MCI | 0.048 | 0.468 | 0.069 |
| **Il-8** | FCSRT free | AD | 0.041 | 0.083 | -0.265 |
| | TMTB | MCI | 0.038 | 0.720 | 0.035 |
| **SAA** | Phonetic fluency | AD | 0.001 | 0.055 | -0.205 |
| | TMTA | MCI | 0.025 | **0.039** | **0.197** |
| **CRP** | Phonetic fluency | AD | $3x10E^{-4}$ | **0.007** | **-0.294** |
| | Semantic fluency | AD | 0.036 | 0.131 | -0.176 |
| | FCSRT sum | MCI | 0.022 | 0.161 | 0.160 |
| **sICAM-1** | Phonetic fluency | AD | 0.014 | **0.033** | **-0.248** |
| **C3aDesArg** | Phonetic fluency | AD | 0.044 | **0.025** | **-0.232** |
| | Semantic fluency | MCI | 0.033 | 0.327 | -0.088 |
| **sIl-1RAcP** | Wordlist Discrimination | MCI | 0.048 | 0.096 | 0.165 |
| | TMTA | MCI | 0.032 | **<0.001** | **0.334** |

**Figure 1: Correlation Matrix of MCI and AD cohorts**

The figure is complementary to figure 3 from the main text but depicts the correlation matrix calculated only for the MCI cohort (A) and the AD cohort (B). Most striking were changes in the correlations to NPT results (box 6, which were more pronounced and robust in the AD cohort (compare figure 3 and 4 from main text and table 15 within this supplement).

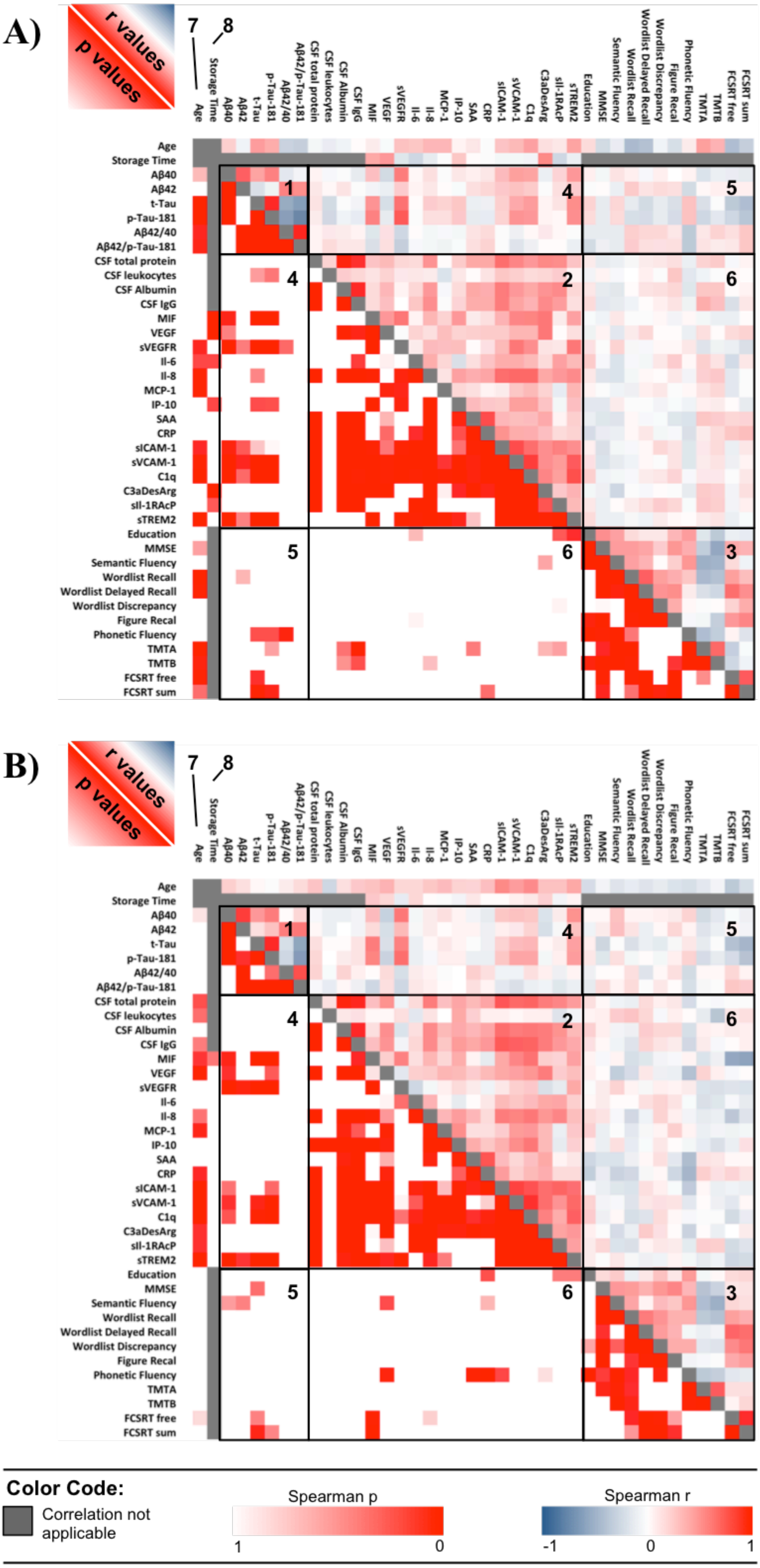
A)
B)
r values
p values
1
2
3
4
5
6
7
8
Age
Storage Time
Aβ40
Aβ42
t-Tau
p-Tau-181
Aβ42/40
Aβ42/p-Tau-181
CSF total protein
CSF leukocytes
CSF Albumin
CSF IgG
MIF
VEGF
sVEGFR
Il-6
Il-8
MCP-1
IP-10
SAA
CRP
sICAM-1
sVCAM-1
C1q
C3aDesArg
sIl-1RAcP
sTREM2
Education
MMSE
Semantic Fluency
Wordlist Recall
Wordlist Delayed Recall
Wordlist Discrepancy
Figure Recal
Phonetic Fluency
TMTA
TMTB
FCSRT free
FCSRT sum
Color Code:
Correlation not applicable
Spearman p
1
0
Spearman r
-1
0
1
